# Construction and pilot test of a set of indicators to assess the implementation and effectiveness of the who safe childbirth checklist

**DOI:** 10.1186/s12884-018-1797-y

**Published:** 2018-05-10

**Authors:** Pedro J. Saturno-Hernández, María Fernández-Elorriaga, Ismael Martínez-Nicolás, Ofelia Poblano-Verástegui

**Affiliations:** Research Center for Evaluations and Surveys (CIEE), National Institute of Public Health of Mexico (INSP), Avenida Universidad No. 655, Colonia Santa María Ahuacatitlán, C.P. 62100 Cuernavaca, Mor Mexico

**Keywords:** Safe Childbirth Checklist, Indicators, Quality improvement, Implementation

## Abstract

**Background:**

The World Health Organization (WHO) launched the “Safe Childbirth Checklist (SCC) Collaboration” in 2012. The SCC is designed to contribute to quality care by providing reminders of evidence-based practices for the prevention and management of the leading causes of maternal and neonatal morbidity and mortality. However, indicators to monitor the implementation and effectiveness of the SCC have not been defined. This study aimed to produce and pilot test a set of valid, reliable and feasible indicators to assess the implementation and effectiveness of the SCC, with an emphasis on best practices.

**Methods:**

As part of the WHO Collaboration, the SCC was adapted to the Mexican context, and a set of indicators was developed to assess the SCC use and adherence to SCC-related best practices. The indicators were pilot tested in three hospitals for feasibility and reliability using the prevalence- and bias-adjusted kappa index (PABAK) for multiple independent evaluators (initial sample, *n* = 47; second sample, *n* = 30 to re-test reliability). The data sources were clinical records and cognitive tests drawn from questionnaires to mothers and health professionals.

**Results:**

We generated 53 indicators, and 38 of the indicators (those related to best practices and outcomes) were pilot tested. Of these, 26 relate to care for the mother (20 were measured based on clinical records and 6 via questionnaire), and 12 relate to newborn care (9 were medical record-based and 3 were from questionnaires). Feasible indicators were generally also reliable (PABAK≥0.6). Routine feasibility is affected by the frequency of assessed events.

**Conclusions:**

The generated indicators allow an assessment of the implementation and effectiveness of the SCC and the monitoring of quality of care during childbirth and the immediate postpartum period.

**Electronic supplementary material:**

The online version of this article (10.1186/s12884-018-1797-y) contains supplementary material, which is available to authorized users.

## Background

Maternal and infant health is a matter of international relevance. There is a broad consensus on the importance of acting around the time of delivery to reduce complications in mothers and newborn babies. It is at this time when there is a higher risk and when the burden of maternal and perinatal death accumulates; most of these deaths occur during the first 24 h after birth [1–3].

Access to professional care at the time of birth, as an isolated strategy without assuring the quality of the care provided, is increasingly considered insufficient to reduce injury to the maternal and child population. Low quality contributes to poor performance [4]. For years, Mexico has promoted institutional childbirth as a strategy to reduce the number and negative impact of complications in mothers and newborns. As a result, by 2012, qualified health professionals attended an average of 99.6% of births [5]. However, there is no correlation between attention to birth in healthcare institutions and the maternal mortality ratio (MMR) [6]. The reported 2015 MMR in Mexico was 38 women per 100,000 live births [7], a figure that did not reach the target MMR of 22 women per 100,000 live births established in the Millennium Development Goals. This number is also far from the MMR of 21 and the infant mortality ratio of 6.5 per 1000 live births in countries of the Organization for Cooperation and Economic Development [8, 9].

With a high rate of access to care at the time of delivery, improving the quality of care that the mother and newborn receive becomes a key strategy for the improvement of health outcomes [10]. To confront this problem, a “Safe Childbirth Checklist” (SCC) with reminders of essential best practices has been generated, and the utilization of the SCC has been tested in a before-after study, in which some essential components included a purposive selection of centers without input problems and the presence of coaches who supervised and observed the SCC use [11].

Based on this promising but difficult to generalize experience, the World Health Organization (WHO) launched the “Safe Childbirth Checklist Collaboration” initiative [12] to obtain evidence regarding the effectiveness and factors associated with the implementation of the SCC. In parallel, in Uttar Pradesh, India, the design and implementation of a cluster-randomized controlled trial to test the impact of the coaching-based use of the SCC on reducing severe maternal, fetal, and newborn harm [13, 14] was also planned. Several countries, including Mexico, are participating in this WHO Collaboration, which will potentially address implementation and effectiveness issues in different contexts using different strategies [15]. Within this international initiative, some studies have recently been published to evaluate the impact of coaching on completing the SCC [16], the level of the SCC use and completion in one tertiary hospital [17], and the improvement in the SCC completion when the SCC is utilized, using Plan-Do-Study-Act (PDSA) cycles in some outcomes [18]. However, a specific comprehensive set of valid and reliable indicators to comparatively evaluate the SCC implementation and the specific good practices promoted by the SCC is still lacking. These indicators are fundamental to understanding the factors associated with SCC implementation and monitoring quality of care [19], the improvements associated with the use of the SCC [20], and eventually the effective implementation of the SCC strategy, thus preventing this strategy from becoming a mere list of intentions [21]. The objective of the present study was to generate a set of indicators, and assess their feasibility and reliability, for routine use to assess the implementation and effectiveness of the SCC, which will primarily be used in the Mexican context but could also be adapted to other countries and contexts.

## Methods

A multi-stage study comprising the following phases was conducted: (i) the adaptation of the SCC to the Mexican context; (ii) the adaptation and development of indicators to evaluate the adherence to best practices related to safe childbirth and the use of the SCC; and (iii) a pilot study to assess the feasibility and inter-rater reliability of the proposed indicator set.

### Adaptation of the SCC to the Mexican context

A working group was established with the participation of the obstetrical, perinatology and quality management staff from four hospitals in the state of Hidalgo, the state of Mexico and Mexico City, supported by a research team from the National Institute of Public Health. The working group adhered to the WHO call for Collaboration on the implementation of the SCC [12]. Following the WHO SCC Collaboration recommendations, the original SCC [22] was refined and adapted to the Mexican context after several iterative discussion sessions. Adaptation was carried out for both content and format. For instance, in relation to format the original WHO SCC is a single document including childbirth and newborn, while our group decided to have separated checklists (one for mother and another for the newborn) to better accommodate the SCC to the structure of hospital childbirth care responsibilities (see online Additional file 1 in Spanish and Additional file 2 in English). In relation to contents, whereas the original SCC asks for simple checks, we added additional information to complete information and reinforce their use as reminder for best practice. For instance, the original SCC ask to check if there was a cesarean section, but in Mexico we added to check on the reason or justification for the cesarean section, according to a list proposed by the working group based on accepted Practice Guidelines.

### Development of indicators

The main basis for the set of indicators related to best practices was the evidence-based structure and process-of-care items contained in the original WHO SCC, converted into indicators. These items and indicators were double-checked and compared with the official recommendations of the Clinical Practice Guidelines in Mexico, to reassure validity and avoid inconsistencies between locally accepted evidence-based practices and the WHO standards for maternal and child care [23]. A group of four researchers with expertise in quality improvement classified the resulting indicators according to type (structure, process, outcome) and potential data source (clinical record or survey) and prepared these indicators for pilot testing by defining the technical specifications of the indicators in a standardized format used previously in other studies [24, 25]. Electronic applications for the computerized capture of data using laptops or tablets were also developed. The detailed definitions of the indicators are available online in Spanish (Additional file 3) and English (Additional file 4). For the indicators regarding the SCC utilization and the factors associated with the SCC, we adapted previous research on the implementation of the Safe Surgery Checklist [26] to the SCC.

### Pilot study for feasibility and reliability

Different strategies were used to measure the indicators depending on the expected data source (clinical records or surveys):

We performed a cross-sectional retrospective study of the indicators that were estimated from clinical records. In two hospitals, systematic random samples were obtained from cases with corresponding ICD-10 codes identified in the computerized system of hospital discharges (SAEH, acronym in Spanish). First, two samples (*n* = 15 each) were obtained from hospital A and were independently assessed by two pairs of evaluators; then, a second sample (*n* = 17) was obtained at hospital B, which was assessed by three evaluators. The seven evaluators did not participate in the construction of the indicators but were trained (10 h) in the use of the indicators and the electronic data capture. An inter-observer reliability analysis was performed by calculating the prevalence-adjusted, bias-adjusted kappa (PABAK) [27] for the pooled sample of 47 cases. An indicator was considered reliable if the PABAK value was ≥0.6, particularly when the observed prevalence was extreme (compliance or non-compliance ≥85%) [28, 29]. The results of this assessment were used to refine the tools and the unreliable indicators. The refined indicators were pilot tested again by four evaluators in a sample of 30 cases (15 cases per couple) in a third hospital. The pilot data were also used to assess the feasibility of measurement. All data were analyzed with the statistical packages Epidat 4.1 [30] and Stata 14.1 [31].

For the indicators obtained from questionnaires, we organized four sessions in two of the participating hospitals following the pre-test cognitive methodology [32], which includes concurrent observation of the behavior of the interviewee and reading/responding aloud. Cognitive methodology was used to ensure respondents understanding of the questionnaire. Reactions and responses were used to change the wording of the questions, when necessary, in order obtain a final version of the questionnaire. The group utilized to test the health personnel questionnaire included 12 professionals. The group for testing the questionnaire for mothers included 15 mothers. Finally, the surveys were fully pilot tested with individual interviews with 5 health professionals and 6 mothers. Two researchers, with previous experience in interviews and surveys, conducted the cognitive sessions and pilot interviews and analyzed the data.

## Results

### Development of indicators

We constructed 53 indicators (Table 1), 15 of which were used to assess the factors associated with utilizing the SCC and the SCC utilization itself (Table 2). The remaining indicators (38), which are described in Tables 3 and 4, were constructed to monitor the impact of the SCC, mainly on good practices but also on outcomes related to complications and adverse events (Table 3). Among these indicators, 25 relate to healthcare for the mother, 11 relate to the newborn and 1 indicator relates to both. Mortality indicators (maternal and neonatal) were excluded from the present pilot study since these indicators are already well established and may be measured from existing databases rather than internally at the institutions using the SCC. The good practice (structure and process) indicators were further classified according to the phases of the SCC: admission and labor, immediately post-partum, and prior to discharge. Indicators that may apply to various stages were grouped in a “general” category.

**Table 1:** Indicators developed to assess the SCC implementation, use and association to good practice and outcomes

Monitoring Objective	Type of indicator			Total
	Structure	Process	Outcome	
Factors associated to the SCC implementation	11	–	–	**11**
Utilization of the SCC	–	4	–	**4**
Good practices	2	26	–	**28**
Health outcomes	–	–	10	**10**
Total	**13**	**30**	**10**	**53**

**Table 2:** Indicators developed to assess the SCC utilization and factors potentially associated to SCC implementation

	Data source
A. Indicators potentially associated to the SCC implementation	
Training on the SCC	
1. Professionals trained in the use of the SCC	Training records/Questionnaire to health professionals
2. Professionals trained in the use of the SCC by professional profile	Training records/Questionnaire to health professionals
Characteristics of the team	
3. Team size	Log books/Questionnaire to health professionals
4. Professional profile of team members	Log books/Questionnaire to health professionals
5. Availability of staff trained in neonatal resuscitation (apply to all shifts)	Questionnaire to health professionals
6. Composition of the team that attends the childbirth (pre-expulsive stage)	Log books/Observation/Questionnaire to health professionals
7. Presence of personnel skilled in neonatal resuscitation at birth	Log books/observation/questionnaire to health professionals
Attitude of health professionals	
8. Perception of the usefulness of the SCC (not a waste of time)	Questionnaire to health professionals
9. Perception of the impact of the SCC	Questionnaire to health professionals
10. Perception of the importance of the SCC implementation for the hospital	Questionnaire to health professionals
SCC Availability	
11. Availability of SCC formats when needed	Questionnaire to health professionals
B. SCC utilization	
12. Percentage of deliveries with SCC (presence in the medical record)	Medical records
13. Percentage of deliveries with completed SCC (all items)	SCC present in medical records
14. Average percentage of SCC completed items (global)	SCC present in medical records
15. Average percentage of SCC completed items by childbirth stage.	SCC present in medical records

**Table 3:** Good practice indicators measured from medical records. Feasibility and reliability from two sequential pilot tests

Indicator description		Prevalence and adjusted bias kappa (PABAK) Pilot test 1			PABAK Pilot test 2
		Hospital 1 (n:30)	Hospital 2 (n:17)	Pooled results Hospitals 1 and 2	Hospital 3 (n:30)
1	Percentage of women with antibiotic prescribed during the childbirth process	0.53	0.88	0.66	0.50
2	Percentage of women with antibiotic prescribed during childbirth process antibiotic and it is justified by any symptom	0.6	0.41	0.49	*NF*
3	Percentage of newborns who are prescribed antibiotic	0.67	1	0.79	1
4	Percentage of newborns who are prescribed antibiotic and it is justified by any symptom	*NF*	*NF*	*NF*	*NF*
5	Percentage of women who are prescribed magnesium sulfate during the childbirth process	1	1	1	1
6	Percentage of women who are prescribed magnesium sulfate during the childbirth process and it is justified	*NF*	*NF*	*NF*	*NF*
7	Percentage of women with partogram open, including:	0.38	1	0.61	1
	• Partograms with name recorded	0.59	1	0.74	1
	• Partograms with age or date of birth recorded	0.59	0.76	0.65	1
	• Partograms with weeks of gestation recorded	0.59	0.76	0.65	1
8	Percentage of women with completed partogram, including:	1	1	1	0.22
	• Partograms with temperature recorded every 2 h	0.52	0.76	0.61	0.48
	• Partograms with heart rate recorded every 30 min	0.93	1	0.96	0.13
	• Partograms with blood pressure recorded every 4 h	0.10	0.76	0.35	0.57
	• Partograms with registration of vaginal exam or dilation	0.86	0.41	0.70	0.91
9	Percentage of women HIV+ with initiation of anti-retroviral treatment	*NF*	*NF*	*NF*	*NF*
10	Percentage of women with justified Cesarean delivery	*NF*	*NF*	*NF*	0.83
11	Percentage of women with justified instrumental delivery	*NF*	*NF*	*NF*	*NF*
12	Percentage of women with justified episiotomy	0.92	*NF*	0.92	0.90
13	Percentage of women with adequate management of the 3rd period of delivery, including:	0.87	1	0.96	0.78
	• Deliveries with oxytocin administered in the first minute	0.33	0.29	0.32	0.43
	• Births with traction of the umbilical cord to extract the placenta	0.20	−0.88	−0.17	0.85
	• Uterine massage is performed after removing the placenta	0.87	0.88	0.87	0.93
14	Percentage of deliveries in which the presence of a second baby is checked and disconfirmed	0.20	0.65	0.36	1
15	Percentage of women with adequate management of postpartum hemorrhage	*NF*	*NF*	*NF*	*NF*
16	Percentage of newborns with adequate immediate care, including:	0.72	0.88	0.82	0.73
	• Drying and kept warm	0.24	0.76	0.43	0.73
	• Administration of vitamin K	0.71	1	0.82	1
	• Administration of ophthalmic prophylaxis	0.86	0.88	0.87	0.8
17	Percentage of newborns with late clamping of umbilical cord	0.87	0.53	0.74	1
18	Percentage of newborns with immediate skin to skin contact	0.72	0.06	0.48	1
19	Percentage of newborns with HIV+ mothers and anti-retroviral treatment initiated	*NF*	*NF*	*NF*	*NF*
20	Percentage of women with hemorrhage	0.87	0.88	0.87	0.93
21	Percentage of women with blood pressure disorders	0.93	0.88	0.91	0.93
22	Percentage of women with post-partum or perinatal infection	0.87	1	0.91	0.71
23	Percentage of infants with neonatal infection	0.8	1	0.87	1
24	Percentage of infants with neonatal asphyxia	0.93	1	0.96	0.87
25	Percentage of women with Cesarean delivery	1	1	1	1
26	Percentage of women with instrumental delivery	1	1	1	1
27	Percentage of women with episiotomy at childbirth	1	1	1	0.88
28	Percentage of deliveries with adverse events in women	0.8	0.88	0.83	1
29	Percentage of deliveries with adverse events in newborns	1	1	1	0.60

*NF* Not feasible due to few or lack of cases in the general sample: it may need specific sample for routine use

General indicators: #1 to 6 (applicable at any time during childbirth); Admission and labor: # 7 to 15; Newborns (immediate post-partum): #16 to 19; Complications and adverse events: #20 to 29

**Table 4:** Good practice indicators measured with surveys to mothers or health professionals

Indicator description		Type of indicator	Data source
1	Percentage of women whose partner is informed and encouraged to be present at the birth	Process	Questionnaire to mothers
2^a^	Frequency of availability of inputs for attention to the mother immediately before childbirth	Structure	Questionnaire to health professionals
3^a^	Frequency of availability of inputs for care of the newborn immediately after childbirth	Structure	
4^b^	Percentage of newborns with immediate skin to skin contact	Process	Questionnaire to mothers
5^b^	Percentage of newborns starting breastfeeding right after birth	Process	
6	Percentage of women informed about family planning before discharge	Process	
7	Percentage of women and/or partners informed about the mother warning signs to ask for help	Process	
8	Percentage of women who know at discharge the visits that must be made (them and the newborn) and the place to turn to revisions	Process	
9	Percentage of women and/or partners informed about the warning signs of the newborn to ask for help	Process	

^a^They could be measured also by observation; ^b^They could be measured also from medical records

### Pilot test of the indicators

#### Reliability of the feasible indicators obtained from clinical records

Table 3 describes the feasibility and reliability results of the indicators measured using data registered in clinical records in phases 1 and 2 of the pilot test. To better reproduce an actual situation that may be encountered in most facilities willing to implement the SCC and monitor its impact on good practices, all deliveries were sampled; no samples were obtained according to particular pathologies or specific events, which in most cases may not be coded in the discharge database. For this reason, 6 of the 29 indicators could not be assessed for reliability due to the low number of cases with the required condition in the general samples. Purposive (usually not routinely feasible) or much larger samples may be required to monitor these indicators. Reliability could not be assessed for this reason in cases of newborns with conditions justifying the prescription of antibiotics or the adequate prescription of magnesium sulfate to mothers or for HIV+ women for whom anti-retroviral treatment was prescribed for both the mother and the newborn. The lack of routine feasibility also affected two other indicators (the justification for prescribing antibiotics to mothers and the justification of a cesarean section) in some of the three hospitals. In these cases, incomplete or confusing medical records made the assessment of the reliability and the actual measurement of the indicators unfeasible.

Most of the indicators exhibited either confirmed or improved reliability in the second pilot test, after refining the description of the indicators based on the results of the first pilot test. Only one indicator (deliveries with oxytocin administered during the first minute) was not adequately improved, and another indicator (the composite indicator reflecting the completeness of the partogram) had a worse PABAK value in the third hospital, related to difficulties in accurately assessing the time intervals of recording temperature (in the mother) and heart rate (in the fetus). All outcome indicators, mostly related to adverse events or complications in either the mother or the newborn, were feasible and reliable.

#### Indicators obtained from questionnaires

Short questionnaires for mothers and health professionals were proposed as data sources for nine relevant indicators (Table 4). Three of these indicators (the percentage of newborns with skin-to-skin contact immediately after birth, breastfeeding right after birth, and the percentage of mothers with information on family planning prior to discharge) may theoretically be obtained from either the clinical record or questionnaires to mothers. However, we found the questionnaire more reliable. As an example, “skin-to-skin contact” was not registered in any of the reviewed clinical records, while mothers understood the question perfectly well in the cognitive test of the questionnaire, thereby providing this relevant information. Furthermore, as an alternative to the periodic inspection of the facilities or the observation of a sample of childbirths, we found that health professionals may be a good data source for a quick assessment of the indicators related to the availability of inputs regarding the attention to the mother during labor and regarding the newborn immediately after childbirth. The other four indicators in this group reflect aspects of the experience of the mother that could be measured only by asking these women.

## Discussion

The “Safe Childbirth Checklist Collaboration” intiative [12] was organized by the WHO with the objectives of identifying the factors that may influence the SCC implementation, better defining a strategy for the effective utilization of the SCC, and gathering data regarding the impact on the quality of care provided to mothers and newborns, with the ultimate goal of improving health outcomes for both. As part of this collaboration, we adapted the SCC to a Mexican context and defined and pilot tested a set of indicators to assess both the SCC implementation process and the impact on best practices and outcomes. The indicators were based on previous research on the implementation of the Safe Surgery Checklist [26] and the evidence-based good practices included in the SCC [1, 11]. The constructed set consists of 53 indicators: 15 indicators related to the implementation of the SCC and 38 indicators involving SCC-related good practices and outcomes. After two sequential pilot tests in three hospitals, we found feasibility problems in six of these indicators and insufficient reliability for two indicators. The considerations described below reflect the knowledge gained from our study and emphasize the importance of systematic measurement of SCC implementation to compare results both within and between healthcare units and to gather evidence on the effectiveness of the SCC and its associated implementation factors.

### The need for valid and reliable indicators linked to the SCC

The SCC includes reminders for interventions with proven positive impacts on quality childbirth and the management of complications and mortality in both the mother and the newborn, but the SCC was launched without identifying indicators that may be used to monitor the utilization and impact of the SCC. However, valid measures allow the monitoring of processes and results of strategies, thereby evaluating the strategies and facilitating decision making [33]; monitoring is necessary for the effective implementation of a strategy and contributes to preventing the strategy from becoming a mere list of intentions [21]. Measuring and reporting results is also important for accountability and transparency [33]. In recent years, particularly after the completion of the Millennium Development Goals, there has been an emphasis on the design of valid strategies and measuring instruments to monitor interventions and enable comparisons at the national and international levels [20, 23, 33]. To better analyze the attributed impact and eventual implementation strategies, we believed that a set of specific indicators closely related to the SCC content was required to evaluate the SCC impact, along with measures of SCC utilization. To date, the largest study on the implementation of the SCC, the Better Birth Trial, assessed the SCC use in 18 of the 39 best practices for which the SCC provided reminders and was based on the comparative observation of coaches and independent observers when coaches were not present [16]. The main objective was to assess the impact of coaching on SCC use, and the analyzed practices were therefore those that could be observed by either coaches or independent observers when the coaches were not present. No attempt was made to provide indicators that could be used to assess the SCC strategy as such, including factors other than coaching that may influence the SCC use. Other studies [17, 18] primarily focused on the completion of the SCC when used, excluding from the analysis all cases in which the SCC was not utilized.

### Pilot test reveals reliability and context-related difficulties

Our proposed indicators are intended for use in monitoring the implementation and effectiveness of the SCC, allowing for comparisons among cases and also institutions where the SCC has not been used. Therefore, the target population was total deliveries, and the data sources were not only those cases in which the SCC was utilized. The numerator and denominator, the terms used and proposed data sources may thus be carefully defined. However, regardless of how well-defined the terms and data sources of an indicator are, pilot testing in actual environments is necessary to guarantee the feasibility of a measurement, an unambiguous interpretation and an eventual technical refinement to increase reliability. A pilot test may reveal that the specifications must be more detailed or that the data may not be available [34–36]. Skipping this step may result in proposing unfeasible or unreliable, and therefore not valid, indicators [36]. We observed all of these types of problems. In some cases, we discussed and refined the specifications or the seemingly unreliable indicators based on the PABAK value obtained in the first pilot test, and in other cases, we highlighted problems with the data source that may affect the generalization of the proposed measures. It was most difficult to find correctly recorded time specifications, and in some cases, we changed specifications to include different ways in which such recorded data could actually be compliant but disparate. For example, “oxytocin administered during the first minute” was sometimes recorded as “oxytocin given after the liberation of shoulders” or “was given oxytocin immediately after the release of the product”; for this reason, we included these response options as valid compliance in the indicator description. However, the majority of feasibility problems related to deficiencies in the data sources, including the identification of cases for assessment and the quality of the recorded information in clinical records and other existing documents, such as partograms, which underscores the importance of context-specific obstacles to monitoring otherwise potentially valid indicators. In relation to the identification of cases (i.e., deliveries) in the routine hospital discharge system, we observed that in many cases, deliveries were not coded as such; the existence of any previous condition or complication as the cause of hospitalization was preferred in many cases. We also identified (and propose to identify) childbirths by searching individual records for other variables that are related to childbirths, such as the gestational age, in combination with the recording of a birth product. There were women with “gestational age” and “birth products” not coded as deliveries.

After selecting cases, extracting the necessary information may also be complex. The lack of uniformity in the formats of medical records is a major contributor to the confusion. Many times, the required information could be recorded in different places or formats, and it is necessary to clarify what format should be revised for each variable. For instance, gestational age may not be in the medical record but may instead be in the partogram. In some cases, when the same information was recorded in different formats, it was possible to detect inconsistencies (even in relation to simple variables, such as the sex of the newborn or the type or birth, vaginal or cesarean) that contributed to context-specific causes of the lack of data reliability.

These findings from the first pilot study in two hospitals and the entire process of refining the indicators were fundamental to obtaining more reliable tools, which were tested again in a second pilot study in a third hospital. However, two of the proposed indicators, both related to the recording of the appropriate time in which the required actions should be performed, had questionable reliability results in the second pilot study. One of these indicators, related to the contents of the partogram, showed reliability in the first pilot study. This discrepancy suggests the potential need for pilot testing in a given environment, even for indicators that are reliable in other environments. Such a pilot test can be performed with small sample sizes [36], but for national or regional projects, a period of monitoring data quality and availability as well as designing strategies to generate timely and reliable data have been suggested [37, 38] .

### The need for questionnaires and purposive sampling for comprehensive SCC monitoring

Some of the SCC items, although relevant, apply to sub-populations of mothers and newborns defined by a given condition. Routine monitoring of these indicators would not be possible unless the information system allows for the easy identification of cases with that particular condition, given the relatively low frequency in which these cases may occur. We classified these indicators, a total of six indicators, as routinely non-feasible. The timely initiation of antiretroviral treatment for HIV+ women and their newborns is a clear example of this type of indicator, which applies only to the subpopulation of known HIV+ women. The same situation occurs with other cases that cannot be readily identified in the discharge information system, such as the justification of antibiotic treatment, the justified administration of magnesium sulfate, and even the adequate management of postpartum hemorrhage. Larger or specific purposive samples, in cases in which these situations could be identified in routine databases, may be required to measure these indicators. In other SCC studies, based only on those cases in which the SCC was utilized and using the SCC checked items as the data source [17, 18], these indicators, particularly those related to HIV+ women and their newborns, show some of the lowest compliance (compliance meaning checking the item in the SCC). In other cases, such as the in the study by Spector JM et al. [11], it is not clear whether compliance includes checking the item defining the sub-population (i.e., assessing HIV status) plus giving the treatment as an “all-or-none” composite indicator. In addition, in our study, the SCC adaptation distinguished two steps: one, whether a treatment or intervention has been provided (for instance, antibiotics, magnesium sulfate, or episiotomy); and two, the explicit justification for that treatment or intervention. Monitoring the quality of the second step in these practices may not be routinely feasible unless the prevalence of the subpopulation to which they apply is sufficiently high in a routine sample of childbirths. The original SCC simply asks whether the particular treatment or intervention is needed and has been administered (in a single Yes/No item) and then provides some advice (a short list of symptoms or situations) regarding the indications for the treatment. In our adaptation, the group of experts considered it more appropriate to transform these items into two companion and sequential items: first, checking whether the treatment was administered (Yes/No), and then, checking the symptom or situation that justifies the treatment. In this way, the presence of any of these symptoms may also be an explicit reminder for considering the treatment if it has not been administered; at the same time, these questions may be used to assess adequacy.

However, there are important aspects (skin-to skin-contact, the initiation of breastfeeding, information about warning signs, among others) for which we could not expect good registered data. The same situation occurred in the search for potential factors that may affect the SCC use, namely those related to the attitudes of health personnel, the existence of inputs and other structural factors. For both types of measures, we devised and successfully tested short questionnaires for mothers and health personnel. Without these additional tools, a comprehensive monitoring and evaluation of the use and impact of the SCC and the subsequent design of improvement initiatives would not be possible.

The technical specifications of the proposed set (Additional file 1 in Spanish and Additional file 2 in English) are consistent with the findings of the pilot tests and include the proposed data source, making these indicators ready for use in the Mexican environment and also for adaptation to other environments.

## Limitations

The present study was conducted using general samples of hospital deliveries. No attempt was made to find samples of subpopulations or specific conditions in mothers or newborns not included as variables in the discharge databases. A comprehensive SCC evaluation may require larger or, if feasible, purposive samples, but our intention was to test the set for potential routine use. The results may be context-specific, and the use of the proposed set in other contexts may require local adaptation to the particular information systems. Nevertheless, the proposed set would be a good starting point, given that this set reflects the relevant SCC items and the main factors that could potentially be associated with the SCC implementation.

## Conclusions

We constructed and pilot tested a set of indicators for feasibility and reliability in monitoring the implementation and impact of the WHO SCC. Technical specifications were modeled after the SCC items, previous research on the use of checklists, the results of two pilot tests, and the context of Mexico, but the specifications could be used in other contexts after local adaptation and analysis of the available data sources.

## Additional files

Additional file 1: Mexico Safe Childbirth Checklist for mother and newborn (spanish). The original WHO Safe Childbirth Checklist was refined and adapted to the Mexican context. (PDF 5695 kb)

Additional file 2: Mexico Safe Childbirth Checklist for mother and newborn (english). The original WHO Safe Childbirth Checklist was refined and adapted to the Mexican context and translated to english language. (PDF 458 kb)

Additional file 3: Set of SCC indicators (spanish version). The detailed definitions of the indicators in Spanish. (DOCX 116 kb)

Additional file 4: Set of SCC indicators (english version). The detailed definitions of the indicators in English. (DOCX 139 kb)

## Abbreviations

MMR: Maternal mortality ratio
PABAK: Prevalence-adjusted, bias-adjusted kappa
P-D-S-A cycle: Plan-Do-Study-Act (PDSA) cycles
SAEH: Computerized system of hospital discharges (acronym in Spanish)
SCC: Safe Childbirth Checklist
WHO: World Health Organization
