# Supplementary material for: Construction and pilot test of a set of indicators to assess the implementation and effectiveness of the who safe childbirth checklist
Source: BMC Pregnancy Childbirth. 2018 May 10;18:154. doi: 10.1186/s12884-018-1797-y (PMC5946578; doi:10.1186/s12884-018-1797-y)
Supplement: Supplementary file 1 — Mexico Safe Childbirth Checklist for mother and newborn (spanish). The original WHO Safe Childbirth Checklist was refined and adapted to the Mexican context. (PDF 5695 kb) [file 12884_2018_1797_MOESM1_ESM.pdf]

Organización Mundial de la Salud

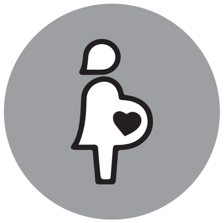

LISTA DE VERIFICACIÓN DE PARTO SEGURO

Folio ______________________

OMS

MADRE	
Nº de expediente clínico	
Nombre	
Edad	
Localidad	
Escolaridad	
Idioma/s	
Nº de embarazos	
Nº de hijos	

RECIÉN NACIDO	
Nombre	
Fecha de nacimiento	
Edad gestacional al nacimiento	
Peso al nacer	
Longitud al nacer	
Apgar 1	Apgar 5

HOSPITAL	
Nombre	
Localidad	
Responsable del servicio	
Responsable de la SCC	

ETAPA EN LA QUE LLEGA LA MADRE (marcar una opción)

- ☐ En la admisión/previa al alumbramiento
- ☐ Justo antes de pujar (o de cesárea)
- ☐ Justo después de dar a luz (durante la primera hora post-parto)
- ☐ Antes del egreso/Alta

MOTIVO DE EGRESO

☐ Referencia ☐ Alta ☐ Muerte

LISTA DE VERIFICACIÓN DE PARTO SEGURO PARA MÉXICO - EDICION PILOTO

OMS

En la admisión / previo al alumbramiento — MADRE

¿Ha llevado la mujer control prenatal?

- ☐ No, calificar el riesgo
- ☐ Si

Revisar
☐ Exámenes actualizados de: grupo y Rh, hemograma,VIH, VDRL/RPR, orina, bioquímica y ecografia.

¿Existe necesidad de referencia de la mujer?

- ☐ No
- ☐ Si

Señalar la causa y habilitar el protocolo:
☐ Materna
☐ Fetal
☐ Obstétrica

¿Se ha realizado apertura del partograma en el ingreso?

- ☐ No
- ☐ Si

Vigilar y registrar:
☐ Tacto/dilatación según evaluación e indicación médica
☐ Cada 30 min: frecuencia cardiaca de la mujer y del feto, y las contracciones.
☐ Cada 2 horas: temperatura.
☐ Cada 4 horas: presión arterial

¿Necesita la madre comenzar tratamiento con... ?

Antibióticos

- ☐ No
- ☐ Si, administrado

Señalar la causa:
☐ Temperatura > 38°
☐ Flujo con olor desagradable (fétido)
☐ Ruptura de membranas > 18 horas
☐ En trabajo de parto > 24 horas
☐ Otras ______________________________

Antihipertensivo

- ☐ No
- ☐ Si

Nombre del medicamento ________________

Sulfato de magnesio

- ☐ No
- ☐ Si, administrado

En este caso señalar la causa:
☐ Presión diastólica ≥110 mm/Hg y 3+ proteinuria
☐ Presión diastólica ≥90 mm/Hg, 2 + proteinuria, y cualquiera de : cefalea severa o visión borrosa
☐ Dolor epigástrico (descartar hematoma hepático no roto)
☐ Excepción: Insuficiencia renal, lesión miocárdica, bloqueo cardiáco, miastenia gravis.
☐ Otros ______________________________

En la admisión / previo al alumbramiento — MADRE

¿Necesita la madre comenzar tratamiento anti-retroviral?

- ☐ No
- ☐ Si, administrado

Mujer con VIH + y en trabajo de parto.
Nombre del medicamento________________

Disponibilidad de insumos para el lavado de manos y uso de guantes para cada exploración vaginal:

- ☐ No
- ☐ Si

Se ofrece, informa y anima al acompañante para estar presente en el parto:

- ☐ No
- ☐ Si

Confirmar que la mujer y/o el acompañante han sido informados sobre los signos de alarma ante los que pedir ayuda:

- ☐ No
- ☐ Si

Señalar cuales conocen:
☐ Sangrado
☐ Sensación de urgencia de pujar
☐ Intenso dolor abdominal
☐ Disminución de movimientos fetales
☐ Intenso dolor de cabeza
☐ Alteraciones visuales
☐ Pérdida de líquido trans-vaginal
☐ Alteraciones cognitivas

¿Se permite la ingesta de líquido durante el trabajo de parto?

- ☐ No
- ☐ Si

¿Se permite posición libre para el parto?

- ☐ No
- ☐ Si

Completado por: Nombre ______________________________
Cargo/Puesto

LISTA DE VERIFICACIÓN DE PARTO SEGURO PARA MÉXICO - EDICION PILOTO

OMS

Justo antes de pujar (o de cesárea) — MADRE

¿Presenta la madre indicación de cesárea?
- ☐ No
- ☐ Si

Se realiza segunda opinión y señalar la causa:
- ☐ 2 cesáreas previas
- ☐ situación transversa
- ☐ embarazo gemelar
- ☐ presentación pélvica
- ☐ cardiopatía clase III y IV
- ☐ hidrocefalia fetal
- ☐ antecedente de cirugía uterina
- ☐ tumor que obstruya el canal de parto
- ☐ desprendimiento prematuro de placenta normoinserta
- ☐ producto óbito mayor de 30 SDG en paciente sin trabajo de parto por más de 24 horas.
- ☐ placenta previa total
- ☐ cerclaje vía abdominal
- ☐ macrosomía
- ☐ estado fetal inestable
- ☐ malformaciones fetales
- ☐ herpes genital activo
- ☐ VIH

¿Presenta la madre indicación de parto instrumentado?
- ☐ No
- ☐ Si

Señalar la causa:
- ☐ cesárea previa
- ☐ expulsivo con inestabilidad fetal
- ☐ cardiopatía materna
- ☐ agotamiento materno
- ☐ Otra________________________________

¿Presenta la madre indicación de episiotomía?
- ☐ No
- ☐ Si

Causa________________________________

¿Necesita la madre comenzar tratamiento con...?

Antibióticos
- ☐ No
- ☐ Si, administrado

Señalar la causa:
- ☐ Temperatura > 38º
- ☐ Ruptura de membrana > 18 horas
- ☐ Flujo con olor desagradable
- ☐ En el trabajo de parto > 24 horas
- ☐ Inicio de cesárea
- ☐ Otras________________________________

Antihipertensivo
- ☐ No
- ☐ Si

Nombre del medicamento________________

Sulfato de magnesio
- ☐ No
- ☐ Si, administrado

Señalar la causa:
- ☐ Presión diastólica ≥110 mm/Hg y 3+ proteinuria
- ☐ Presión diastólica ≥90 mm/Hg, 2+ proteinuria, y cualquiera de: cefalea severa o visión borrosa
- ☐ Dolor epigástico (descartar hematoma hepático no roto)
- ☐ Excepción: Insuficiencia renal, lesión miocárdica, bloqueo cardiáco, miastenia gravis.
- ☐ Otros ____________________________

Disponibilidad de insumos para atención de la madre inmediatamente antes del parto:
- ☐ No
- ☐ Si

- ☐ Guantes
- ☐ Uterotónico: Oxitocina 10 IU en jeringa
- ☐ Jabón y agua limpia
- ☐ 2 pinzas de anillos

¿Se ha identificado a un asistente para el parto y está informado de que debe estar listo para prestar ayuda en cualquier momento del parto?
- ☐ No
- ☐ Si

Nombre ____________________________

Completado por: Nombre ____

Cargo/Puesto

Justo después de dar a luz (durante la primera hora posterior al parto) — MADRE

Atención a la madre inmediatamente después del nacimiento:
- ☐ No
- ☐ Si

- ☐ Descartar presencia de un segundo producto
- ☐ Administración de Oxitocina en el primer minuto
- ☐ Controlar tracción del cordón umbilical para la extracción de la plancenta
- ☐ Masaje de útero después de extraer la placenta

¿Está la madre sangrando demasiado?
- ☐ No
- ☐ Si, pedir ayuda

Acciones en post parto si hay hemorragia ≥ 500 mL, o si ≥ 250 mL y anemia severa:
- ☐ Masaje al útero
- ☐ Iniciar vía intravenosa
- ☐ Considerar uterotónico adicional
- ☐ Activar equipo de respuesta rápida para emergencias obstétricas
- ☐ Tratar la causa

¿Necesita la madre comenzar tratamiento con?

Antibióticos
- ☐ No
- ☐ Si

Causa ________________________________

Antihipertensivo
- ☐ No
- ☐ Si

Nombre del medicamento ________________

Sulfato de magnesio
- ☐ No
- ☐ Si, administrado

En este caso señalar la causa:
- ☐ Presión diastólica ≥110 mm/Hg y 3+ proteinuria
- ☐ Presión diastólica ≥90 mm/Hg, 2+ proteinuria, y cualquiera de: cefalea severa o visión borrosa
- ☐ Dolor epigástrico (descartar hematoma hepático no roto)
- ☐ Excepción: Insuficiencia renal, lesión miocárdica,bloqueo cardiáco, miastenia gravis.
- ☐ Otros ____________________________

Confirmar que la mujer y/o el acompañante han sido informados de los signos de alarma ante los que pedir ayuda
- ☐ No
- ☐ Si

Señalar cuales conocen:
- ☐ Sangrado
- ☐ Fuerte dolor abdominal
- ☐ Fuerte dolor de cabeza
- ☐ Alteraciones visuales
- ☐ Dificultad respiratoria
- ☐ Dificultad para vaciar la vejiga
- ☐ Fiebre y/o escalofríos

Completado por: Nombre ____

Cargo/Puesto

Antes del egreso / Alta — MADRE

¿Está el sangrado de la madre controlado?

- ☐ No: tratar y diferir el alta
- ☐ Si

¿Necesita la madre comenzar tratamiento con antibióticos?

- ☐ No
- ☐ Si, administrado

Señalar la causa:
- ☐ Temperatura >38° C y: escalofrío o flujo con olor desagradable o bajo tono/distensión abdominal
- ☐ Otras_______________________________

¿Se le ha ofrecido a la madre información sobre planificación familiar antes de su egreso?

- ☐ No: dar orientación y consejería
- ☐ Si

Confirmar que la mujer y/o el acompañante han sido informados de los signos de alarma ante los que pedir ayuda:

- ☐ No
- ☐ Si, administrado

Señalar cuales:
- ☐ Sangrado
- ☐ Alteración del estado de conciencia
- ☐ Dolor abdominal intenso
- ☐ Alteraciones visuales
- ☐ Dolor de cabeza intenso
- ☐ Dificultad respiratoria
- ☐ Dificultad para vaciar la vejiga
- ☐ Fiebre y/o escalofríos

Si la madre es VIH + ¿madre y recién nacido tienen antirretrovirales para el tratamiento completo (1 mes el recién nacido y la madre continuación con su seguimiento)?

- ☐ No
- ☐ Si, administrado

¿Se ha organizado y acordado el seguimiento de la madre y el RN?

- ☐ No
- ☐ Si

Completado por: Nombre ______________________________

Cargo/Puesto

GOBIERNO DEL ESTADO DE MÉXICO

GENTE QUE TRABAJA Y LOGRA enGRANDE

ISEM

Hospital General de Toluca Dr. Nicolás San Juan
Hospital General de Ecatepec Dr. José María Rodríguez

HIDALGO TIERRA DE TRABAJO — SECRETARÍA DE SALUD

Estado Libre y Soberano de Hidalgo

Hospital Obstétrico de Pachuca
Hospital General de Tula

INPer — Instituto Nacional de Perinatología Isidro Espinosa de los Reyes

Instituto Nacional de Salud Pública

CONACYT — Consejo Nacional de Ciencia y Tecnología

Organización
Mundial de la Salud

LISTA DE VERIFICACIÓN
DE PARTO SEGURO

LISTA DE VERIFICACIÓN DE PARTO SEGURO PARA MÉXICO - EDICION PILOTO

OMS

Folio ____________________

RECIÉN NACIDO

Nombre	
Edad gestacional al nacimiento	
Peso al nacer	
Longitud al nacer	
Apgar 1	Apgar 5

MADRE

Nº de expediente clínico	
Nombre	

MOTIVO DE EGRESO

☐ Referencia ☐ Alta ☐ Muerte

Justo antes de pujar (o de cesárea) — RECIÉN NACIDO

Disponibilidad de insumos para atención del recién nacido:

☐ No
☐ Si

☐ Toallas limpias
☐ Tijera para corte de cordón
☐ Perilla de succión
☐ Vitamina K
☐ Profilaxis oftálmica
☐ Equipo de canalización
☐ Laringoscopio
☐ Cinta/pinza para cordón umbilical
☐ Fuente de O_2 y aspiración
☐ Adrenalina
☐ Cánulas de intubación endotraqueal
☐ Solución salina
☐ Solución glucosada
☐ Mascarilla y bolsa para ventilación (ambú)

¿Está presente algún profesional con capacitación actualizada en reanimación neonatal?

☐ No
☐ Si

Nombre ____________________

Completado por: Nombre ____________________
Cargo/Puesto ____________________

LISTA DE VERIFICACIÓN DE PARTO SEGURO PARA MÉXICO - EDICION PILOTO

OMS

Justo después de dar a luz (durante la primera hora posterior al parto) — RECIÉN NACIDO

Cuidado del recién nacido:

Secar al recién nacido
- ☐ No
- ☐ Si

Mantenerlo caliente
- ☐ No
- ☐ Si

Vitamina K
- ☐ No
- ☐ Si

Profilaxis oftálmica.
- ☐ No
- ☐ Si

Pinzamiento tardío de cordón umbilical
- ☐ No
- ☐ Si

Causa ______________________________

¿Se comienza con el contacto piel con piel?
- ☐ No
- ☐ Si

Causa ______________________________

¿Se comienza con lactancia materna?
- ☐ No
- ☐ Si

Causa ______________________________

¿Necesita el recién nacido cuidado especial y monitorización?
- ☐ No
- ☐ Si, administrado

Señalar la causa:
- ☐ Requirió reanimación
- ☐ Nacimiento más de un mes antes
- ☐ Hipoglucemia
- ☐ Necesidad de antibiótico
- ☐ Dificultad respiratoria
- ☐ Peso <2500 gramos

Justo después de dar a luz (durante la primera hora posterior al parto) — RECIÉN NACIDO

¿Existe necesidad de referencia del recién nacido a unidad de cuidados especiales?
- ☐ No
- ☐ Si, organizado

Señalar la causa:
- ☐ Sospecha de sepsis
- ☐ Dificultad respiratoria
- ☐ Sospecha de patología neonatal (cual):

____

¿Necesita el recién nacido comenzar tratamiento con antibióticos?
- ☐ No
- ☐ Si, administrado

Señalar la causa:
- ☐ Respiración rápida (>60 respiraciones/min) o lenta (< 30/min)
- ☐ Pecho sumido, tiraje intercostal, ruido, convulsiones
- ☐ Corioamniotis
- ☐ Movilidad escasa o nula a la estimulación
- ☐ Se le dió a la madre
- ☐ Ruptura prematura de membranas (>18 horas)
- ☐ Temperatura muy fría (<35° y no se calienta) o temperatura alta (>38° C)
- ☐ Otras: ______________________________

¿Necesita el recién nacido comenzar tratamiento anti-retroviral?
- ☐ No
- ☐ Si

Administrado en las primeras 6 horas si la madre presenta VIH+.
Nombre del medicamento:

____

Confirmar que la mujer y/o el acompañante han sido informados de los signos de alarma ante los que pedir ayuda:
- ☐ No
- ☐ Si

Señalar cuales conoce:
- ☐ Ictericia
- ☐ Fiebre
- ☐ Crisis convulsivas
- ☐ Respiración rápida o dificultad al respirar
- ☐ Extremadamente frío
- ☐ Interrupción de la correcta alimentación
- ☐ Menos actividad de lo normal

Completado por: Nombre ______________________________

Cargo/Puesto ______________________________

LISTA DE VERIFICACIÓN DE PARTO SEGURO PARA MÉXICO - EDICION PILOTO

OMS

Antes del egreso / Alta — RECIÉN NACIDO

¿Necesita el recién nacido comenzar tratamiento con antibióticos?

☐ No
☐ Si. Administrar, diferir el alta y dar cuidados especiales o referir:

Señalar la causa:
☐ Se le dio a la madre
☐ Respiración rápida (>60 respiraciones/min) o lenta (<30/min)
☐ Corioamniotis
☐ Pecho sumido, tiraje intercostal, ruido, convulsiones
☐ Movilidad escasa o nula a la estimulación
☐ Ruptura prematura de membranas (>18 horas)
☐ Temperatura muy fría (<35°C y no se calienta) o temperatura alta (>38°C)
☐ Otras________________________________

¿Presenta el recién nacido ictericia?

☐ No
☐ Si: diferir el alta.

¿Está el recién nacido alimentándose con lactancia materna y correctamente?

☐ No: Capacitar y ayudar
☐ Si

Señalar la causa:
☐ VIH
☐ Hepatitis B
☐ CMV
☐ Otra________________________________

Confirmar que la mujer y/o el acompañante han sido informados de los signos de alarma ante los que pedir ayuda:

☐ No: Capacitar y ayudar
☐ Si

Señalar cuales conocen:
☐ Fiebre
☐ Respiración rápida o dificultad para respirar
☐ Extremadamente frío
☐ No orina o evacua
☐ Cianosis
☐ Interrupción de la correcta alimentación
☐ Crisis convulsivas
☐ Menos actividad de lo normal
☐ Ictericia
☐ Rechazo a vía oral o vómitos
☐ Cordón enrojecido, supurativo o con mal olor

¿Se le ha realizado al recién nacido laboratorio para grupo y Rh?

☐ No
☐ Si

Completado por: Nombre ________________________________

Cargo/Puesto ________________________________

Observaciones:

____
____
____
____
____
____
____
____
____
____
____
____

GOBIERNO DEL
ESTADO DE MÉXICO

GENTE QUE TRABAJA Y LOGRA
enGRANDE

ISEM

Hospital General de Toluca Dr. Nicolás San Juan
Hospital General de Ecatepec Dr. José María Rodríguez

HIDALGO
TIERRA DE TRABAJO

SECRETARÍA DE
SALUD

Estado Libre y Soberano
de Hidalgo

Hospital Obstétrico de Pachuca
Hospital General de Tula

INPer
INSTITUTO NACIONAL
DE PERINATOLOGÍA
ISIDRO ESPINOSA DE LOS REYES

Instituto Nacional
de Salud Pública

CONACYT
Consejo Nacional de Ciencia y Tecnología
