# Supplementary material for: Construction and pilot test of a set of indicators to assess the implementation and effectiveness of the who safe childbirth checklist
Source: BMC Pregnancy Childbirth. 2018 May 10;18:154. doi: 10.1186/s12884-018-1797-y (PMC5946578; doi:10.1186/s12884-018-1797-y)
Supplement: Supplementary file 2 — Mexico Safe Childbirth Checklist for mother and newborn (english). The original WHO Safe Childbirth Checklist was refined and adapted to the Mexican context and translated to english language. (PDF 458 kb) [file 12884_2018_1797_MOESM2_ESM.pdf]

World Health Organization

SAFE CHILDBIRTH CHECKLIST

MOTHER

Clinical record number	
Name	
Age	
Locality	
Education	
Languages	
Number of pregnancies	
Number of children	

NEWBORN

Name	
Date of birth	
Gestational age at birth	
Weight	
Lenth	
Apgar 1	Apgar 5

HOSPITAL

Name	
Locality	
Responsible for the service	
Responsible for the SCC	

STAGE IN WHICH THE MOTHER ARRIVES (mark an option)

- ☐ On admission
- ☐ Just before pushing (or caesarean)
- ☐ Soon after birth (within 1 hour)
- ☐ Before discharge

MOTIVO DE EGRESO

**Mother**	☐ Referred	☐ Discharge	☐ Death	**Newborn**	☐ Referred	☐ Discharge	☐ Death

1. ON ADMISSION

Did the mother have antenatal control?
- ☐ No, rate the risk
- ☐ Yes

Check:
Updated examinations of: group and Rh, blood count, HIV, VDRL / RPR, urine, biochemistry and ultrasound.

Does the mother need referral?
- ☐ No
- ☐ Yes

Indicate the cause and enable the protocol:
- ☐ Maternal
- ☐ Fetal
- ☐ Obstetric

Has the partogram been opened in the hospital?
- ☐ No
- ☐ Yes

Plot, monitor and register:
- ☐ Every 30 min: plot HR, contractions, fetal HR
- ☐ Every 2 hrs: plot temperature
- ☐ Every 4 hrs: plot BP
- ☐ Vaginal examination anddilatation according to evaluation and medical indication

---

Does the mother need to start treatment with ...?

Antibiotics
- ☐ No
- ☐ Yes, administered

Indicate the cause:
- ☐ Mother's temperature ≥38°C
- ☐ History of foul-smelling vaginal discharge
- ☐ Rupture of membranes >18 hrs
- ☐ Labor >24 hrs
- ☐ Others ____

Antihypertensive treatment?
- ☐ No
- ☐ Yes, antihypertensive medication given

Name of the medication _________________________________

Magnesium sulfate?
- ☐ No
- ☐ Yes, magnesium sulfate given

Give magnesium sulfate to mother if any of:
- ☐ Diastolic BP ≥110 mmHg and 3+ proteinuria
- ☐ Diastolic BP ≥90 mmHg, 2+ proteinuria,
- ☐ and any: severe headache, visual disturbance
- ☐ Epigastric pain (rule out liver hematoma)
- ☐ Exception: Renal insufficiency, myocardial injury, heart block, myasthenia gravis

---

Does the mother need to start anti-retroviral treatment?
- ☐ No
- ☐ Yes, administered

Woman with HIV + and in labor.
Name of the medication__________________________

Confirm supplies are available to clean hands and wear gloves for each vaginal exam:
- ☐ No
- ☐ Yes

Offered and Encourage birth companion to be present at birth:
- ☐ No
- ☐ Yes

Confirm that mother and/or companion have been informed of the warning signs and they will call for help if needed
- ☐ No
- ☐ Yes

Call for help if any of:
- ☐ Bleeding
- ☐ Severe abdominal pain
- ☐ Severe headache or visual disturbance
- ☐ Unable to urinate
- ☐ Urge to push

Is liquid intake allowed during labor?
- ☐ No
- ☐ Yes

Is free position allowed for delivery?
- ☐ No
- ☐ Yes

COMPLETED BY:	
Name	
Position:	

2. Just before pushing (or caesarean) - MOTHER

Does the mother needs a cesarean section?
- ☐ No
- ☐ Yes

A second opinion is made and indicate the cause:
- ☐ 2 previous caesarean sections
- ☐ transverse situation
- ☐ twin pregnancy
- ☐ pelvic presentation
- ☐ heart disease class III and IV
- ☐ fetal hydrocephalus
- ☐ history of uterine surgery
- ☐ macrosomia
- ☐ unstable fetal status
- ☐ HIV
- ☐ fetal malformations active genital herpes
- ☐ tumor that obstructs the birth canal
- ☐ premature detachment of placenta normoinserta
- ☐ Death product greater than 30 SDG in a patient without labor for more than 24 hours.
- ☐ total placenta previa
- ☐ abdominal via cerclage

Does the mother have an indication of instrumental delivery?
- ☐ No
- ☐ Yes

Indicate the cause:
- ☐ previous cesarean section
- ☐ expulsive with fetal instability
- ☐ maternal heart disease
- ☐ maternal exhaustion
- ☐ Other ____________________________________

Does the mother have an indication for an episiotomy?
- ☐ No
- ☐ Yes

Cause ____

Does the mother need to start treatment with ...?

Antibiotics
- ☐ No
- ☐ Yes, administered

Indicate the cause:
- ☐ Mother's temperature ≥38°C
- ☐ History of foul-smelling vaginal discharge
- ☐ Rupture of membranes >18 hrs
- ☐ Labor >24 hrs
- ☐ Cesarean section start
- ☐ Others ___________________________

Antihypertensive treatment?
- ☐ No
- ☐ Yes, given

Name of the medication _________________________

Magnesium sulfate?
- ☐ No
- ☐ Yes, given

Give magnesium sulfate to mother if any of:
- ☐ Diastolic BP ≥110 mmHg and 3+ proteinuria
- ☐ Diastolic BP ≥90 mmHg, 2+ proteinuria,
- ☐ and any: severe headache, visual disturbance
- ☐ Epigastric pain (rule out liver hematoma)
- ☐ Exception: Renal insufficiency, myocardial injury, heart block, myasthenia gravis
- ☐ Others____________________________

Confirm essential supplies for mother are at bedside and prepare for delivery:
- ☐ No
- ☐ Yes

- ☐ Gloves
- ☐ Alcohol-based handrub or soap and clean water
- ☐ Oxytocin 10 units in syringe
- ☐ 2 ring forceps

Assistant identified, informed and ready to help at birth if needed
- ☐ No
- ☐ Yes

Name________________________________

COMPLETED BY:

Name	
Position:	

2. Just before pushing (or caesarean) - NEWBORN

Confirm essential supplies for newborn are available and prepare:

- ☐ No
- ☐ Yes

- ☐ Clean towel
- ☐ Tie or cord clamp
- ☐ Sterile blade to cut cord
- ☐ O2 source and suction device
- ☐ Bag-and-mask
- ☐ Sewer equipment
- ☐ Laryngoscope
- ☐ Adrenalin
- ☐ Endotracheal intubation cannulas
- ☐ Saline and glucose solution
- ☐ Vitamin K
- ☐ Ophthalmic prophylaxis

Are there trained staff in neonatal resuscitation?

- ☐ No
- ☐ Yes

Name_____________________________________

COMPLETED BY:

Name	
Position:	

3. Soon after birth (within 1 hour) - MOTHER

Care for mother immediately after birth

- ☐ No
- ☐ Yes

- ☐ Confirm single baby only (not multiple birth)
- ☐ Give oxytocin within 1 minute after birth
- ☐ Cord traction control for deliver placenta
- ☐ Massage uterus after placenta is delivered

Is mother bleeding abnormally?

- ☐ No
- ☐ Yes, shout for help

Actions in postpartum if there is bleeding ≥ 500 mL, or if ≥ 250 mL and severe anemia:

- ☐ Massage uterus
- ☐ Consider more uterotonic
- ☐ Start IV fluids
- ☐ Activate rapid response team for obstetric emergencies
- ☐ Treat cause

Does the mother need to start treatment with ...?

Antibiotics

- ☐ No
- ☐ Yes

Cause ____

Antihypertensive treatment?

- ☐ No
- ☐ Yes

Name of the medication ____

Magnesium sulfate?

- ☐ No
- ☐ Yes, given

Give magnesium sulfate to mother if any of:

- ☐ Diastolic BP ≥110 mmHg and 3+ proteinuria
- ☐ Diastolic BP ≥90 mmHg, 2+ proteinuria,
- ☐ and any: severe headache, visual disturbance
- ☐ Epigastric pain (rule out liver hematoma)
- ☐ Exception: renal insufficiency, myocardial injury, heart block, myasthenia gravis
- ☐ Others____

Confirm that mother and/or companion have been informed of the warning signs and they will call for help if needed

- ☐ No
- ☐ Yes

Which of these do they know:

- ☐ Bleeding
- ☐ Strong abdominal pain
- ☐ Strong headache
- ☐ Visual alterations
- ☐ Respiratory distress
- ☐ Difficulty emptying the bladder
- ☐ Fever and / or chills

COMPLETED BY:

Name	
Position:	

3. Soon after birth (within 1 hour) - NEWBORN

Newborn care:

Dry the newborn	Keep warm	Vitamin K	Ophthalmic prophylaxis
☐ No	☐ No	☐ No	☐ No
☐ Yes	☐ Yes	☐ Yes	☐ Yes

Late clamping of umbilical cord
☐ No
☐ Yes
Cause ____

Skin-to-skin contact started immediately
☐ No
☐ Yes
Cause ____

Breastfeeding started immediately
☐ No
☐ Yes
Cause ____

Does baby need…

Special care and monitoring?
☐ No
☐ Yes, organized

Indicate cause:
☐ More than 1 month early
☐ Birth weight <2500 grams
☐ Needs antibiotics
☐ Required resuscitation
☐ Hypoglycemia
☐ Respiratory distress

Referral?
☐ No
☐ Yes, organized

Indicate cause:
☐ Suspicion of sepsis
☐ Respiratory distress
☐ Suspected neonatal pathology (which): ______________________

Antibiotics?
☐ No
☐ Yes, given

Indicate cause:
☐ Antibiotics given to mother
☐ Respiratory rate >60/min or <30/min
☐ Chest in-drawing, grunting, or convulsions
☐ Rupture of membranes >18 hrs
☐ chorioamnionitis
☐ Poor movement on stimulation
☐ Baby's temperature <35 °C (and not rising after warming)or baby's temperature ≥38 °C
☐ Other ________________

Antiretroviral treatment?
☐ No
☐ Yes, given

Administered in the first 6 hours if the mother has HIV +.
Name of the medication:____

Confirm that mother and/or companion have been informed of the warning signs and they will call for help if needed
☐ No
☐ Yes

Which of these do they know:
☐ Fast/difficult breathing
☐ Fever
☐ Unusually cold
☐ Stops feeding well
☐ Less activity than normal
☐ Seizures
☐ Whole body becomes yellow

COMPLETED BY:

Name	
Position:	

4. Before discharge - MOTHER

Is the bleeding controlled?

- ☐ No, treat and delay discharge
- ☐ Yes

Does the mother need to start treatment with ...?

Antibiotics

- ☐ No
- ☐ Yes

Indicate cause:

- ☐ Temperature ≥38 °C and: shaking chills, foul-smelling vaginal discharge or undertone or abdominal distention
- ☐ Others: ____

Has the mother been offered information about family planning before discharge?

- ☐ No: give guidance and counseling
- ☐ Yes

Confirm that mother and/or companion have been informed of the warning signs and they will call for help if needed

- ☐ No
- ☐ Yes

Which of these do they know:

- ☐ Bleeding
- ☐ Strong abdominal pain
- ☐ Strong headache
- ☐ Visual alterations
- ☐ Respiratory distress
- ☐ Difficulty emptying the bladder
- ☐ Altered state of consciousness
- ☐ Fever and / or chills

If the mother is HIV +: Do the mother and the newborn have anti-retrovirals for the complete treatment (1 month for the newborn and the mother continued with her follow-up)?

- ☐ No
- ☐ Yes

Has the follow-up of mother and newborn been agreed and organized?

- ☐ No
- ☐ Yes

COMPLETED BY:	
Name	
Position:	

4. Before discharge - NEWBORN

Does baby need…

Antibiotics?

- ☐ No
- ☐ Yes, treat and delay discharge and gives specials care or refer if is neded

Indicate cause:

- ☐ Antibiotics given to mother
- ☐ Respiratory rate >60/min or <30/min
- ☐ Chest in-drawing, grunting, or convulsions
- ☐ Rupture of membranes >18 hrs
- ☐ Chorioamnionitis
- ☐ Poor movement on stimulation
- ☐ Baby's temperature <35 °C (and not rising after warming)or baby's temperature ≥38 °C
- ☐ Other ____________________

Does the newborn have jaundice?

- ☐ No
- ☐ Yes: delay discharge

Is baby feeding well?

- ☐ No, train and help
- ☐ Yes

Indicate cause:

- ☐ Mother with HIV+
- ☐ Hepatitis B
- ☐ Cytomegalovirus
- ☐ Other____

Confirm that mother and/or companion have been informed of the warning signs and they will call for help if needed

- ☐ No
- ☐ Yes

Which of these do they know:

- ☐ Fast/difficult breathing
- ☐ Fever
- ☐ Unusually cold
- ☐ Stops feeding well
- ☐ Less activity than normal
- ☐ Seizures
- ☐ Whole body becomes yellow
- ☐ Do not urinate or evacuate
- ☐ Cyanosis
- ☐ Vomiting / rejection intake
- ☐ Umbilical cord: flushed, with suppuration or bad smell

Has newborn been tested for blood group and Rh been?

- ☐ No
- ☐ Yes

COMPLETED BY:

Name	
Position:	
