# Supplementary material for: Construction and pilot test of a set of indicators to assess the implementation and effectiveness of the who safe childbirth checklist
Source: BMC Pregnancy Childbirth. 2018 May 10;18:154. doi: 10.1186/s12884-018-1797-y (PMC5946578; doi:10.1186/s12884-018-1797-y)
Supplement: Supplementary file 3 — Set of SCC indicators (spanish version). The detailed definitions of the indicators in Spanish. (DOCX 116 kb) [file 12884_2018_1797_MOESM3_ESM.docx]

**FICHAS DE INDICADORES PROPUESTOS PARA LA LISTA DE VERIFICACIÓN DE NACIMIENTO SEGURO (LVNS)**

INDICE

[1. Indicadores para implementación 2](#_Toc510078572)

[1.1. Capacitación 2](#_Toc510078573)

[1.2. Equipo 4](#_Toc510078574)

[1.3. Actitud 9](#_Toc510078575)

[1.4. Disponibilidad 12](#_Toc510078576)

[2. Indicadores utilización 13](#_Toc510078577)

[3. Indicadores buenas prácticas 17](#_Toc510078578)

[3.1. Generales 17](#_Toc510078579)

[3.2. Indicadores para fase de admisión 24](#_Toc510078580)

[3.3. Indicadores para fase pre-expulsiva (o antes de cesárea) 28](#_Toc510078581)

[3.4. Indicadores para fase Post-parto inmediato (primera hora tras la expulsión) 33](#_Toc510078582)

[3.5. Indicadores para fase justo antes del alta 40](#_Toc510078583)

[4. Indicadores de resultado 44](#_Toc510078584)

# Indicadores para implementación

| FICHA TÉCNICA DE INDICADORES: estructura | |
| --- | --- |
| **GRUPO:** | Factores que influyen en la implementación de la LVNS |
| **AREA:** | Personal de salud |
| Sub-área | Capacitación |
| **Nº** | 1 (de 2) |
| **NOMBRE INDICADOR** | Profesionales capacitados en el uso de la LVNS |
| **FORMA DE MEDICIÓN** | Encuesta a profesionales y registros de capacitación |
| **DESCRIPCIÓN** | Porcentaje de profesionales que recibieron capacitación en el uso de la LVNS |
| **Nivel de evidencia; Fuerza recomendación** | No aplica |
| **FÓRMULA DEL INDICADOR** | Número de profesionales que responden que han recibido capacitación en el uso de la lista de verificación de parto seguro |
| **Numerador** |  |
| **Denominador** | Total de profesionales de los servicios de urgencias, cirugía y obstetricia involucrados en el proceso de atención al parto. |
| **FUENTE DE DATOS** | Cuestionario  Listados de capacitación |
| **ELABORACIÓN DEL INDICADOR** | Propio |
| **REFERENCIAS BIBLIOGRÁFICAS**  No aplica | |
| **OBSERVACIONES**  Se sugiere obtener el porcentaje total para el centro, y el desagregado por servicio y por turno. | |

## 1.1. Capacitación

| FICHA TÉCNICA DE INDICADORES: estructura | |
| --- | --- |
| **GRUPO:** | Factores que influyen en la implementación de la LVNS |
| **AREA:** | Personal de salud |
| Sub-área | Capacitación |
| **Nº** | 2 (de 2) |
| **NOMBRE INDICADOR** | Profesionales capacitados en el uso de la LVNS por perfil profesional |
| **FORMA DE MEDICIÓN** | Encuesta a profesionales y registros de capacitación |
| **DESCRIPCIÓN** | Porcentaje de profesionales que recibieron capacitación en el uso de la LVNS según perfil profesional |
| **Nivel de evidencia; Fuerza recomendación** | No aplica |
| **FÓRMULA DEL INDICADOR** | Número de profesionales por perfil que responden que han recibido capacitación en el uso de la lista de verificación de parto seguro |
| **Numerador** |  |
| **Denominador** | Total de profesionales por perfil |
| **FUENTE DE DATOS** | Cuestionario  Listados de capacitación |
| **ELABORACIÓN DEL INDICADOR** | Propio |
| **REFERENCIAS BIBLIOGRÁFICAS**  No aplica | |
| **OBSERVACIONES**  Desagregar resultados por los siguientes perfiles profesionales:   - Enfermera/o general - Enfermero/a obstétrica - Doula - Psicóloga/o - Médico general - Médico ginecólogo/obstetra - Médico neonatólogo/pediatra - Médico residente | |

## 1.2. Equipo

| FICHA TÉCNICA DE INDICADORES: estructura | |
| --- | --- |
| **GRUPO:** | Factores que influyen en la implementación de la LVNS |
| **AREA:** | Personal de salud |
| Sub-área | Equipo |
| **Nº** | 1 (de 5) |
| **NOMBRE INDICADOR** | Tamaño del equipo (variable de análisis) |
| **FORMA DE MEDICIÓN** | Auditoría |
| **DESCRIPCIÓN** | Razón de profesionales en relación a las camas de los servicios de ginecología-obstetricia y neonatología |
| **Nivel de evidencia; Fuerza recomendación** | No identificado |
| **FÓRMULA DEL INDICADOR** | N° de profesionales del equipo |
| **Numerador** |  |
| **Denominador** | Camas del servicio de ginecología-obstetricia y neonatología |
| **FUENTE DE DATOS** | Plantilla del personal; Datos administrativos del hospital  Cuestionario a profesionales |
| **ELABORACIÓN DEL INDICADOR** | Propio |
| **REFERENCIAS BIBLIOGRÁFICAS**  No identificado | |
| **OBSERVACIONES**  Desagregar por perfil profesional, por servicio y por turno, siempre en base al número de camas disponibles para la atención al parto en el servicio de ginecología-obstetricia y de neonatología. Opcionalmente, se puede calcular también la razón por número de camas del centro asistencial.  Turnos: mañana, tarde, noche, fin de semana y festivo  Perfiles del equipo:   - Enfermera/o general - Enfermero/a obstétrica - Doula - Psicóloga/o - Médico general - Médico ginecólogo/obstetra - Médico neonatólogo/pediatra - Médico residente | |

| FICHA TÉCNICA DE INDICADORES: estructura | |
| --- | --- |
| **GRUPO:** | Factores que influyen en la implementación de la LVNS |
| **AREA:** | Personal de salud |
| Sub-área | Equipo |
| **Nº** | 2 (de 5) |
| **NOMBRE INDICADOR** | Perfil profesional de integrantes del equipo (variable de análisis) |
| **FORMA DE MEDICIÓN** | Auditoría |
| **DESCRIPCIÓN** | Volumen de cada perfil profesional del equipo de trabajo (servicio y turno) |
| **Nivel de evidencia; Fuerza recomendación** | No identificado |
| **FÓRMULA DEL INDICADOR** | N° de profesionales de cada perfil profesional por servicio y turno |
| **Numerador** |  |
| **Denominador** | No aplica |
| **FUENTE DE DATOS** | Plantilla del personal del centro asistencial  Cuestionario a profesionales |
| **ELABORACIÓN DEL INDICADOR** | Propio |
| **REFERENCIAS BIBLIOGRÁFICAS**  No identificado | |
| **OBSERVACIONES**  Servicios: urgencias, cirugía y ginecología-obstetricia  Turnos: mañana, tarde, noche, fin de semana y festivo  Perfiles del equipo:   - Enfermera/o general - Enfermero/a obstétrica - Doula - Psicóloga/o - Médico general - Médico ginecólogo/obstetra - Médico neonatólogo/pediatra - Médico residente | |

| FICHA TÉCNICA DE INDICADORES: estructura | |
| --- | --- |
| **GRUPO:** | Factores que influyen en la implementación de la LVNS |
| **AREA:** | Personal de salud |
| Sub-área | Equipo |
| **Nº** | 3 (de 5) |
| **NOMBRE INDICADOR** | Disponibilidad de personal capacitado en reanimación neonatal en el servicio |
| **FORMA DE MEDICIÓN** | Auditoria; Encuesta |
| **DESCRIPCIÓN** | Existencia de personal con capacitación actualizada en reanimación neonatal en cada servicio y cada turno |
| **Nivel de evidencia; Fuerza recomendación** | No aplica |
| **FÓRMULA DEL INDICADOR** | Existencia de profesionales con capacitación actualizada en reanimación neonatal en cada servicio y cada turno |
| **Numerador** |  |
| **Denominador** | No aplica |
| **FUENTE DE DATOS** | Cuestionario a profesionales |
| **ELABORACIÓN DEL INDICADOR** | Propio |
| **REFERENCIAS BIBLIOGRÁFICAS**  No aplica | |
| **OBSERVACIONES**  Se entiende por capacitación actualizada que esté vigente (realizada en un periodo menor a 2 años).  Servicios: urgencias, cirugía y ginecología-obstetricia  Turnos: mañana, tarde, noche, fin de semana y festivo | |

| FICHA TÉCNICA DE INDICADORES: estructura | |
| --- | --- |
| **GRUPO:** | Factores que influyen en la implementación de la LVNS |
| **AREA:** | Personal de salud |
| Sub-área | Equipo |
| **Nº** | 4 (de 5) |
| **NOMBRE INDICADOR** | Composición del equipo que atiende el parto (etapa pre-expulsiva) |
| **FORMA DE MEDICIÓN** | Observación |
| **DESCRIPCIÓN** | Perfil del equipo que atendió cada parto |
| **Nivel de evidencia; Fuerza recomendación** | No aplica |
| **FÓRMULA DEL INDICADOR** | Tipología de composición del equipo presentes en el momento del parto (para cada parto) |
| **Numerador** |  |
| **Denominador** | No aplica |
| **FUENTE DE DATOS** | Observación directa  Libros de registro  Cuestionario a profesionales |
| **ELABORACIÓN DEL INDICADOR** | Propio |
| **REFERENCIAS BIBLIOGRÁFICAS**  No aplica | |
| **OBSERVACIONES**  Se debe registrar los perfiles profesionales existentes para cada parto así como la existencia del profesional con formación en reanimación neonatal (ver más abajo).  Se reporta independientemente el número de profesionales presente en el parto por cada perfil. Se valorará independientemente cada turno.  Turnos: mañana, tarde, noche, fin de semana y festivo  Perfiles del equipo:   1. Enfermera/o general 2. Enfermero/a obstétrica 3. Doula 4. Psicóloga/o 5. Médico general 6. Médico ginecólogo/obstetra 7. Médico residente 8. Médico neonatólogo/pediatra 9. Cualquier categoría de la “*a*” a la “*g*” con formación en reanimación neonatal   NOTA: Para la identificación de profesionales con formación, se deberá haber identificado previamente quienes tienen esta capacitación. En el caso del médico neonatólogo, se considerará directamente dicha capacitación. | |

| FICHA TÉCNICA DE INDICADORES: estructura | |
| --- | --- |
| **GRUPO:** | Factores que influyen en la implementación de la LVNS |
| **AREA:** | Personal de salud |
| Sub-área | Equipo |
| **Nº** | 5 (de 5) |
| **NOMBRE INDICADOR** | Presencia de personal capacitado en reanimación neonatal en el parto |
| **FORMA DE MEDICIÓN** | Observación; revisión de expedientes clínicos |
| **DESCRIPCIÓN** | Porcentaje de partos con presencia de algún profesional con capacitación actualizada en reanimación neonatal |
| **Nivel de evidencia; Fuerza recomendación** | Recomendación de expertos |
| **FÓRMULA DEL INDICADOR** | Presencia de profesionales con capacitación actualizada en reanimación neonatal en el momento del parto |
| **Numerador** |  |
| **Denominador** | Número de partos atendidos |
| **FUENTE DE DATOS** | Observación directa  Libros de registro  Cuestionario a profesionales  LVNS |
| **ELABORACIÓN DEL INDICADOR** | Propio |
| **REFERENCIAS BIBLIOGRÁFICAS**  ^1^ Asociación Española de Pediatría y Sociedad Española de Neonatología. Protocolos Diagnóstico Terapéuticos de la AEP: Neonatología. 2ª edición. 2008. p. 111-125. <http://www.aeped.es/documentos/protocolos-neonatologia>  ^2^ OMS-Departamento de Investigación y Salud Reproductiva. Cuidados en el parto normal: una guía práctica. Informe presentado por el Grupo Técnico de Trabajo. Ginebra. 1996. | |
| **OBSERVACIONES**  *^1^En todo parto debe haber al menos una persona responsabilizada de la atención al recién nacido con capacitación en reanimación neonatal inicial.*  *^2^La persona que asiste el parto debe ser capaz de llevar a cabo intervenciones básicas esenciales y de cuidar al niño después del nacimiento.*  Se entiende por capacitación actualizada en reanimación neonatal: curso “homologado/certificado” realizado o actualizado en un periodo menor a 2 años.  NOTA: Para la evaluación de este indicador, se deberá haber identificado previamente quienes tienen esta capacitación. En el caso del médico neonatólogo, se considerará directamente dicha capacitación. | |

## 1.3. Actitud

| FICHA TÉCNICA DE INDICADORES: estructura | |
| --- | --- |
| **GRUPO:** | Factores que influyen en la implementación de la LVNS |
| **AREA:** | Personal de salud |
| Sub-área | Actitud |
| **Nº** | 1 (de 3) |
| **NOMBRE INDICADOR** | Percepción de utilidad de la lista de verificación de parto seguro |
| **FORMA DE MEDICIÓN** | Encuesta |
| **DESCRIPCIÓN** | Porcentaje de profesionales que opinan que el uso de la LVNS no es una pérdida de tiempo |
| **Nivel de evidencia; Fuerza recomendación** | No aplica |
| **FÓRMULA DEL INDICADOR** | Número de profesionales que opinan que el uso de la LVNS “nunca” es una pérdida de tiempo |
| **Numerador** |  |
| **Denominador** | Número de profesionales que respondieron al cuestionario de cada servicio |
| **FUENTE DE DATOS** | Cuestionario *ad hoc* a profesionales |
| **ELABORACIÓN DEL INDICADOR** | Propio |
| **REFERENCIAS BIBLIOGRÁFICAS**   - Saturno, P. J., Soria-Aledo, V., Da Silva Gama, Z. a., Lorca-Parra, F., & Grau-Polan, M. (2014). Understanding WHO surgical checklist implementation: Tricks and pitfalls. An observational study. *World Journal of Surgery*, *38*(2), 287–295. doi:10.1007/s00268-013-2300-6 | |
| **OBSERVACIONES**  Se valorará de forma independiente cada perfil profesional, por turnos.  Posibles respuestas de la encuesta a la pregunta: *En su opinión, ¿la lista de verificación es una pérdida de tiempo en el proceso del parto?*  Categorías:  1. N° respuestas “siempre”  2. N° respuestas “casi siempre”  3. N° respuestas “a veces”  4. N° respuestas “casi nunca”  5. N° respuestas “nunca”  Se obtendrá como resultado de este indicador la proporción de respuestas a la rúbrica “nunca”. | |

| FICHA TÉCNICA DE INDICADORES: estructura | |
| --- | --- |
| **GRUPO:** | Factores que influyen en la implementación de la LVNS |
| **AREA:** | Personal de salud |
| Sub-área | Actitud |
| **Nº** | 2 (de 3) |
| **NOMBRE INDICADOR** | Percepción del impacto de la lista de verificación de parto seguro |
| **FORMA DE MEDICIÓN** | Encuesta |
| **DESCRIPCIÓN** | Porcentaje de profesionales que opinan que el uso de la LVNS mejora la seguridad de la atención al parto |
| **Nivel de evidencia; Fuerza recomendación** | No aplica |
| **FÓRMULA DEL INDICADOR** | Número de profesionales que opinan que el uso de la LVNS “siempre” mejora la seguridad en el parto |
| **Numerador** |  |
| **Denominador** | Número de profesionales que respondieron al cuestionario de cada servicio |
| **FUENTE DE DATOS** | Cuestionario *ad hoc* a profesionales |
| **ELABORACIÓN DEL INDICADOR** | Propio |
| **REFERENCIAS BIBLIOGRÁFICAS**   - Saturno, P. J., Soria-Aledo, V., Da Silva Gama, Z. a., Lorca-Parra, F., & Grau-Polan, M. (2014). Understanding WHO surgical checklist implementation: Tricks and pitfalls. An observational study. *World Journal of Surgery*, *38*(2), 287–295. doi:10.1007/s00268-013-2300-6 | |
| **OBSERVACIONES**  Se valorará de forma independiente cada perfil profesional por turnos.  Posibles respuestas de la encuesta a la pregunta *“En su opinión, ¿la lista de verificación* *mejora la seguridad de la atención al parto?”*:  Categorías:  1. N° respuestas “siempre”  2. N° respuestas “casi siempre”  3. N° respuestas “a veces”  4. N° respuestas “casi nunca”  5. N° respuestas “nunca”  Se obtendrá como resultado de este indicador la proporción de respuestas a la rúbrica “siempre”. | |

| FICHA TÉCNICA DE INDICADORES: estructura | |
| --- | --- |
| **GRUPO:** | Factores que influyen en la implementación de la LVNS |
| **AREA:** | Personal de salud |
| Sub-área | Actitud |
| **Nº** | 3 (de 3) |
| **NOMBRE INDICADOR** | Percepción de relevancia para la institución de la implantación de la lista de verificación de parto seguro |
| **FORMA DE MEDICIÓN** | Encuesta |
| **DESCRIPCIÓN** | Porcentaje de profesionales que perciben relevante para su institución la implementación de la lista de verificación de parto seguro |
| **Nivel de evidencia; Fuerza recomendación** | No aplica |
| **FÓRMULA DEL INDICADOR** | Número de profesionales que perciben relevante para su institución la implementación de la lista de verificación de parto seguro |
| **Numerador** |  |
| **Denominador** | Número de profesionales que respondieron al cuestionario de cada servicio |
| **FUENTE DE DATOS** | Cuestionario *ad hoc* a profesionales |
| **ELABORACIÓN DEL INDICADOR** | Propio |
| **REFERENCIAS BIBLIOGRÁFICAS**  No aplica | |
| **OBSERVACIONES**  Se valorará de forma independiente cada categoría profesional por turnos.  Posibles respuestas de la encuesta a la pregunta *“¿Cree que su unidad hospitalaria considera importante la implementación de la lista de verificación de parto seguro?”*   - Sí - No - No sabe/No contesta   Sólo se considerarán las respuestas cuya rúbrica sea “Sí”. | |

## 1.4. Disponibilidad

| FICHA TÉCNICA DE INDICADORES: estructura | |
| --- | --- |
| **GRUPO:** | Factores que influyen en la implementación de la LVNS |
| **AREA:** | Infraestructura |
| Sub-área | Disponibilidad de LVNS |
| **Nº** | 1 (de 1) |
| **NOMBRE INDICADOR** | Disponibilidad de formato de lista de verificación de parto seguro cuando se necesita |
| **FORMA DE MEDICIÓN** | Encuesta a profesionales |
| **DESCRIPCIÓN** | Porcentaje de profesionales que encuentran disponible el formato de lista de verificación de parto seguro cuando lo necesitan |
| **Nivel de evidencia; Fuerza recomendación** | No aplica |
| **FÓRMULA DEL INDICADOR** | Número de profesionales por servicio que responden que “siempre” encuentran disponible el formato de lista de verificación de parto seguro cuando lo necesitan |
| **Numerador** |  |
| **Denominador** | Total de profesionales que respondieron el cuestionario por cada servicio y turno |
| **FUENTE DE DATOS** | Cuestionario |
| **ELABORACIÓN DEL INDICADOR** | Propio |
| **REFERENCIAS BIBLIOGRÁFICAS**  No aplica | |
| **OBSERVACIONES**  Se obtendrán proporciones por cada una de las respuestas de frecuencia a la pregunta “*¿En su experiencia, con qué frecuencia encuentra disponible el formato de lista de verificación de parto seguro cuando lo necesita?”*:   1. N° respuestas “siempre”/total de profesionales del servicio. 2. N° respuestas “casi siempre”/total de profesionales del servicio. 3. N° respuestas “a veces”/total de profesionales del servicio. 4. N° respuestas “casi nunca”/total de profesionales del servicio. 5. N° respuestas “nunca”/total de profesionales del servicio.   Se obtendrá como resultado de este indicador la proporción de respuestas a la rúbrica “siempre”.  Se desagrega por servicio y turno encuestado. | |

# Indicadores utilización

| FICHA TÉCNICA DE INDICADORES: proceso | |
| --- | --- |
| **GRUPO:** | Utilización de la LVNS |
| **AREA:** |  |
| Sub-área |  |
| **Nº** | 1 (de 4) |
| **NOMBRE INDICADOR** | Utilización del formato de LVNS en el hospital (presencia en el expediente) |
| **FORMA DE MEDICIÓN** | Muestreo de expedientes de mujeres que fueron atendidas por motivo de parto: Revisión de llenado de las LVNS |
| **DESCRIPCIÓN** | Porcentaje de expedientes clínicos con LVNS |
| **Nivel de evidencia; Fuerza recomendación** | No aplica |
| **FÓRMULA DEL INDICADOR** | Número de expedientes clínicos con lista de verificación de parto seguro utilizados |
| **Numerador** |  |
| **Denominador** | Número total de expedientes de partos atendidos |
| **FUENTE DE DATOS** | Expedientes clínicos |
| **ELABORACIÓN DEL INDICADOR** | Propio |
| **REFERENCIAS BIBLIOGRÁFICAS**  No aplica | |
| **OBSERVACIONES**  Ejemplo para frecuencia de recogida de datos cada 15 días:  En 15 días ha habido 130 partos  En los mismos 15 días se han utilizado (iniciado) LVNS en 90 partos  90/130 = 0.69  La LVNS se ha utilizado en el 69% de los partos que se han atendido en los últimos 15 días (el porcentaje de utilización de la LVNS ha sido del 69%)  Este indicador permite contrastar la información con el cuestionario a profesionales: “*En los partos que usted asiste, ¿con qué frecuencia utiliza el formato de lista de verificación de parto seguro?*” | |

| FICHA TÉCNICA DE INDICADORES: proceso | |
| --- | --- |
| **GRUPO:** | Utilización de la LVNS |
| **AREA:** |  |
| Sub-área |  |
| **Nº** | 2 (de 4) |
| **NOMBRE INDICADOR** | Utilización del formato de LVNS en el hospital (LVNS completa) |
| **FORMA DE MEDICIÓN** | Muestreo de expedientes de mujeres que fueron atendidas por motivo de parto: Revisión de llenado de las LVNS |
| **DESCRIPCIÓN** | Porcentaje de utilización de la LVNS completa |
| **Nivel de evidencia; Fuerza recomendación** | No aplica |
| **FÓRMULA DEL INDICADOR** | Número de formatos de lista de verificación de parto seguro completados totalmente |
| **Numerador** |  |
| **Denominador** | Número de formatos de lista de verificación de parto seguro utilizados |
| **FUENTE DE DATOS** | LVNS |
| **ELABORACIÓN DEL INDICADOR** | Propio |
| **REFERENCIAS BIBLIOGRÁFICAS**  No aplica | |
| **OBSERVACIONES**  Debe haber concordancia con el momento de entrada de la mujer al centro. Así, si una paciente entra en el hospital habiéndose saltado una etapa de la lista de verificación, esta etapa no se tendrá en cuenta para la atribución de cumplimiento/incumplimiento.  Sólo se tendrán en cuenta las fases en las que ha llegado la embarazada y las subsiguientes. | |

| FICHA TÉCNICA DE INDICADORES: proceso | |
| --- | --- |
| **GRUPO:** | Utilización de la LVNS |
| **AREA:** |  |
| Sub-área |  |
| **Nº** | 3 (de 4) |
| **NOMBRE INDICADOR** | Items completados de la LVNS en global |
| **FORMA DE MEDICIÓN** | Muestreo de expedientes de mujeres que fueron atendidas por motivo de parto: Revisión de llenado de las LVNS. |
| **DESCRIPCIÓN** | Media ponderada del porcentaje de ítems llenados de toda la LVNS en global |
| **Nivel de evidencia; Fuerza recomendación** | No aplica |
| **FÓRMULA DEL INDICADOR** | No aplica |
| **Numerador** |  |
| **Denominador** | No aplica |
| **FUENTE DE DATOS** | LVNS |
| **ELABORACIÓN DEL INDICADOR** | Propio |
| **REFERENCIAS BIBLIOGRÁFICAS**  No aplica | |
| **OBSERVACIONES**  Media ponderada realizada a partir del porcentaje global de cada LVNS utilizada en el centro, en función de las fases que hayan aplicado a cada caso.  Este indicador se calculará a partir del indicador “Items completados de la LVNS en cada etapa”. | |

| FICHA TÉCNICA DE INDICADORES: proceso | |
| --- | --- |
| **GRUPO:** | Utilización de la LVNS |
| **AREA:** |  |
| Sub-área |  |
| **Nº** | 4 (de 4) |
| **NOMBRE INDICADOR** | Items completados de la LVNS en cada etapa |
| **FORMA DE MEDICIÓN** | Muestreo de expedientes de mujeres que fueron atendidas por motivo de parto: Revisión de llenado de las LVNS. |
| **DESCRIPCIÓN** | Porcentaje de ítems llenados por cada una de las 4 etapas |
| **Nivel de evidencia; Fuerza recomendación** | No aplica |
| **FÓRMULA DEL INDICADOR** | Número de ítems de la lista de verificación de parto seguro llenados por cada etapa |
| **Numerador** |  |
| **Denominador** | Número de ítems por cada etapa (*) de todas las listas de verificación de parto seguro utilizadas |
| **FUENTE DE DATOS** | LVNS |
| **ELABORACIÓN DEL INDICADOR** | Propio |
| **REFERENCIAS BIBLIOGRÁFICAS**  No aplica | |
| **OBSERVACIONES**  (*) Se obtendrá un porcentaje por cada etapa.  Se ha de verificar en el expediente clínico la fase en la que llegó la parturienta al centro asistencial. Sólo se tendrán en cuenta la etapa en la que comenzó la asistencia y las subsiguientes. Cualquier etapa anterior a éstas, será excluida de la evaluación.  Se desagrega los resultados de este indicador por ítems de la LVNS para la madre y para el niño.  Número de ítems por fase:  Admisión: 12 madre  Antes de pujar: 10 (8 madre y 2 de recién nacido)  Post-parto inmediato: 18 (6 madre y 12 de recién nacido)  Antes de egreso: 11 (6 madre y 5 de recién nacido) | |

# Indicadores buenas prácticas

## 3.1. Generales

| FICHA TÉCNICA DE INDICADORES: PROCESO | |
| --- | --- |
| **GRUPO:** | Buenas prácticas |
| **AREA:** | Generales |
| Sub-área | Uso de antibióticos |
| **Nº** | 1 (de 4) |
| **NOMBRE INDICADOR** | Prescripción de antibióticos durante el parto |
| **FORMA DE MEDICIÓN** | Muestreo de expedientes de mujeres que fueron atendidas por motivo de parto |
| **DESCRIPCIÓN** | Porcentaje de mujeres a quienes se prescribe antibiótico durante el proceso del parto |
| **Nivel de evidencia; Fuerza recomendación** | No aplica |
| **FÓRMULA DEL INDICADOR** | Número de mujeres durante el proceso del parto a las que se prescribe antibiótico |
| **Numerador** |  |
| **Denominador** | Número de mujeres atendidas por motivo de parto |
| **FUENTE DE DATOS** | Expedientes clínicos  LVNS (si es caso) |
| **ELABORACIÓN DEL INDICADOR** | Propio |
| **REFERENCIAS BIBLIOGRÁFICAS**   - World Health Organization (WHO). WHO - Safe childbirth checklist [Internet]. Available from: <http://www.who.int/patientsafety/implementation/checklists/childbirth/en/> | |
| **OBSERVACIONES**  Prescripción de antibiótico:  Será válida cualquier indicación o prescripción de antibiótico en el expediente clínico durante todo el proceso del parto (desde la admisión hasta el egreso). Se capturará la fase del proceso asistencial (admisión, pre-expulsiva, post-parto inmediato o antes del egreso) en el que se realiza la indicación o prescripción, si bien el indicador se reporta para el proceso global de atención al parto.  Para fines de validación de la LVNS, se puede contrastar lo marcado en ésta independientemente de la causa que lo justifique, ya sea para el proceso global o por la fase de inicio (prescripción) de antibiótico. | |

| FICHA TÉCNICA DE INDICADORES: PROCESO | |
| --- | --- |
| **GRUPO:** | Buenas prácticas |
| **AREA:** | Generales |
| Sub-área | Uso de antibióticos |
| **Nº** | 2 (de 4) |
| **NOMBRE INDICADOR** | Manejo de antibióticos durante el parto |
| **FORMA DE MEDICIÓN** | Muestreo de expedientes de mujeres que fueron atendidas por motivo de parto y se les prescribió antibiótico |
| **DESCRIPCIÓN** | Porcentaje de mujeres a las que se prescribe antibiótico en alguna fase del parto y está registrado algún síntoma que lo justifique |
| **Nivel de evidencia; Fuerza recomendación** | II-2 - Kaimal A, 2008^1^  B - Tita AT, 2008^1^ |
| **FÓRMULA DEL INDICADOR** | Número de mujeres a las que se prescribe antibiótico en alguna fase del parto y está registrado algún síntoma que lo justifique |
| **Numerador** |  |
| **Denominador** | Número de mujeres atendidas por motivo de parto con antibiótico prescrito |
| **FUENTE DE DATOS** | Expedientes clínicos  LVNS (si es caso) |
| **ELABORACIÓN DEL INDICADOR** | Propio |
| **REFERENCIAS BIBLIOGRÁFICAS**   - World Health Organization (WHO). WHO - Safe childbirth checklist [Internet]. Available from:   <http://www.who.int/patientsafety/implementation/checklists/childbirth/en/>   - - - 1. Guía de Práctica Clínica para la Reducción de la Frecuencia de Operación Cesárea México: Instituto Mexicano de Seguro social; 2014. ISBN: 978-607-7790-92-11   2. Organización Panamericana de la Salud “AIEPI Neonatal Intervenciones basadas en evidencia”. Segunda edición. Washington, D.C. 2010 ISBN: 978-92-75-33135-4 | |
| **OBSERVACIONES**  Los síntomas que pueden justificar la prescripción de antibiótico son dependientes de la fase de atención al parto:  **Etapa de admisión:** necesidad de antibiótico si la paciente presenta…   - Temperatura >38° - Flujo con olor desagradable (fétido) - Ruptura de membranas >18 horas - En trabajo de parto >24 horas   **Etapa pre-expulsiva:** necesidad de antibiótico si la paciente presenta…   - Temperatura >38° - Flujo con olor desagradable (fétido) - Ruptura de membranas >18 horas - En trabajo de parto >24 horas - Inicio de cesárea^1, 2^   **Etapa post-parto (<1 hora):** necesidad de antibiótico…   - si la placenta fue retirada manualmente - si hay temperatura >38° y cualquiera de los siguientes: escalofrío y/o flujo con olor desagradable (fétido)   **Etapa de antes del egreso:** necesidad de antibiótico si la paciente presenta…  - Temperatura >38° C y escalofrío o flujo con olor desagradable  - Temperatura >38° C y bajo tono/distensión abdominal  En el caso de la LVNS, se puede considerar que existe síntoma que justifica la prescripción con cualquier causa checada o descrita explícitamente. | |

| FICHA TÉCNICA DE INDICADORES: PROCESO | |
| --- | --- |
| **GRUPO:** | Buenas prácticas |
| **AREA:** | Generales |
| Sub-área | Uso de antibióticos |
| **Nº** | 3 (de 4) |
| **NOMBRE INDICADOR** | Prescripción de antibióticos en el recién nacido |
| **FORMA DE MEDICIÓN** | Muestreo de expedientes de mujeres atendidas por motivo de parto y sus recién nacido |
| **DESCRIPCIÓN** | Porcentaje de recién nacidos a quienes se prescribe antibiótico |
| **Nivel de evidencia; Fuerza recomendación** | D – NICE. Coto, 2006. Fernández, 2008. |
| **FÓRMULA DEL INDICADOR** | Número de recién nacidos a los que se prescribe antibiótico en cualquier momento hasta el egreso |
| **Numerador** |  |
| **Denominador** | Número de recién nacidos vivos |
| **FUENTE DE DATOS** | Expedientes clínicos madre y recién nacido  LVNS (si es caso) |
| **ELABORACIÓN DEL INDICADOR** | Propio |
| **REFERENCIAS BIBLIOGRÁFICAS**  World Health Organization (WHO). WHO - Safe childbirth checklist [Internet]. Available from: <http://www.who.int/patientsafety/implementation/checklists/childbirth/en/>  ^1^ Prevención, diagnóstico y tratamiento de sepsis y choque séptico del recién nacido en el segundo nivel y tercer nivel de atención, México: Secretaría de Salud; Noviembre 2012. | |
| **OBSERVACIONES**  Prescripción de antibiótico:  Será válida cualquier indicación o prescripción de antibiótico en el expediente clínico durante todo el proceso asistencial (desde el nacimiento hasta el egreso). Se capturará la fase del proceso asistencial (post-parto inmediato o antes del egreso) en el que se realiza la prescripción, si bien el indicador se reporta sin distinción de la fase.  Para fines de validación de la LVNS, se puede contrastar lo marcado en ésta independientemente de la causa que lo justifique, ya sea para el proceso global o por la fase de inicio (prescripción) de antibiótico. | |

| FICHA TÉCNICA DE INDICADORES: PROCESO | |
| --- | --- |
| **GRUPO:** | Buenas prácticas |
| **AREA:** | Generales |
| Sub-área | Uso de antibióticos |
| **Nº** | 4 (de 4) |
| **NOMBRE INDICADOR** | Manejo de antibióticos en el recién nacido |
| **FORMA DE MEDICIÓN** | Muestreo de expedientes de mujeres atendidas por motivo de parto y sus recién nacido |
| **DESCRIPCIÓN** | Porcentaje de recién nacidos a los que se prescribe antibiótico en cualquier momento y está registrado algún síntoma que lo justifique |
| **Nivel de evidencia; Fuerza recomendación** | D – NICE. Coto, 2006. Fernández, 2008. |
| **FÓRMULA DEL INDICADOR** | Número de recién nacidos a los que se prescribe antibiótico en cualquier momento y está registrado algún síntoma que lo justifique |
| **Numerador** |  |
| **Denominador** | Número de recién nacidos con antibiótico prescrito |
| **FUENTE DE DATOS** | Expedientes clínicos madre y recién nacido  LVNS (si es caso) |
| **ELABORACIÓN DEL INDICADOR** | Propio |
| **REFERENCIAS BIBLIOGRÁFICAS**   - World Health Organization (WHO). WHO - Safe childbirth checklist [Internet]. Available from:   <http://www.who.int/patientsafety/implementation/checklists/childbirth/en/>  ^1^ Prevención, diagnóstico y tratamiento de sepsis y choque séptico del recién nacido en el segundo nivel y tercer nivel de atención, México: Secretaría de Salud; noviembre 2012. | |
| **OBSERVACIONES**  necesidad de antibiótico en el recién nacido si:   - Respiración rápida (>60 respiraciones/min) o lenta (<30/min) - Pecho sumido, tiraje intercostal, ruido, convulsiones - Corioamnionitis - Movilidad escasa o nula a la estimulación - Se le dio antibiótico a la madre - Ruptura prematura de membranas (18h)^1^ - Temperatura muy fría (<35°C y no se calienta) o temperatura alta (>38°C)   En el caso de la LVNS, se puede considerar que existe síntoma que justifica la prescripción con cualquier causa checada o descrita explícitamente. | |

| FICHA TÉCNICA DE INDICADORES: PROCESO | |
| --- | --- |
| **GRUPO:** | Buenas prácticas |
| **AREA:** | Generales |
| Sub-área | Uso de sulfato de magnesio |
| **Nº** | 1 (de 2) |
| **NOMBRE INDICADOR** | Prescripción de sulfato de magnesio durante el parto |
| **FORMA DE MEDICIÓN** | Muestreo de expedientes de mujeres que fueron atendidas por motivo de parto |
| **DESCRIPCIÓN** | Porcentaje de mujeres a quienes se prescribe sulfato de magnesio durante el proceso del parto |
| **Nivel de evidencia; Fuerza recomendación** | No aplica |
| **FÓRMULA DEL INDICADOR** | Número de mujeres durante el proceso del parto a las que se prescribe sulfato de magnesio |
| **Numerador** |  |
| **Denominador** | Número de mujeres atendidas por motivo de parto |
| **FUENTE DE DATOS** | Expedientes clínicos  LVNS (si es caso) |
| **ELABORACIÓN DEL INDICADOR** | Propio |
| **REFERENCIAS BIBLIOGRÁFICAS**   - World Health Organization (WHO). WHO - Safe childbirth checklist [Internet]. Available from: <http://www.who.int/patientsafety/implementation/checklists/childbirth/en/> | |
| **OBSERVACIONES**  Prescripción de sulfato de magnesio:  Será válida cualquier indicación o prescripción de sulfato de magnesio en el expediente clínico durante todo el proceso del parto (desde la admisión hasta post-parto inmediato). Se capturará la fase del proceso asistencial (admisión, pre-expulsiva o post-parto inmediato) en el que se realiza la indicación o prescripción, si bien el indicador se reporta para el proceso global de atención al parto.  Para fines de validación de la LVNS, se puede contrastar lo marcado en ésta independientemente de la causa que lo justifique, ya sea para el proceso global o por la fase de inicio (prescripción) de sulfato de magnesio. | |

| FICHA TÉCNICA DE INDICADORES: PROCESO | |
| --- | --- |
| **GRUPO:** | Buenas prácticas |
| **AREA:** | Generales |
| Sub-área | Uso de sulfato de magnesio |
| **Nº** | 2 (de 2) |
| **NOMBRE DEL INDICADOR** | Manejo del sulfato de magnesio en el parto para control de preeclampsia/eclampsia |
| **FORMA DE MEDICIÓN** | Muestreo de expedientes de mujeres que fueron atendidas por motivo de parto y se les prescribió sulfato de magnesio |
| **DESCRIPCIÓN** | Porcentaje de mujeres a quienes se prescribe sulfato de magnesio durante el proceso del parto y está presente algún criterio que lo justifique |
| **Nivel de evidencia; Fuerza recomendación** | Evidencia: Ia-[E. Shekelle] - McDonald SD, 2012.- Duley L, 2010. (Enf. Eclampsia)  IV-[E. Shekelle] - Diemunsh P, et.al. 2010- (ER obst)  Recomendación: A-[E. Shekelle] - Duley L, 2010.(Enf. Eclampsia) |
| **FÓRMULA DEL INDICADOR** | Número de mujeres durante el proceso del parto a las que se prescribe sulfato de magnesio y está presente algún criterio de preeclampsia o eclampsia |
| **Numerador** |  |
| **Denominador** | Número de mujeres a las que, durante su estancia en el hospital, se prescribió sulfato de magnesio |
| **FUENTE DE DATOS** | Expedientes clínicos  LVNS (si es caso) |
| **ELABORACIÓN DEL INDICADOR** | Propio |
| **REFERENCIAS BIBLIOGRÁFICAS**   - World Health Organization (WHO). WHO - Safe childbirth checklist [Internet]. Available from:   <http://www.who.int/patientsafety/implementation/checklists/childbirth/en/>  1. Detección y Tratamiento Inicial de las Emergencias Obstétricas. México: Secretaria de Salud, elaboración 2010 y actualización 2011. | |
| **OBSERVACIONES**  **En todas las etapas:** necesidad de sulfato de magnesio si la paciente presenta:   - Presión diastólica ≥110 mmHg y proteinuria 3+ (ver tabla 1) - Presión diastólica ≥90 mmHg, proteinuria 2+ (ver tabla 1) y cualquiera de las siguientes: cefalea severa y/o visión borrosa - Dolor epigástrico   Es contraindicación para la prescripción de sulfato de magnesio (se considerará incumplimiento): insufiencia renal, lesión miocárdica, bloqueo cardíaco y miastenia gravis.  En el caso de la LVNS, se puede considerar que existe síntoma que justifica la prescripción con cualquier causa checada o descrita explícitamente. | |

**Tabla 1. Gradación de Proteinuria**

| **Designación** | **Cantidad aproximada** | |
| --- | --- | --- |
|  | **Concentración^1^** | **Diario^2^** |
| Traza | 5-20 mg/dL | - |
| 1+ | 30 mg/dL | Menos de 0.5 g/día |
| 2+ | 100 mg/dL | 0.5-1 g/día |
| 3+ | 300 mg/dL | 1-2 g/día |
| 4+ | Más de 300 mg/dL | Más de 2 g/día |

1. [eMedicine > Proteinuria](http://emedicine.medscape.com/article/984289-overview) Author: Ronald J Kallen. Coauthor: Watson C Arnold. Updated: Apr 21, 2008.

2. Ivanyi B, Kemeny E, Szederkenyi E, Marofka F, Szenohradszky P (December 2001). "The value of electron microscopy in the diagnosis of chronic renal allograft rejection". *Mod. Pathol.* **14** (12): 1200–8. doi:[10.1038/modpathol.3880461](http://dx.doi.org/10.1038%2Fmodpathol.3880461). PMID [11743041](http://www.ncbi.nlm.nih.gov/pubmed/11743041).

| FICHA TÉCNICA DE INDICADORES: PROCESO | |
| --- | --- |
| **GRUPO:** | Buenas prácticas |
| **AREA:** | Generales |
| Sub-área | Control de hemorragia |
| **Nº** | 1 (de 1) |
| **NOMBRE INDICADOR** | Atención correcta de la hemorragia después del parto |
| **FORMA DE MEDICIÓN** | Muestreo de expedientes de mujeres que fueron atendidas por motivo de parto |
| **DESCRIPCIÓN** | Porcentaje de pacientes en post-parto con manejo adecuado de hemorragia |
| **Nivel de evidencia; Fuerza recomendación** | Opinión de expertos |
| **FÓRMULA INDICADOR** | Número de mujeres en post parto a quienes se les realizan las acciones necesarias por sangrado o hemorragia |
| **Numerador** |  |
| **Denominador** | Número de mujeres en post parto que necesitaron acciones para manejo de hemorragia |
| **FUENTE DE DATOS** | Expedientes clínicos  LVNS (si es caso) |
| **ELABORACIÓN DEL INDICADOR** | Propio |
| **REFERENCIAS BIBLIOGRÁFICAS**   - World Health Organization (WHO). WHO - Safe childbirth checklist [Internet]. Available from:   <http://www.who.int/patientsafety/implementation/checklists/childbirth/en/>   - Organización Mundial de la Salud. Concentraciones de hemoglobina para diagnosticar la anemia y evaluar su gravedad. Ginebra, Organización Mundial de la Salud, 2011 (WHO/NMH/NHD/MNM/11.1) <http://www.who.int/vmnis/indicators/haemoglobin_es.pdf> (consultado el 08/07/2015). - Prevención y manejo de la hemorragia obstétrica en el primer, segundo y tercer niveles de atención – SS-103-08. Secretaría de salud. México. Actualización 2013. - Biblioteca de Salud Reproductiva de la OMS. Tratamiento para la hemorragia postparto primaria. Available from: <http://apps.who.int/rhl/pregnancy_childbirth/childbirth/postpartum_haemorrhage/sfguide/es/>   (consultado el 23/07/2015). | |
| **OBSERVACIONES**  **INDICADOR COMPUESTO**  **Etapa post-parto inmediato:**  Se valora independientemente cada una de las acciones a realizar en esta etapa. Reporte de las acciones en el post parto si hay hemorragia ≥500 ml, o si ≥250 ml y anemia severa (hemoglobina menor a 7 gr/dL)  Se considerará la revisión en la LVNS y en el expediente de cada acción y del conjunto (compuesto):   - Masaje al útero / LVNS y expedientes revisados - Considerar uterotónico adicional/ LVNS y expedientes revisados - Iniciar vía intravenosa/ LVNS y expedientes revisados - Activar equipo de respuesta rápida para emergencias obstétricas/ LVNS y expedientes revisados - Tratar la causa/ LVNS y expedientes revisados - Las 5 actividades realizadas/ LVNS y expedientes revisados (compuesto)   **Etapa antes de egreso:**   - Sangrado excesivo: hemorragia ≥500 ml, o si ≥250 ml y anemia severa (hemoglobina menor a 7 gr/dL). - Si la paciente presenta hemorragia no controlada, se debe tratar y retrasar el egreso (expediente con “hemorragia controlada” registrado antes del egreso o checado en LVNS). | |

## 3.2. Indicadores para fase de admisión

| FICHA TÉCNICA DE INDICADORES: PROCESO | |
| --- | --- |
| **GRUPO:** | Buenas prácticas |
| **AREA:** | Admisión |
| Sub-área | Uso de partograma |
| **Nº** | 1 (de 2) |
| **NOMBRE INDICADOR** | Apertura de partograma |
| **FORMA DE MEDICIÓN** | Muestreo de expedientes de mujeres que fueron atendidas por motivo de parto |
| **DESCRIPCIÓN** | Porcentaje de mujeres a las que se les realizó apertura de partograma |
| **Nivel de evidencia; Fuerza recomendación** | A – Shekelle – Sony OMS, 2009  D - Shekelle- NOM-007-SSA2-1993 |
| **FÓRMULA DEL INDICADOR** | Número de mujeres con partograma iniciado |
| **Numerador** |  |
| **Denominador** | Número de mujeres que fueron atendidas por motivo de parto |
| **FUENTE DE DATOS** | Expedientes clínicos: partograma |
| **ELABORACIÓN DEL INDICADOR** | Propio |
| **REFERENCIAS BIBLIOGRÁFICAS**   - World Health Organization (WHO). WHO - Safe childbirth checklist [Internet]. Available from:   <http://www.who.int/patientsafety/implementation/checklists/childbirth/en/>   - Vigilancia y manejo del trabajo de parto en embarazo de bajo riesgo. México: Secretaría de Salud; 11 de diciembre de 2014. ISBN: 978-607-7790-94-5 - Norma Oficial Mexicana NOM-007-SSA2-1993, Atención de la mujer durante el embarazo, parto y puerperio y del recién nacido. Criterios y procedimientos para la prestación del servicio. | |
| **OBSERVACIONES**  **Apertura:** existe el partograma en el expediente con datos de la paciente (al menos, nombre completo, edad o fecha de nacimiento, semanas de gestación y nº expediente clínico). | |

| FICHA TÉCNICA DE INDICADORES: PROCESO | |
| --- | --- |
| **GRUPO:** | Buenas prácticas |
| **AREA:** | Admisión |
| Sub-área | Uso de partograma |
| **Nº** | 2 (de 2) |
| **NOMBRE INDICADOR** | Llenado del partograma |
| **FORMA DE MEDICIÓN** | Muestreo de expedientes de mujeres que fueron atendidas por motivo de parto |
| **DESCRIPCIÓN** | Porcentaje de mujeres a las que se les realizó rellenado de partograma en el parto |
| **Nivel de evidencia; Fuerza recomendación** | A – Shekelle – Sony OMS, 2009  D - Shekelle- NOM-007-SSA2-1993 |
| **FÓRMULA DEL INDICADOR** | Número de mujeres con partograma rellenado correctamente en el expediente |
| **Numerador** |  |
| **Denominador** | Número de mujeres que fueron atendidas por motivo de parto y tenían partograma en el expediente |
| **FUENTE DE DATOS** | Expedientes clínicos: partograma  LVNS (si es caso) |
| **ELABORACIÓN DEL INDICADOR** | Propio |
| **REFERENCIAS BIBLIOGRÁFICAS**   - World Health Organization (WHO). WHO - Safe childbirth checklist [Internet]. Available from:   <http://www.who.int/patientsafety/implementation/checklists/childbirth/en/>   - Vigilancia y manejo del trabajo de parto en embarazo de bajo riesgo. México: Secretaría de Salud; 11 de diciembre de 2014. ISBN: 978-607-7790-94-5 - Norma Oficial Mexicana NOM-007-SSA2-1993, Atención de la mujer durante el embarazo, parto y puerperio y del recién nacido. Criterios y procedimientos para la prestación del servicio. | |
| **OBSERVACIONES**  **Llenado:**  - Cada 2 horas: temperatura.  - Cada 30 min: frecuencia cardiaca de la mujer y del feto, y las contracciones.  - Cada 4 horas: presión arterial.   - Tacto/dilatación según evolución e indicación médica: debe haber chequeo explícito en LVNS y/o registro en el expediente de tacto/dilatación (el registro de cm de dilatación se tomará como válido) | |

| FICHA TÉCNICA DE INDICADORES: PROCESO | |
| --- | --- |
| **GRUPO:** | Buenas prácticas |
| **AREA:** | Admisión |
| Sub-área | Uso de antiretrovirales |
| **Nº** | 1 (de 1) |
| **NOMBRE INDICADOR** | Manejo de anti-retrovirales en el parto |
| **FORMA DE MEDICIÓN** | Revisión de expedientes de mujeres con VIH atendidas por motivo de parto |
| **DESCRIPCIÓN** | Porcentaje de mujeres con VIH en trabajo de parto en las que está iniciado el tratamiento anti-retroviral |
| **Nivel de evidencia; Fuerza recomendación** | A: [E. Shekelle]. Volmink, J. 2009 |
| **FÓRMULA DEL INDICADOR** | Número de mujeres (VIH+) en las que se ha iniciado anti-retroviral durante el trabajo de parto |
| **Numerador** |  |
| **Denominador** | Número de mujeres con VIH+ atendidas por motivo de parto |
| **FUENTE DE DATOS** | Expedientes clínicos  LVNS (si es caso) |
| **ELABORACIÓN DEL INDICADOR** | Propio |
| **REFERENCIAS BIBLIOGRÁFICAS**   - World Health Organization (WHO). WHO - Safe childbirth checklist [Internet]. Available from:   <http://www.who.int/patientsafety/implementation/checklists/childbirth/en/>   - Prevención, diagnóstico y tratamiento del binomio madre-hijo con infección por el VIH. México. Instituto Mexicano del Seguro Social, 2009; actualización junio 2012 | |
| **OBSERVACIONES**  Para la prevención de la transmisión de VIH de la madre al recién nacido durante el parto, el tratamiento antirretroviral debe administrarse desde el inicio del trabajo de parto (o en cualquier momento desde la admisión de la mujer en trabajo de parto) hasta el nacimiento del recién nacido.  En la LVNS, el inicio de anti-retrovirales (ARV) debe estar registrado en la fase de admisión  El expediente clínico de la madre debe estar registrada la prescripción del ARV siempre antes del nacimiento. | |

| FICHA TÉCNICA DE INDICADORES: PROCESO | |
| --- | --- |
| **GRUPO:** | Buenas prácticas |
| **AREA:** | Admisión |
| Sub-área | Atención centrada en el paciente |
| **Nº** | 1 (de 1) |
| **NOMBRE INDICADOR** | Promoción de presencia de acompañante durante el parto |
| **FORMA DE MEDICIÓN** | Encuesta de mujeres en postparto |
| **DESCRIPCIÓN** | Porcentaje de mujeres en trabajo de parto a cuyo acompañante se le informa y anima a estar presente en el parto |
| **Nivel de evidencia; Fuerza recomendación** | Ia-[E. Shekelle] – Hodnett. Cochrane 2013.  IV-[E. Shekelle] – Martis BSR OMS 2007.  A-GPC Ministerio de Salud, 2010. |
| **FÓRMULA DEL INDICADOR** | N° de mujeres en trabajo de parto a cuyo acompañante se le informa y anima a estar presente en el parto desde el ingreso |
| **Numerador** |  |
| **Denominador** | Número de mujeres que fueron atendidas por motivo de parto |
| **FUENTE DE DATOS** | Cuestionario a la madre  LVNS (si es caso) |
| **ELABORACIÓN DEL INDICADOR** | Propio |
| **REFERENCIAS BIBLIOGRÁFICAS**   - World Health Organization (WHO). WHO - Safe childbirth checklist [Internet]. Available from:   <http://www.who.int/patientsafety/implementation/checklists/childbirth/en/>   - Vigilancia y manejo del trabajo de parto en embarazo de bajo riesgo. México: Secretaría de Salud; 11 de diciembre de 2014. ISBN: 978-607-7790-94-5   ^1^ Biblioteca de Salud reproductiva de la OMS. Apoyo continuo a las mujeres durante el parto. http://apps.who.int/rhl/pregnancy_childbirth/childbirth/routine_care/rmcom/es/ | |
| **OBSERVACIONES**  Si la madre está en condiciones de decidir, ella será quien determine quién es su acompañante; siempre y cuando el hospital cuente con la estructura necesaria para permitir la presencia del acompañante.  Son motivo de excepción todos los casos en los que no había ningún acompañante o la estructura del centro no lo permite.  No obstante la OMS señala^1^:  “El argumento de que invade la privacidad de otras mujeres a menudo no puede sostenerse, ya que otros miembros del personal como por ejemplo el personal de limpieza, el servicio de comidas y los estudiantes también ingresan a la sala de preparto sin presentarse y podrían invadir la privacidad de todas las mujeres. La mayoría de los hospitales ofrecen cortinas que pueden utilizarse si la privacidad constituye un verdadero problema”. | |

## 3.3. Indicadores para fase pre-expulsiva (o antes de cesárea)

| FICHA TÉCNICA DE INDICADORES: PROCESO | |
| --- | --- |
| **GRUPO:** | Buenas prácticas |
| **AREA:** | Fase pre-expulsiva |
| Sub-área | Intervenciones en el parto |
| **Nº** | 1 (de 3) |
| **NOMBRE DEL INDICADOR** | Resolución del parto por cesárea justificada |
| **FORMA DE MEDICIÓN** | Muestreo de expedientes de mujeres que fueron atendidas por motivo de parto a las que se les realiza cesárea |
| **DESCRIPCIÓN** | Porcentaje de mujeres con resolución del parto por cesárea justificada |
| **Nivel de evidencia; Fuerza recomendación** | Opinión de Expertos |
| **FÓRMULA DEL INDICADOR** | Número de mujeres a las que se les realiza cesárea bajo una indicación justificada |
| **Numerador** |  |
| **Denominador** | Número de mujeres a las que les realiza cesárea |
| **FUENTE DE DATOS** | Expedientes clínicos  LVNS (si es caso) |
| **ELABORACIÓN DEL INDICADOR** | Propio |
| **REFERENCIAS BIBLIOGRÁFICAS**   - Guía de Práctica Clínica para la Reducción de la Frecuencia de Operación Cesárea México: Instituto Mexicano de Seguro social; 2014. ISBN: 978-607-7790-92-1 | |
| **OBSERVACIONES**  Indicación correcta y justificada para realizar cesárea:   - 2 cesáreas previas - situación transversa - embarazo gemelar - presentación pélvica - cardiopatía clase III y IV - hidrocefalia fetal - placenta previa total - cerclaje vía abdominal - macrosomía - estado fetal inestable - malformaciones fetales - herpes genital activo - tumor que obstruya el canal de parto - antecedente de cirugía uterina - desprendimiento prematuro de placenta normoinserta - VIH - producto óbito >30 semanas de gestación (SDG) en paciente sin trabajo de parto por más de 24 hrs. | |

| FICHA TÉCNICA DE INDICADORES: PROCESO | |
| --- | --- |
| **GRUPO:** | Buenas prácticas |
| **AREA:** | Fase pre-expulsiva |
| Sub-área | Intervenciones en el parto |
| **Nº** | 2 (de 3) |
| **NOMBRE DEL INDICADOR** | Realización de parto instrumentado justificado |
| **FORMA DE MEDICIÓN** | Muestreo de expedientes de mujeres que fueron atendidas por motivo de parto a las que se les realiza parto instrumentado |
| **DESCRIPCIÓN** | Porcentaje de mujeres con parto instrumentado justificado |
| **Nivel de evidencia; Fuerza recomendación** | IIb - Guía de Práctica Clínica- Parto Instrumental. 2010  C - ACOG, 2004 |
| **FÓRMULA DEL INDICADOR** | Número de mujeres con indicación justificada de parto instrumentado |
| **Numerador** |  |
| **Denominador** | Número de mujeres con parto instrumentado |
| **FUENTE DE DATOS** | Expedientes clínicos  LVNS (si es caso) |
| **ELABORACIÓN DEL INDICADOR** | Propio |
| **REFERENCIAS BIBLIOGRÁFICAS**  Guía de Práctica Clínica para la Reducción de la Frecuencia de Operación Cesárea México: Instituto Mexicano de Seguro social; 2014. ISBN: 978-607-7790-92-1 | |
| **OBSERVACIONES**  Indicación correcta y justificada para parto instrumentado (forceps):  Causas fetales:   - Presunto compromiso fetal/expulsivo con inestabilidad fetal   Causas maternas:   - Enfermedades médicas para evitar valsalva (Ej. Enfermedad materna Clase funcional III-IV) - Expulsivo Prolongado - Fatiga/agotamiento materna - Cesárea previa - Cardiopatía materna | |

| FICHA TÉCNICA DE INDICADORES: PROCESO | |
| --- | --- |
| **GRUPO:** | Buenas prácticas |
| **AREA:** | Fase pre-expulsiva |
| Sub-área | Intervenciones en el parto |
| **Nº** | 3 (de 3) |
| **NOMBRE DEL INDICADOR** | Realización de episiotomía justificada |
| **FORMA DE MEDICIÓN** | Muestreo de expedientes de mujeres que fueron atendidas por motivo de parto a las que se les realiza episiotomía |
| **DESCRIPCIÓN** | Porcentaje de mujeres con episiotomía justificada en el parto |
| **Nivel de evidencia; Fuerza recomendación** | A - GPC Ministerio de Sanidad y Política Social, 2010  A - GPC Prevención, diagnóstico y tratamiento de la episiotomías complicada IMSS, 2013 |
| **FÓRMULA DEL INDICADOR** | Número de mujeres con indicación justificada de episiotomía |
| **Numerador** |  |
| **Denominador** | Número de mujeres con episiotomía en el parto |
| **FUENTE DE DATOS** | Expedientes clínicos  LVNS (si es caso) |
| **ELABORACIÓN DEL INDICADOR** | Propio |
| **REFERENCIAS BIBLIOGRÁFICAS**   - Vigilancia y manejo del trabajo de parto en embarazo de bajo riesgo. México: Secretaría de Salud; 11 de diciembre de 2014. ISBN: 978-607-7790-94-5 - Norma Oficial Mexicana NOM-007-SSA2-1993, Atención de la mujer durante el embarazo, parto y puerperio y del recién nacido. Criterios y procedimientos para la prestación del servicio. | |
| **OBSERVACIONES**  No debe practicarse episiotomía de rutina en todos los partos espontáneos.  La episiotomía deberá realizarse si hay necesidad clínica, por lo que se considerarán los siguientes:   - Parto instrumental - Periné corto y/o rígido - Distocia de hombros en el feto | |

| FICHA TÉCNICA DE INDICADORES: ESTRUCTURA | |
| --- | --- |
| **GRUPO:** | Buenas prácticas |
| **AREA:** | Fase pre-expulsiva |
| Sub-área | Infraestructura |
| **Nº** | 1 (de 2) |
| **NOMBRE INDICADOR** | Disponibilidad de insumos para atención de la madre inmediatamente antes del parto |
| **FORMA DE MEDICIÓN** | Muestreo de expedientes de mujeres que fueron atendidas por motivo de parto: revisión de LVNS ; o realización de encuesta |
| **DESCRIPCIÓN** | Disponibilidad de insumos para atención de la madre inmediatamente antes del parto |
| **Nivel de evidencia; Fuerza recomendación** | No aplica |
| **FÓRMULA INDICADOR** | Disponibilidad de insumos para atención de la madre (cada insumo y del conjunto) |
| **Numerador** |  |
| **Denominador** | Número de LVNS revisadas |
| **FUENTE DE DATOS** | LVNS  Cuestionario *ad hoc* a profesionales |
| **ELABORACIÓN** | Propio |
| **REFERENCIAS BIBLIOGRÁFICAS**  World Health Organization (WHO). WHO - Safe childbirth checklist [Internet]. Available from:  <http://www.who.int/patientsafety/implementation/checklists/childbirth/en/> | |
| **OBSERVACIONES**  Reporte de disponibilidad de cada insumo y del conjunto (100%):   - Agua limpia - Jabón - Toallas desechables (sanitas) - Guantes - Uterotónico: oxitocina 10 IU en jeringa n° de LVNS revisadas - 2 Pinzas de anillos   Herramienta de validación de información: encuesta a profesionales  La categoría de respuesta “siempre” será tenida en cuenta como insumo disponible.  El resto de categorías serán tenidas en cuenta como no disponibilidad de insumos. | |

| FICHA TÉCNICA DE INDICADORES: ESTRUCTURA | |
| --- | --- |
| **GRUPO:** | Buenas prácticas |
| **AREA:** | Fase pre-expulsiva |
| Sub-área | Infraestructura |
| **Nº** | 2 (de 2) |
| **NOMBRE DEL INDICADOR** | Disponibilidad de insumos para atención del recién nacido inmediatamente antes del parto |
| **FORMA DE MEDICIÓN** | Muestreo de expedientes de mujeres que fueron atendidas por motivo de parto: revisión de LVNS ; o realización de encuesta |
| **DESCRIPCIÓN** | Disponibilidad de insumos para atención del recién nacido inmediatamente antes del parto |
| **Nivel de evidencia; Fuerza recomendación** | No aplica |
| **FÓRMULA INDICADOR** | Disponibilidad de insumos para atención del recién nacido (cada insumo y del conjunto) |
| **Numerador** |  |
| **Denominador** | Número de LVNS revisadas |
| **FUENTE DE DATOS** | LVNS  Cuestionario *ad hoc* a profesionales |
| **ELABORACIÓNINDICADOR** | Propio |
| **REFERENCIAS BIBLIOGRÁFICAS**   - World Health Organization (WHO). WHO - Safe childbirth checklist [Internet]. Available from: <http://www.who.int/patientsafety/implementation/checklists/childbirth/en/> - Atención del recién nacido sano; México: Secretaría de Salud; 2009 | |
| **OBSERVACIONES**  Reporte de disponibilidad de la LVNS: de cada insumo y del conjunto (100%):   - Toallas limpias o campos - Tijera para corte de cordón - Perilla de succión - Vitamina K - Profilaxis oftálmica - Equipo de canalización - Laringoscopio - Cinta para cordón umbilical n° de LVNS revisadas - Fuente de O2 y aspiración - Adrenalina - Cánulas de intubación - Solución salina y glucosada - Mascarilla y bolsa para ventilación (ambú)   Herramienta de validación de información: encuesta a profesionales  La categoría de respuesta “siempre” será tenida en cuenta como insumo disponible.  El resto de categorías serán tenidas en cuenta como no disponibilidad de insumos. | |

## 3.4. Indicadores para fase Post-parto inmediato (primera hora tras la expulsión)

| FICHA TÉCNICA DE INDICADORES: PROCESO | |
| --- | --- |
| **GRUPO:** | Buenas prácticas |
| **AREA:** | Post-parto inmediato |
| Sub-área | Atención de la madre |
| **Nº** | 1 (de 2) |
| **NOMBRE DEL INDICADOR** | Atención a la madre inmediatamente después del nacimiento |
| **FORMA DE MEDICIÓN** | Muestreo de expedientes de mujeres que fueron atendidas por motivo de parto: revisión de LVNS |
| **DESCRIPCIÓN** | Porcentaje de mujeres a las que se les realizaron acciones necesarias inmediatamente después del nacimiento. |
| **Nivel de evidencia; Fuerza recomendación** | A, B - (GPC Ministerio de Sanidad y Política Social, 2010) - Fuerte (WHO recommendations for the prevention and treatment of postpartum haemorrhage, 2012).  D - Shekelle - FIGO Guidelines. Prevention and treatment of postpartum hemorrhage in low-resource settings, 2012. |
| **FÓRMULA INDICADOR** | Mujeres a las que se les realizaron las acciones necesarias inmediatamente después del nacimiento (ver observaciones) |
| **Numerador** |  |
| **Denominador** | Total expedientes revisados |
| **FUENTE DE DATOS** | Expediente clínico  LVNS |
| **ELABORACIÓN INDICADOR** | Propio |
| **REFERENCIAS BIBLIOGRÁFICAS**  ^1^ World Health Organization (WHO). WHO - Safe childbirth checklist [Internet]. Available from: <http://www.who.int/patientsafety/implementation/checklists/childbirth/en/>  ^2^ Vigilancia y manejo del trabajo de parto en embarazo de bajo riesgo. México: Secretaría de Salud; 11 de diciembre de 2014. ISBN: 978-607-7790-94-5 | |
| **OBSERVACIONES**  Reporte de las acciones necesarias de atención a la madre inmediatamente después del nacimiento   - Administración de **Oxitocina en el primer minuto^1,2^**/ LVNS y expedientes revisados - **Controlar tracción del cordón umbilical^1,2^** para la extracción de la placenta/ LVNS y expedientes revisados - **Masaje de útero^1,2^** después de extraer la placenta/ LVNS y expedientes revisados   Valorar % de cumplimiento de cada una de las acciones.  Valorar % de cumplimiento del indicador compuesto. Es necesario el cumplimiento del 100% de los cuatro componentes. | |

| FICHA TÉCNICA DE INDICADORES: PROCESO | |
| --- | --- |
| **GRUPO:** | Buenas prácticas |
| **AREA:** | Post-parto inmediato |
| Sub-área | Atención de la madre |
| **Nº** | 2 (de 2) |
| **NOMBRE DEL INDICADOR** | Descartar presencia de segundo producto |
| **FORMA DE MEDICIÓN** | Muestreo de expedientes de mujeres que fueron atendidas por motivo de parto: revisión de LVNS |
| **DESCRIPCIÓN** | Porcentaje de mujeres en las que la presencia de un segundo producto es revisada y descartada |
| **Nivel de evidencia; Fuerza recomendación** |  |
| **FÓRMULA INDICADOR** | Mujeres en las que se revisa y descarta presencia de un segundo producto |
| **Numerador** |  |
| **Denominador** | Total expedientes revisados |
| **FUENTE DE DATOS** | Expediente clínico  LVNS |
| **ELABORACIÓN INDICADOR** | Propio |
| **REFERENCIAS BIBLIOGRÁFICAS**  ^1^ World Health Organization (WHO). WHO - Safe childbirth checklist [Internet]. Available from: <http://www.who.int/patientsafety/implementation/checklists/childbirth/en/> | |
| **OBSERVACIONES**  Reporte de las acciones necesarias de atención a la madre inmediatamente después del nacimiento   - Descartar **presencia de un segundo bebé^1^**/ LVNS y expedientes revisados | |

| FICHA TÉCNICA DE INDICADORES: PROCESO | |
| --- | --- |
| **GRUPO:** | Buenas prácticas |
| **AREA:** | Post-parto inmediato |
| Sub-área | Atención del recién nacido |
| **Nº** | 1 (de 5) |
| **NOMBRE INDICADOR** | Cuidado inmediato del recién nacido |
| **FORMA DE MEDICIÓN** | Muestreo de expedientes de mujeres atendidas por motivo de parto y sus recién nacidos |
| **DESCRIPCIÓN** | Porcentaje de recién nacido a los que se les realizaron acciones necesarias de cuidado |
| **Nivel de evidencia; Fuerza recomendación** | **Profilaxis oftálmica**  A, C – NICE, 2006.  **Vitamina K**  A, D – NICE, 2006.  **Secado**  A – NICE, 2006. |
| **FÓRMULA DEL INDICADOR** | Total de recién nacidos a los que se les realizaron correctamente las acciones necesarias en el post-parto inmediato |
| **Numerador** |  |
| **Denominador** | Número de recién nacidos |
| **FUENTE DE DATOS** | Expediente clínico de la madre; Hoja del recién nacido; LVNS (si es caso) |
| **ELABORACIÓN DEL INDICADOR** | Propio |
| **REFERENCIAS BIBLIOGRÁFICAS**   - World Health Organization (WHO). WHO - Safe childbirth checklist [Internet]. Available from: <http://www.who.int/patientsafety/implementation/checklists/childbirth/en/>   ^1^ Vigilancia y manejo del trabajo de parto en embarazo de bajo riesgo. México: Secretaría de Salud; 2014. ISBN: 978-607-7790-94-5  ^2^ Atención del recién nacido sano; México: Secretaría de Salud; 2009.  ^3^ Norma Oficial Mexicana NOM-007-SSA2-1993, Atención de la mujer durante el embarazo, parto y puerperio y del recién nacido. Criterios y procedimientos para la prestación del servicio. | |
| **OBSERVACIONES**  Valorar % de cumplimiento de cada una de las acciones. Valorar % de cumplimiento del indicador compuesto (cumplimiento del 100% de los cuatro componentes).  Cuidado adecuado del recién nacido:   - **Secar al bebé^1,2^** y mantenerlo caliente^2^ - **Administrar vitamina K^2,3^** - **Administrar profilaxis oftálmica^2^** | |

| FICHA TÉCNICA DE INDICADORES: PROCESO | |
| --- | --- |
| **GRUPO:** | Buenas prácticas |
| **AREA:** | Post-parto inmediato |
| Sub-área | Atención del recién nacido |
| **Nº** | 2 (de 5) |
| **NOMBRE INDICADOR** | Pinzamiento tardío del cordón umbilical en el recién nacido |
| **FORMA DE MEDICIÓN** | Muestreo de expedientes de mujeres atendidas por motivo de parto y sus recién nacidos |
| **DESCRIPCIÓN** | Porcentaje de recién nacido a los que se les realizó el pinzamiento tardío del recién nacido |
| **Nivel de evidencia; Fuerza recomendación** | **Retraso en cortar el cordón**  1+; GPC Ministerio de Sanidad y Política Social, 2010  IV; Shekelle - FIGO Guidelines. Prevention and treatment of postpartum hemorrhage in low-resource settings, 2012 |
| **FÓRMULA DEL INDICADOR** | Total de recién nacidos a los que se les realizó el pinzamiento tardío del recién nacido como parte de las acciones del post-parto inmediato |
| **Numerador** |  |
| **Denominador** | Número de recién nacidos |
| **FUENTE DE DATOS** | Expediente clínico de la madre; Hoja del recién nacido; LVNS (si es caso) |
| **ELABORACIÓN DEL INDICADOR** | Propio |
| **REFERENCIAS BIBLIOGRÁFICAS**   - World Health Organization (WHO). WHO - Safe childbirth checklist [Internet]. Available from: <http://www.who.int/patientsafety/implementation/checklists/childbirth/en/>   ^1^ Vigilancia y manejo del trabajo de parto en embarazo de bajo riesgo. México: Secretaría de Salud; 2014. ISBN: 978-607-7790-94-5 | |
| **OBSERVACIONES**   - **Retraso en cortar el cordón^1^ -** Está recomendado el pinzamiento tardío del cordón umbilical en el manejo activo del tercer período de trabajo de parto (1 a 3 minutos después del nacimiento) o al cese del latido del cordón umbilical.   **Criterio de exclusión**: Inestabilidad de la madre y/o el recién nacido. | |

| FICHA TÉCNICA DE INDICADORES: PROCESO | |
| --- | --- |
| **GRUPO:** | Buenas prácticas |
| **AREA:** | Post-parto inmediato |
| Sub-área | Atención del recién nacido |
| **Nº** | 3 (de 5) |
| **NOMBRE INDICADOR** | Contacto piel con piel |
| **FORMA DE MEDICIÓN** | Muestreo de expedientes de mujeres atendidas por motivo de parto y sus recién nacidos |
| **DESCRIPCIÓN** | Porcentaje de recién nacido en los que se inicia el contacto piel con piel de manera inmediata |
| **Nivel de evidencia; Fuerza recomendación** | **Piel con piel inmediato**  A - Shekelle. Moore, 2012  A - Guía de Práctica Clínicas sobre la Atención al Parto Normal, Ministerio de Sanidad y Consumo de España, 2010. |
| **FÓRMULA DEL INDICADOR** | Total de recién nacidos en los que se inicia el contacto piel con piel de manera inmediata tras el nacimiento |
| **Numerador** |  |
| **Denominador** | Número de recién nacidos |
| **FUENTE DE DATOS** | Expediente clínico de la madre; Hoja del recién nacido; LVNS (si es caso) |
| **ELABORACIÓN DEL INDICADOR** | Propio |
| **REFERENCIAS BIBLIOGRÁFICAS**   - World Health Organization (WHO). WHO - Safe childbirth checklist [[ - Internet]. Available from: <http://www.who.int/patientsafety/implementation/checklists/childbirth/en/>   ^1^ Atención del recién nacido sano; México: Secretaría de Salud; 2009  ^2^ Vigilancia y manejo del trabajo de parto en embarazo de bajo riesgo. México: Secretaría de Salud; 2014. ISBN: 978-607-7790-94-5  ^3^ Atención del recién nacido sano; México: Secretaría de Salud; 2009. | |
| **OBSERVACIONES**   - **Piel con piel inmediato^1, 2, 3^ -** Se recomienda el contacto temprano piel-piel de madres y recién nacidos sanos post-nacimiento, pues mejora la estabilidad cardiorrespiratoria e incrementa la glucosa en los recién nacidos (recién nacido). Y que permanezca junto a su madre en todo momento tras el parto, siempre y cuando el estado de salud de la madre y del recién nacido lo permitan. Si el estado de salud de la madre no lo permite, se ofrecerá al padre la posibilidad de que haga el contacto piel con piel con el recién nacido.   **Criterio de exclusión**: Inestabilidad de la madre y/o el recién nacido.  Herramienta de validación: encuesta a madres. | |

| FICHA TÉCNICA DE INDICADORES: PROCESO | |
| --- | --- |
| **GRUPO:** | Buenas prácticas |
| **AREA:** | Post-parto inmediato |
| Sub-área | Atención del recién nacido |
| **Nº** | 4 (de 5) |
| **NOMBRE INDICADOR** | Lactancia materna |
| **FORMA DE MEDICIÓN** | Muestreo de expedientes de mujeres atendidas por motivo de parto y sus recién nacidos |
| **DESCRIPCIÓN** | Porcentaje de recién nacido que inicial la lactancia materna inmediata |
| **Nivel de evidencia; Fuerza recomendación** | **LM inmediata**  D – Shekelle. PROYECTO de Norma Oficial Mexicana PROY-NOM-007-SSA2-2010, Para la atención de la mujer durante el embarazo, parto y puerperio, y del recién nacido, 2012. |
| **FÓRMULA DEL INDICADOR** | Total de recién nacidos en los que se inicia la lactancia materna inmediatamente después del parto (primera hora tras la expulsión) |
| **Numerador** |  |
| **Denominador** | Número de recién nacidos |
| **FUENTE DE DATOS** | Expediente clínico de la madre; Hoja del recién nacido; LVNS (si es caso) |
| **ELABORACIÓN DEL INDICADOR** | Propio |
| **REFERENCIAS BIBLIOGRÁFICAS**   - World Health Organization (WHO). WHO - Safe childbirth checklist [Internet]. Available from: <http://www.who.int/patientsafety/implementation/checklists/childbirth/en/>   ^1^ Atención del recién nacido sano; México: Secretaría de Salud; 2009  ^2^ Vigilancia y manejo del trabajo de parto en embarazo de bajo riesgo. México: Secretaría de Salud; 2014. ISBN: 978-607-7790-94-5  ^3^ Atención del recién nacido sano; México: Secretaría de Salud; 2009. | |
| **OBSERVACIONES**   - **LM inmediata (> 1h)^1, 2, 3^ -** Iniciar la lactancia materna exclusiva a libre demanda dentro de los primeros 30 minutos de vida del recién nacido, en aquellas mujeres cuyas condiciones lo permitan.   **Criterio de exclusión**: Inestabilidad de la madre y/o el recién nacido. No iniciar o evitar LM en el caso de madres con VIH+ o en tratamiento con medicamento contraindicado para LM  Herramienta de validación: encuesta a madres. | |

| FICHA TÉCNICA DE INDICADORES: PROCESO | |
| --- | --- |
| **GRUPO:** | Buenas prácticas |
| **AREA:** | Post-parto inmediato |
| Sub-área | Atención del recién nacido |
| **Nº** | 5 (de 5) |
| **NOMBRE INDICADOR** | Manejo de anti-retrovirales en el recién nacido |
| **FORMA DE MEDICIÓN** | Muestreo o total de expedientes de mujeres con VIH que fueron atendidas por motivo de parto y sus recién nacido |
| **DESCRIPCIÓN** | Porcentaje de recién nacidos con tratamiento anti-retroviral iniciado |
| **Nivel de evidencia; Fuerza recomendación** | C – Shekelle. Public Health Service Task Force Recommendations for Use of Antiretroviral Drugs in Pregnant HIV-Infected Women for materna Health and Interventions to Reduce Perinatal HIV Transmission in the United States, 2009. |
| **FÓRMULA DEL INDICADOR** | Número de recién nacidos en los que se inicia tratamiento con anti-retroviral |
| **Numerador** |  |
| **Denominador** | Número total de recién nacidos de madres con VIH+ |
| **FUENTE DE DATOS** | Expedientes clínicos / Historia del recién nacido  LVNS (si es caso) |
| **ELABORACIÓN DEL INDICADOR** | Propio |
| **REFERENCIAS BIBLIOGRÁFICAS**   - World Health Organization (WHO). WHO - Safe childbirth checklist [Internet]. Available from: <http://www.who.int/patientsafety/implementation/checklists/childbirth/en/> - WHO recommendations for Prevention and treatment of pre-eclampsia and eclampsia. Geneva: World Health Organization, 2011. - Prevención, diagnóstico y tratamiento en el binomio madre-hijo con infección por el VIH. México, Instituto Mexicano del Seguro Social, 2009; actualización en junio, 2012. | |
| **OBSERVACIONES**  El recién nacido debe recibir tratamiento en las primeras 6h si la madre presenta VIH+. | |

## 3.5. Indicadores para fase justo antes del alta

| FICHA TÉCNICA DE INDICADORES: PROCESO | |
| --- | --- |
| **GRUPO:** | Buenas prácticas |
| **AREA:** | Antes del egreso |
| Sub-área | Atención de la madre |
| **Nº** | 1 (de 2) |
| **NOMBRE INDICADOR** | Información sobre planificación familiar previo al egreso |
| **FORMA DE MEDICIÓN** | Muestreo de expedientes de mujeres atendidas por motivo de parto;  Realización de encuesta |
| **DESCRIPCIÓN** | Porcentaje de mujeres con información sobre planificación familiar antes de su egreso |
| **Nivel de evidencia; Fuerza recomendación** | No encontrada |
| **FÓRMULA DEL INDICADOR** | Número de mujeres a las que se informa, explica, o se le presentan opciones sobre planificación familiar previo a su egreso |
| **Numerador** |  |
| **Denominador** | Número total de mujeres egresadas tras un parto |
| **FUENTE DE DATOS** | LVNS (si caso)  Cuestionario a madres |
| **ELABORACIÓN DEL INDICADOR** | Propio |
| **REFERENCIAS BIBLIOGRÁFICAS**   - World Health Organization (WHO). WHO - Safe childbirth checklist [Internet]. Available from:   <http://www.who.int/patientsafety/implementation/checklists/childbirth/en/>   - Norma Oficial Mexicana NOM-007-SSA2-1993, Atención de la mujer durante el embarazo, parto y puerperio y del recién nacido. Criterios y procedimientos para la prestación del servicio. | |
| **OBSERVACIONES**  Se orientará a la madre en los cuidados del recién nacido, la lactancia materna exclusiva, sobre métodos de planificación familiar, la alimentación materna y acerca de los cambios emocionales que pueden presentarse durante el postparto. | |

| FICHA TÉCNICA DE INDICADORES: PROCESO | |
| --- | --- |
| **GRUPO:** | Buenas prácticas |
| **AREA:** | Antes del egreso |
| Sub-área | Atención de la madre |
| **Nº** | 2 (de 2) |
| **NOMBRE INDICADOR** | Información sobre los signos de alarma de la madre |
| **FORMA DE MEDICIÓN** | Muestreo de expedientes de mujeres atendidas por motivo de parto;  Realización de encuesta  LVNS |
| **DESCRIPCIÓN** | Porcentaje de mujeres y/o el acompañantes fueron informados sobre los signos de alarma de la madre ante los que pedir ayuda |
| **Nivel de evidencia; Fuerza recomendación** | No aplica |
| **FÓRMULA DEL INDICADOR** | Número de mujeres y/o acompañantes a los que se les informó de los signos de alarma de la madre ante los que debía pedir ayuda al egreso |
| **Numerador** |  |
| **Denominador** | Número de mujeres que fueron atendidas por motivo de parto |
| **FUENTE DE DATOS** | Revisión de la LVNS  Cuestionario a mujer/ acompañante |
| **ELABORACIÓN DEL INDICADOR** | Propio |
| **REFERENCIAS BIBLIOGRÁFICAS**  World Health Organization (WHO). WHO - Safe childbirth checklist [Internet]. Available from:  <http://www.who.int/patientsafety/implementation/checklists/childbirth/en/> | |
| **OBSERVACIONES**  Se valoran los signos de alarma ante los que se debe pedir ayuda al egreso descritos en la LVNS (por cada signo y del conjunto –indicador compuesto tipo 100% de cumplimiento–):   - Sangrado - Dolor abdominal intenso - Dolor de cabeza intenso - Alteración del estado de conciencia - Alteraciones visuales n° de LVNS revisadas - Dificultad respiratoria - Dificultad para vaciar la vejiga - Fiebre y/o escalofríos   Herramienta de contraste de información: encuesta a madres. Si bien, sólo se registrarán los síntomas y signos de los que se acuerden. | |

| FICHA TÉCNICA DE INDICADORES: PROCESO | |
| --- | --- |
| **GRUPO:** | Buenas prácticas |
| **AREA:** | Antes del egreso |
| Sub-área | Atención de la madre y del recién nacido |
| **Nº** | 1 (de 1) |
| **NOMBRE INDICADOR** | Organización y acuerdos sobre el seguimiento de la madre y el recién nacido |
| **FORMA DE MEDICIÓN** | Muestreo de expedientes de mujeres atendidas por motivo de parto;  Realización de encuesta  LVNS |
| **DESCRIPCIÓN** | Porcentaje de mujeres que conocen las visitas que deben hacer (ellas y el recién nacido) y el lugar al que acudir a las revisiones antes de su egreso |
| **Nivel de evidencia; Fuerza recomendación** | No identificado |
| **FÓRMULA DEL INDICADOR** | Número de mujeres que son informadas de antes de su egreso de las visitas que deben hacer (ellas y el recién nacido) |
| **Numerador** |  |
| **Denominador** | Número total de mujeres que egresan después del parto |
| **FUENTE DE DATOS** | LVNS (si caso)  Cuestionario a las madres en el momento del egreso |
| **ELABORACIÓN DEL INDICADOR** | Propio |
| **REFERENCIAS BIBLIOGRÁFICAS**   - World Health Organization (WHO). WHO - Safe childbirth checklist [Internet]. Available from:   <http://www.who.int/patientsafety/implementation/checklists/childbirth/en/>   - Norma Oficial Mexicana NOM-007-SSA2-1993, Atención de la mujer durante el embarazo, parto y puerperio y del recién nacido. Criterios y procedimientos para la prestación del servicio. | |
| **OBSERVACIONES**   - **Madre:**   Puerperio inmediato (2o. al 7o. día) y tardío (8o. a 42o. día): Se deberá proporcionar un mínimo de tres consultas, con una periodicidad que abarque el término de la primera semana (para la primera consulta), y el término del primer mes (para la tercera); el segundo control debe realizarse dentro del periodo entre la primera y la tercera visita, acorde con el estado de salud de la mujer.   - **Recién nacido:**   Visitas de control a los 7 días y a los 28 días.  Para la evaluación de este indicador sólo se tendrá en cuenta que la madre haya sido informada de la primera visita para ella y para su recién nacido.  Método de validación de la LVNS: medición independiente de la LVNS y de los resultados de la encuesta a las madres. | |

| FICHA TÉCNICA DE INDICADORES: PROCESO | |
| --- | --- |
| **GRUPO:** | Buenas prácticas |
| **AREA:** | Antes del egreso |
| Sub-área | Atención del recién nacido |
| **Nº** | 1 (de 1) |
| **NOMBRE INDICADOR** | Información sobre los signos de alarma del recién nacido |
| **FORMA DE MEDICIÓN** | Muestreo de expedientes de mujeres atendidas por motivo de parto;  Realización de encuesta  LVNS |
| **DESCRIPCIÓN** | Porcentaje de mujeres y/o el acompañantes que fueron informados sobre los signos de alarma del recién nacido ante los que regresar a alguna unidad de salud y pedir ayuda |
| **Nivel de evidencia; Fuerza recomendación** | No identificado |
| **FÓRMULA DEL INDICADOR** | Número de mujeres y/o el acompañante a las que se les informó de los signos de alarma del recién nacido |
| **Numerador** |  |
| **Denominador** | Número de recién nacidos que egresan |
| **FUENTE DE DATOS** | Revisión de la LVNS  Cuestionario a mujer / acompañante |
| **ELABORACIÓN DEL INDICADOR** | Propio |
| **REFERENCIAS BIBLIOGRÁFICAS**  World Health Organization (WHO). WHO - Safe childbirth checklist [Internet]. Available from:  <http://www.who.int/patientsafety/implementation/checklists/childbirth/en/> | |
| **OBSERVACIONES**  Se valoran los signos de alarma del recién nacido ante los que se debe pedir ayuda descritos en la LVNS (para cada signo y del conjunto –indicador compuesto tipo 100% de cumplimiento–):   - Fiebre - Respiración rápida o dificultad para respirar - Extremadamente frio - No orina o evacua - Cianosis - Interrupción de la correcta alimentación n° de LVNS revisadas - Crisis convulsivas - Menos actividad de lo normal - Ictericia - Rechazo a vía oral o vómitos   Herramienta de validación de información: encuesta a madres | |

# 4. Indicadores de resultado

| FICHA TÉCNICA DE INDICADORES: RESULTADO | |
| --- | --- |
| **GRUPO:** | Resultados en salud |
| **AREA:** | Complicaciones (morbilidad) |
| Sub-área | Madre |
| **Nº** | 1 (de 3) |
| **NOMBRE INDICADOR** | Incidencia de hemorragia obstétrica |
| **FORMA DE MEDICIÓN** | Muestreo de expedientes de mujeres que fueron atendidas por motivo de parto |
| **DESCRIPCIÓN** | Porcentaje de mujeres sufrieron hemorragia (periodos quincenales) |
| **Nivel de evidencia; Fuerza recomendación** | No aplica |
| **FÓRMULA INDICADOR** | Número de mujeres que presentaron hemorragia intra- y post-parto |
| **Numerador** |  |
| **Denominador** | Número de mujeres que fueron atendidas por motivo de parto |
| **FUENTE DE DATOS** | Historia clínica  LVNS |
| **ELABORACIÓN DEL INDICADOR** | Propio |
| **REFERENCIAS BIBLIOGRÁFICAS**   - Secretaría de Salud. Diagnóstico y tratamiento de la hemorragia obstétrica en la segunda mitad del embarazo y puerperio inmediato. IMSS-162-09. [Internet]. México; 2009. Available from: <http://www.cenetec.salud.gob.mx/interior/catalogoMaestroGPC.html> - Instituto Mexicano de la Seguridad Social (IMSS). Guía de práctica clínica. Detección y tratamiento inicial de las emergencias obstétricas. IMSS-436-11. [Internet]. México; 2011. Available from: <http://www.cenetec.salud.gob.mx/interior/catalogoMaestroGPC.html> | |
| **OBSERVACIONES**   - Hemorragia obstétrica grave: pérdida sanguínea de origen obstétrico, con presencia de alguno de los siguientes criterios: pérdida del 25% de la volemia, caída del hematocrito mayor de 10 puntos, presencia de cambios hemodinámicos o pérdida mayor de 150 ml/min. - Hemorragia postparto (75% de los casos del puerperio patológico): pérdida sanguínea de más de 500 ml posterior a un parto vaginal, o la pérdida de más de 1000 ml posterior a una cesárea. También se ha definido como la disminución del nivel de hematocrito de 10%. Se considera como primaria, si ocurre dentro de las primeras 24 horas posteriores al nacimiento y su causa principal es la atonía uterina (80% de los casos).   Alternativamente, se intentará en el estudio piloto obtener el dato de la estadística mensual que el centro reporta, así como el total de los casos registrados en bases de datos electrónicas con los CIE 10:  O67: Trabajo de parto y parto complicado por hemorragia intraparto, no clasificado en otra parte  O71: Otro traumatismo obstétrico  O72: Hemorragia postparto | |

| FICHA TÉCNICA DE INDICADORES: RESULTADO | |
| --- | --- |
| **GRUPO:** | Resultados en salud |
| **AREA:** | Complicaciones (morbilidad) |
| Sub-área | Madre |
| **Nº** | 2 (de 3) |
| **NOMBRE INDICADOR** | Incidencia de trastornos de la presión arterial pre-, intra- y post-parto |
| **FORMA DE MEDICIÓN** | Muestreo de expedientes de mujeres que fueron atendidas por motivo de parto |
| **DESCRIPCIÓN** | Porcentaje de mujeres con trastornos de la presión arterial en el proceso del parto (periodos quincenales) |
| **Nivel de evidencia; Fuerza recomendación** | No aplica |
| **FÓRMULA INDICADOR** | Número de mujeres con trastornos de la presión arterial pre-, intra- y post-parto |
| **Numerador** |  |
| **Denominador** | Número de mujeres que fueron atendidas por motivo de parto |
| **FUENTE DE DATOS** | Historia clínica - LVNS - Partograma |
| **ELABORACIÓN DEL INDICADOR** | Propio |
| **REFERENCIAS/BIBLIOGRÁFICAS**   - Secretaría de Salud. Detección y Diagnóstico de Enfermedades Hipertensivas del Embarazo. (México D. F., 2010).   <http://www.cenetec.salud.gob.mx/interior/catalogoMaestroGPC.html>   - Secretaría de Salud. Guía de Práctica Clínica Intervenciones de Enfermería en la paciente con Preeclampsia/Eclampsia. (México, 2011).   <http://www.cenetec.salud.gob.mx/interior/catalogoMaestroGPC.html>   - Secretaría de Salud. Detección y tratamiento inicial de las emergencias obstétricas. (México, 2011).   <http://www.cenetec.salud.gob.mx/interior/catalogoMaestroGPC.html> | |
| **OBSERVACIONES**   - Preeclampsia: síndrome multisistémico del embarazo y puerperio. Se presenta después de la semana 20 de la gestación, durante el parto o en las primeras dos semanas después de éste. Hipertensión arterial ≥ 140/90 mmHg y proteinuria, frecuente con cefalea, acúfenos, fosfenos, edema, dolor abdominal y/o alteraciones de laboratorio. - Leve: PAS≥ 140 mmHg y/o PAD ≥ 90 mmHg en mujer normotensa (2 tomas con diferencia de 6 horas entre ellas, y un lapso máximo de 7 días). Proteinuria ≥300mg en recolección de orina de 24 horas o reporte en tira reactiva de por lo menos 30 mg/dl (1+) en 2 muestras de orina tomadas al azar (diferencia de 6 horas entre ellas, lapso máximo de 7 días y sin evidencia de infección de vías urinarias. - Severa: PA≥ 160/110 mmHg en al menos 2 determinaciones con mínimo 6 horas de diferencia. Proteinuria de 5 gr o más en una colección de orina de 24 horas (por tira reactiva 3+ o más en 2 muestras con al menos 4 horas de diferencia). Otras características: oliguria (menos de 500 ml de orina en 24 horas), alteraciones visuales, edema pulmonar, cianosis, dolor epigástrico o en hipocondrio derecho, pruebas de función hepática alteradas (incremento de DHL> 600 UI, elevación al doble de AST y ALT), creatinina sérica >1.2 mg/dl, trombocitopenia (plaquetas < 150 000 cel/mm3,), restricción del crecimiento intrauterino. - Eclampsia: convulsiones o coma en pacientes con preeclampsia después de la semana 20 de gestación, parto o en las primeras 6 semanas después de éste, en ausencia de otras causas de convulsiones. - Síndrome de HELLP (variante atípica de la preeclampsia grave): hemólisis microangiopática, elevación de las enzimas hepáticas y trombocitopenia.   Se intentará en el piloto: Recoger el dato de la estadística mensual que el centro reporta con el total de los casos para contrastar por CIE 10.  O11, O14, O15, O16 | |

| FICHA TÉCNICA DE INDICADORES: RESULTADO | |
| --- | --- |
| **GRUPO:** | Resultados en salud |
| **AREA:** | Complicaciones (morbilidad) |
| Sub-área | Madre |
| **Nº** | 3 (de 3) |
| **NOMBRE INDICADOR** | Incidencia de infección post-parto o perinatal materna |
| **FORMA DE MEDICIÓN** | Muestreo de expedientes de mujeres que fueron atendidas por motivo de parto |
| **DESCRIPCIÓN** | Porcentaje de mujeres con infección perinatal o post-parto (periodos quincenales) |
| **Nivel de evidencia; Fuerza recomendación** | No aplica |
| **FÓRMULA INDICADOR** | Número de mujeres con infección perinatal o post-parto |
| **Numerador** |  |
| **Denominador** | Número de mujeres que fueron atendidas por motivo de parto |
| **FUENTE DE DATOS** | Historia clínica  LVNS |
| **ELABORACIÓN DEL INDICADOR** | Propio |
| **REFERENCIAS BIBLIOGRÁFICAS**  ^1^Organización Mundial de la Salud. Guía de la OMS para la aplicación de la CIE-10 a las muertes ocurridas durante el embarazo, parto y puerperio: CIE MM. Ginebra 2012.ISBN 978 92 4 154845 8 | |
| **OBSERVACIONES**  Se tendrán en cuenta los casos en los que su expediente clínico consten criterios clínicos (consensuados entre los centros participantes) o registro de infección (prescripción de antibiótico y causa) en LVNS como identificación de las unidades del numerador.  **Se intentará en el piloto:** Recoger el dato de la estadística mensual que el centro reporta con el total de los casos para contrastar.  Alternativamente, se utilizarán códigos diagnósticos CIE10 para la identificación de casos, si es posible su explotación en bases de datos electrónicas, y se pedirá el dato de la estadística mensual que el centro reporta.  Códigos CIE 10^1^:  O75.3: Otra infección durante el trabajo de parto  O85: Sepsis puerperal  O86: Otras infecciones puerperales  Si en el piloto se detecta la imposibilidad de obtener los registros de infección, el criterio se sustituirá por “se prescribe antibiótico” | |

| FICHA TÉCNICA DE INDICADORES: RESULTADO | |
| --- | --- |
| **GRUPO:** | Resultados en salud |
| **AREA:** | Complicaciones (morbilidad) |
| Sub-área | Recién nacido |
| **Nº** | 1 (de 2) |
| **NOMBRE INDICADOR** | Incidencia de infección neonatal |
| **FORMA DE MEDICIÓN** | Muestreo de expedientes de mujeres que fueron atendidas por motivo de parto o de expedientes de recién nacidos abiertos |
| **DESCRIPCIÓN** | Porcentaje de neonatos con infección neonatal (periodos quincenales) |
| **Nivel de evidencia; Fuerza recomendación** | No aplica |
| **FÓRMULA INDICADOR** | Número de neonatos con infección |
| **Numerador** |  |
| **Denominador** | Número recién nacido vivos |
| **FUENTE DE DATOS** | Historia clínica del recién nacido  LVNS |
| **ELABORACIÓN DEL INDICADOR** | Propio |
| **REFERENCIAS BIBLIOGRÁFICAS**  No aplica | |
| **OBSERVACIONES**  Mención específica en expediente clínico del diagnóstico de infección o motivo de prescripción de antibiótico  **Se intentará en el piloto:** Recoger el dato de la estadística mensual que el centro reporta con el total de los casos para contrastar.  Alternativamente, se pueden utilizar códigos CIE10 para identificar los casos, si existe registro electrónico que lo permita.  [P36](https://eciemaps.mspsi.es/ecieMaps/browser/index_10_mc.html#search=P36&flags=111100&flagsLT=11111111&searchId=1436825362125&indiceAlfabetico=expand-0a65479&listaTabular=P36&expand=1&clasification=cie10mc&version=2010) – Sepsis bacteriana del recién nacido  P37 - Otras enfermedades infecciosas y parasitarias congénitas  P38 - Onfalitis del recién nacido con o sin hemorragia  [P39](https://eciemaps.mspsi.es/ecieMaps/browser/index_10_mc.html#search=P39&flags=111100&flagsLT=11111111&searchId=1436825484645&indiceAlfabetico=expand-0a65479a65670&listaTabular=P39&expand=1&clasification=cie10mc&version=2010) – Otras infecciones específicas del período perinatal  Si en el piloto se detecta la imposibilidad de obtener los registros de infección, el criterio se sustituirá por “se prescribe antibiótico” | |

| FICHA TÉCNICA DE INDICADORES: RESULTADO | |
| --- | --- |
| **GRUPO:** | Resultados en salud |
| **AREA:** | Complicaciones (morbilidad) |
| Sub-área | Recién nacido |
| **Nº** | 2 (de 2) |
| **NOMBRE INDICADOR** | Incidencia de asfixia neonatal |
| **FORMA DE MEDICIÓN** | Muestreo de expedientes de mujeres que fueron atendidas por motivo de parto o de expedientes de recién nacidos abiertos |
| **DESCRIPCIÓN** | Porcentaje de neonatos con asfixia neonatal (periodos quincenales) |
| **Nivel de evidencia; Fuerza recomendación** | No aplica |
| **FÓRMULA INDICADOR** | Número de neonatos con evento de asfixia neonatal |
| **Numerador** |  |
| **Denominador** | Número recién nacido vivos |
| **FUENTE DE DATOS** | Historia clínica del recién nacido |
| **ELABORACIÓN DEL INDICADOR** | Propio |
| **REFERENCIAS BIBLIOGRÁFICAS**  No aplica | |
| **OBSERVACIONES**  Mención específica de la asfixia neonatal en el expediente clínico  Recoger el dato de la estadística mensual que el centro reporta con el total de los casos para contrastar.  [P20](http://apps.who.int/classifications/icd10/browse/2015/en#/P20) – Hipoxia intrauterina (descartar otros diagnósticos)  [P21](http://apps.who.int/classifications/icd10/browse/2015/en#/P21) – Asfixia en el parto | |

| FICHA TÉCNICA DE INDICADORES: RESULTADO | |
| --- | --- |
| **GRUPO:** | Resultados en salud |
| **AREA:** | Intervenciones |
| Sub-área |  |
| **Nº** | 1 (de 3) |
| **NOMBRE INDICADOR** | Porcentaje de partos con cesárea |
| **FORMA DE MEDICIÓN** | Muestreo de expedientes de mujeres que fueron atendidas por motivo de parto |
| **DESCRIPCIÓN** | Porcentaje de mujeres con resolución del parto por cesárea |
| **Nivel de evidencia; Fuerza recomendación** | No aplica |
| **FÓRMULA INDICADOR** | Número de mujeres a las que se les realiza cesárea |
| **Numerador** |  |
| **Denominador** | Número de mujeres que fueron atendidas por motivo de parto |
| **FUENTE DE DATOS** | Expedientes clínicos  LVNS (si es caso) |
| **ELABORACIÓN DEL INDICADOR** | Propio |
| **REFERENCIAS BIBLIOGRÁFICAS**  Norma Oficial Mexicana NOM-007-SSA2-1993, Atención de la mujer durante el embarazo, parto y puerperio y del recién nacido. Criterios y procedimientos para la prestación del servicio. | |
| **OBSERVACIONES**  Toda unidad médica con atención obstétrica debe contar con lineamientos para la indicación de cesárea, cuyo índice idealmente se recomienda de 15% en los hospitales de segundo nivel y del 20% en los del tercer nivel en relación con el total de nacimientos, por lo que las unidades de atención médica deben aproximarse a estos valores. | |

| FICHA TÉCNICA DE INDICADORES: RESULTADO | |
| --- | --- |
| **GRUPO:** | Resultados en salud |
| **AREA:** | Intervenciones |
| Sub-área |  |
| **Nº** | 2 (de 3) |
| **NOMBRE INDICADOR** | Porcentaje de partos instrumentados |
| **FORMA DE MEDICIÓN** | Muestreo de expedientes de mujeres que fueron atendidas por motivo de parto |
| **DESCRIPCIÓN** | Porcentaje de mujeres con resolución del parto instrumentado |
| **Nivel de evidencia; Fuerza recomendación** | No aplica |
| **FÓRMULA INDICADOR** | Número de mujeres a las que se les realiza un parto instrumentado |
| **Numerador** |  |
| **Denominador** | Número de mujeres que fueron atendidas por motivo de parto |
| **FUENTE DE DATOS** | Expedientes clínicos  LVNS (si es caso) |
| **ELABORACIÓN DEL INDICADOR** | Propio |
| **REFERENCIAS BIBLIOGRÁFICAS**  Guía de Práctica Clínica para la Reducción de la Frecuencia de Operación Cesárea México: Instituto Mexicano de Seguro social; 2014. ISBN: 978-607-7790-92-1 | |
| **OBSERVACIONES** | |

| FICHA TÉCNICA DE INDICADORES: RESULTADO | |
| --- | --- |
| **GRUPO:** | Resultados en salud |
| **AREA:** | Intervenciones |
| Sub-área |  |
| **Nº** | 3 (de 3) |
| **NOMBRE INDICADOR** | Porcentaje de partos con episiotomía |
| **FORMA DE MEDICIÓN** | Muestreo de expedientes de mujeres que fueron atendidas por motivo de parto |
| **DESCRIPCIÓN** | Porcentaje de mujeres con episiotomía en el parto |
| **Nivel de evidencia; Fuerza recomendación** | No aplica |
| **FÓRMULA INDICADOR** | Número de mujeres con episiotomía en el parto |
| **Numerador** |  |
| **Denominador** | Número de mujeres que fueron atendidas por motivo de parto |
| **FUENTE DE DATOS** | Expedientes clínicos  LVNS (si es caso) |
| **ELABORACIÓN DEL INDICADOR** | Propio |
| **REFERENCIAS BIBLIOGRÁFICAS**   - - - 1. Informe presentado por el Grupo Técnico de Trabajo - OMS. CUIDADOS EN EL PARTO NORMAL: UNA GUÍA PRÁCTICA. Ginebra; 1996.       2. Belizan J, Campodonico L, Carroli G, Gonzalez L. **Routine vs selective episiotomy: a randomised controlled trial**. *Lancet*. 1993; **18**:1517-8.       3. Hartmann K, Viswanathan M, Palmieri R, Gartlehner G, Thorp J, Lohr KN. **Outcomes of routine episiotomy: a systematic review**. *JAMA*. 2005; **17**:2141-8. | |
| **OBSERVACIONES**  En el informe de 1996 de la OMS se menciona que tras un estudio (Sleep et al 1984), un buen objetivo podría ser una frecuencia de episiotomía no superior al 10%^1^ para los partos normales. Posteriormente, diferentes autores han recomendado que la realización de episiotomía debería estar entre el 30% y el 15%^2,3^, pero en ningún caso debería superar el 30%^2^. | |

| FICHA TÉCNICA DE INDICADORES: RESULTADO | |
| --- | --- |
| **GRUPO:** | Resultados en salud |
| **AREA:** | Eventos adversos |
| Sub-área | Madre |
| **Nº** | 1 (de 1) |
| **NOMBRE INDICADOR** | Eventos adversos en la madre |
| **FORMA DE MEDICIÓN** | Muestreo de expedientes de mujeres que fueron atendidas por motivo de parto |
| **DESCRIPCIÓN** | Porcentaje de eventos adversos en mujeres que han sido atendidas por parto |
| **Nivel de evidencia; Fuerza recomendación** | No aplica |
| **FÓRMULA INDICADOR** | Número de mujeres con, al menos, un evento adverso |
| **Numerador** |  |
| **Denominador** | Total de partos |
| **FUENTE DE DATOS** | Expediente clínico |
| **ELABORACIÓN DEL INDICADOR** | Adaptado de Pettker et al. |
| **REFERENCIAS BIBLIOGRÁFICAS**  Pettker CM et al. Impact of a comprehensive patient safety strategy on obstetric adverse events. Am Journal Obst Gynecol, 2009, 200:492e1-492e8. Disponible en: http://www.ajog.org/article/S0002-9378(09)00092-1/pdf. | |
| **OBSERVACIONES**  Se tendrán en cuenta todos los eventos adversos listados, pero sólo se considera un evento adverso por mujer:   - Transfusión sanguínea - Laceración de 3° o 4° grado - Internadas en UCI - Histerectomia post-parto - Ruptura uterina - Regreso al hospital después del egreso - Muerte materna   La utilización de un indicador compuesto facilita la obtención de un resultado de eventos adversos, minimizando la baja frecuencia de alguno de ellos, y señalando individualmente la gravedad de los casos, ya que el más alto es el numerador del indicador de eventos adversos probablemente más fallos en el servicio existía el parto. | |

| FICHA TÉCNICA DE INDICADORES: RESULTADO | |
| --- | --- |
| **GRUPO:** | Resultados en salud |
| **AREA:** | Morbi-mortalidad |
| Sub-área | Recién nacido |
| **Nº** | 1 (de 1) |
| **NOMBRE INDICADOR** | Eventos adversos en recién nacidos |
| **FORMA DE MEDICIÓN** | Muestreo de expedientes de mujeres que fueron atendidas por motivo de parto o de los recién nacido |
| **DESCRIPCIÓN** | Porcentaje de eventos adversos en recién nacidos que han sido atendidos en el centro |
| **Nivel de evidencia; Fuerza recomendación** | No aplica |
| **FÓRMULA INDICADOR** | Número de recién nacidos con, al menos, un efecto adverso |
| **Numerador** |  |
| **Denominador** | Total de nacimientos |
| **FUENTE DE DATOS** | Expediente clínico |
| **ELABORACIÓN DEL INDICADOR** | Adaptado de Pettker et al. |
| **REFERENCIAS BIBLIOGRÁFICAS**  Pettker CM et al. Impact of a comprehensive patient safety strategy on obstetric adverse events. Am Journal Obst Gynecol, 2009, 200:492e1-492e8. Disponible en: http://www.ajog.org/article/S0002-9378(09)00092-1/pdf. | |
| **OBSERVACIONES**  Se tendrán en cuenta todos los eventos adversos listados, pero sólo se considera un evento adverso por recién nacido:   - Internados en UCIN con peso > 2500g e por >24h; - APGAR < 7 al minuto 5 - Trauma o herida en el parto (p. ej: traumatismo craneano, fractura, herida neurológica, hemorragia o laceración); - Hospitalizado más de 7 días - Muerte fetal o neonatal   La utilización de un indicador compuesto facilita la obtención de un resultado de eventos adversos, minimizando la baja frecuencia de alguno de ellos, y señalando individualmente la gravedad de los casos, ya que el más alto es el numerador del indicador de eventos adversos probablemente más fallos en el servicio existía el parto. | |
