# Supplementary material for: Construction and pilot test of a set of indicators to assess the implementation and effectiveness of the who safe childbirth checklist
Source: BMC Pregnancy Childbirth. 2018 May 10;18:154. doi: 10.1186/s12884-018-1797-y (PMC5946578; doi:10.1186/s12884-018-1797-y)
Supplement: Supplementary file 4 — Set of SCC indicators (english version). The detailed definitions of the indicators in English. (DOCX 139 kb) [file 12884_2018_1797_MOESM4_ESM.docx]

**SET OF INDICATORS FOR THE**

**SAFE CHILDBIRTH CHECKLIST (SCC)**

[1. Indicators for implementation 2](#_Toc510078495)

[1.2. Training on SCC 2](#_Toc510078496)

[1.2. Characteristics of the team 4](#_Toc510078497)

[1.3. Attitude of health professionals 9](#_Toc510078498)

[1.4. SCC Availability 12](#_Toc510078499)

[2. SCC utilization 13](#_Toc510078500)

[3. Good practice indicators 17](#_Toc510078501)

[3.1. General 17](#_Toc510078502)

[3.2. Indicators for phase of admission 24](#_Toc510078503)

[3.3. Indicators for pre-expulsive phase (or before cesarean section) 28](#_Toc510078504)

[3.4. Indicators for immediate postpartum phase (first hour after expulsion) 33](#_Toc510078505)

[3.5. Indicators for phase just before discharge 40](#_Toc510078506)

[4. Outcome indicators 44](#_Toc510078507)

# **Indicators for implementation**

## 1.2. Training on SCC

| TECHNICAL DATE OF INDICATORS: structure | |
| --- | --- |
| **GROUP:** | Factors potentially associated to SCC implementation |
| **AREA:** | Health professionals |
| Sub-area | Training |
| **Nº** | 1 (of 2) |
| **INDICATOR NAME** | Professionals trained in the use of the SCC |
| **FORM OF MEASUREMENT** | Survey of professionals and training records |
| **DESCRIPTION** | Percentage of professionals who received training in the use of SCC |
| **Level of evidence; Force recommendation** | Not applicable |
| **INDICATOR FORMULA** | Number of health professionals who have received training in the use of the SCC |
| **Numerator** |  |
| **Denominator** | Total professionals of the emergency services, surgery, obstetrics and neonatal services involved in the process of attending childbirth |
| **DATA SOURCE** | Training records/Questionnaire to health professionals |
| **INDICATOR ELABORATION** | Self-developed |
| **BIBLIOGRAPHIC REFERENCES**  Not applicable | |
| **OBSERVATIONS**  It is suggested to obtain the total percentage for the center, and the disaggregated by service and per shift. | |

| TECHNICAL DATE OF INDICATORS: structure | |
| --- | --- |
| **GROUP:** | Factors potentially associated to SCC implementation |
| **AREA:** | Health professionals |
| Sub-area | Training |
| **Nº** | 2 (of 2) |
| **INDICATOR NAME** | Professionals trained in the use of the SCC by professional profile |
| **FORM OF MEASUREMENT** | Survey of professionals and training records |
| **DESCRIPTION** | Percentage of professionals who received training in the use of SCC according to professional profile |
| **Level of evidence; Force recommendation** | Not applicable |
| **INDICATOR FORMULA** | Number of professionals per respondent profile who have received training in the use of the SCC |
| **Numerator** |  |
| **Denominator** | Total of professionals by profile |
| **DATA SOURCE** | Training records/Questionnaire to health professionals |
| **INDICATOR ELABORATION** | Individual |
| **BIBLIOGRAPHIC REFERENCES**  Not applicable | |
| **OBSERVATIONS**   - Disaggregate results for the following professional profiles: - General Nurse - Obstetric nurse - Doula - Psychologist - General Doctor - Gynecologist / obstetrician - Neonatologist / pediatrician - Resident doctor | |

## 1.2. Characteristics of the team

| TECHNICAL DATE OF INDICATORS: structure | |
| --- | --- |
| **GROUP:** | Factors potentially associated to SCC implementation |
| **AREA:** | Health professionals |
| Sub-area | Team |
| **Nº** | 1 (of 5) |
| **INDICATOR NAME** | Team size (analysis variable) |
| **FORM OF MEASUREMENT** | Audit |
| **DESCRIPTION** | Reason for professionals in relation to the beds of the services of gynecology-obstetrics and neonatology |
| **Level of evidence; Force recommendation** | Unidentified |
| **INDICATOR FORMULA** | Number of team professionals |
| **Numerator** |  |
| **Denominator** | Beds of the gynecology-obstetrics and neonatology service |
| **DATA SOURCE** | Log books/Questionnaire to health professionals |
| **INDICATOR ELABORATION** | Individual |
| **BIBLIOGRAPHIC REFERENCES**  Not applicable | |
| **OBSERVATIONS**  Disaggregate by professional profile, per service and per shift, always based on the number of beds available for delivery care in the gynecology-obstetrics and neonatology service. Optionally, the reason for the number of beds in the care center can also be calculated.  Shifts: morning, afternoon, evening, weekend and holiday  Team Profiles:   - General Nurse - Obstetric nurse - Doula - Psychologist - General Doctor - Gynecologist / obstetrician - Neonatologist / pediatrician - Resident doctor | |

| TECHNICAL DATE OF INDICATORS: structure | |
| --- | --- |
| **GROUP:** | Factors potentially associated to SCC implementation |
| **AREA:** | Health professionals |
| Sub-area | Team |
| **Nº** | 2 (of 5) |
| **INDICATOR NAME** | Professional profile of team members (analysis variable) |
| **FORM OF MEASUREMENT** | Audit |
| **DESCRIPTION** | Volume of each professional profile of the work team (service and shift) |
| **Level of evidence; Force recommendation** | Unidentified |
| **INDICATOR FORMULA** | Number of professionals in each professional profile per service and shift |
| **Numerator** |  |
| **Denominator** | Not applicable |
| **DATA SOURCE** | Log books/Questionnaire to health professionals |
| **INDICATOR ELABORATION** | Individual |
| **BIBLIOGRAPHIC REFERENCES**  Unidentified | |
| **OBSERVATIONS**  Services: emergency, surgery and gynecology-obstetrics  Shifts: morning, afternoon, evening, weekend and holiday  Team Profiles:   - General Nurse - Obstetric nurse - Doula - Psychologist - General Doctor - Gynecologist / obstetrician - Neonatologist / pediatrician - Resident doctor | |

| TECHNICAL DATE OF INDICATORS: structure | |
| --- | --- |
| **GROUP:** | Factors potentially associated to SCC implementation |
| **AREA:** | Health professionals |
| Sub-area | Team |
| **Nº** | 3 (of 5) |
| **INDICATOR NAME** | Availability of staff trained in neonatal resuscitation (in the service) |
| **FORM OF MEASUREMENT** | Audit; Poll |
| **DESCRIPTION** | Existence of personnel with updated training in neonatal resuscitation in each service and each shift |
| **Level of evidence; Force recommendation** | Not applicable |
| **INDICATOR FORMULA** | Existence of professionals with updated training in neonatal resuscitation in each service and each shift |
| **Numerator** |  |
| **Denominator** | Not applicable |
| **DATA SOURCE** | Questionnaire to health professionals |
| **INDICATOR ELABORATION** | Individual |
| **BIBLIOGRAPHIC REFERENCES**  Not applicable | |
| **OBSERVATIONS**  Up-to-date training is understood to be in force (carried out within a period of less than 2 years).  Services: emergency, surgery and gynecology-obstetrics  Shifts: morning, afternoon, evening, weekend and holiday | |

| TECHNICAL DATE OF INDICATORS: structure | |
| --- | --- |
| **GROUP:** | Factors potentially associated to SCC implementation |
| **AREA:** | Health professionals |
| Sub-area | Team |
| **Nº** | 4 (of 5) |
| **INDICATOR NAME** | Composition of the team that attends the childbirth (pre-expulsive stage) |
| **FORM OF MEASUREMENT** | Observation |
| **DESCRIPTION** | Profile of the team that attended each childbirth |
| **Level of evidence; Force recommendation** | Not applicable |
| **INDICATOR FORMULA** | Typology of equipment composition present at the time of childbirth (for each childbirth) |
| **Numerator** |  |
| **Denominator** | Not applicable |
| **DATA SOURCE** | Log books/Observation/Questionnaire to health professionals |
| **INDICATOR ELABORATION** | Individual |
| **BIBLIOGRAPHIC REFERENCES**  Not applicable | |
| **OBSERVATIONS**  Existing professional profiles should be recorded for each delivery as well as the existence of the professional with training in neonatal resuscitation (see below).  The number of professionals present at delivery for each profile is independently reported. It will be assessed independently each shift.  Shifts: morning, afternoon, evening, weekend and holiday  Team Profiles:  a) General nurse  b) Obstetric nurse  c) doula  d) Psychologist  e) General practitioner  f) Obstetrician / obstetrician  g) Resident physician  h) Neonatologist / pediatrician  i) Any category from "a" to "g" with training in neonatal resuscitation  NOTE: For identification of professionals with training, those who have this training must have been previously identified. In the case of the neonatologist, this training will be considered directly. | |

| TECHNICAL DATE OF INDICATORS: structure | |
| --- | --- |
| **GROUP:** | Factors potentially associated to SCC implementation |
| **AREA:** | Health professionals |
| Sub-area | Team |
| **Nº** | 5 (of 5) |
| **INDICATOR NAME** | Presence of personnel skilled in neonatal resuscitation at birth |
| **FORM OF MEASUREMENT** | Observation; review of clinical files |
| **DESCRIPTION** | Percentage of births with the presence of a professional with updated skilled in neonatal resuscitation |
| **Level of evidence; Force recommendation** | Expert Advice |
| **INDICATOR FORMULA** | Presence of professionals with up-to-date training in neonatal resuscitation at childbith |
| **Numerator** |  |
| **Denominator** | Number of childbirths attended |
| **DATA SOURCE** | Log books/Observation/Questionnaire to health professionals/SCC |
| **INDICATOR ELABORATION** | Individual |
| **BIBLIOGRAPHIC REFERENCES**  ^1^ Asociación Española de Pediatría y Sociedad Española de Neonatología. Protocolos Diagnóstico Terapéuticos de la AEP: Neonatología. 2ª edición. 2008. p. 111-125. <http://www.aeped.es/documentos/protocolos-neonatologia>  ^2^ OMS-Departamento de Investigación y Salud Reproductiva. Cuidados en el parto normal: una guía práctica. Informe presentado por el Grupo Técnico de Trabajo. Ginebra. 1996. | |
| **OBSERVATIONS**  1 At every chilbirth there must be at least one person responsible for the care of the newborn with training in initial neonatal resuscitation.  2 The person attending the childbirth should be able to perform essential basic interventions and care for the child after birth.  Up-to-date training in neonatal resuscitation is understood as an "approved / certified" course conducted or updated within a period of less than 2 years.  NOTE: For the evaluation of this indicator, those who have this training should have been previously identified. In the case of the neonatologist, this training will be considered directly. | |

## 1.3. Attitude of health professionals

| TECHNICAL DATE OF INDICATORS: structure | |
| --- | --- |
| **GROUP:** | Factors potentially associated to SCC implementation |
| **AREA:** | Health professionals |
| Sub-area | Attitude |
| **Nº** | 1 (of 3) |
| **INDICATOR NAME** | Perception of the usefulness of the SCC |
| **FORM OF MEASUREMENT** | Poll |
| **DESCRIPTION** | Percentage of professionals who believe that using SCC is not a waste of time |
| **Level of evidence; Force recommendation** | Not applicable |
| **INDICATOR FORMULA** | Number of professionals who think that using SCC is "never" a waste of time |
| **Numerator** |  |
| **Denominator** | Number of professionals who answered the questionnaire of each service |
| **DATA SOURCE** | Questionnaire to health professionals |
| **INDICATOR ELABORATION** | Individual |
| **BIBLIOGRAPHIC REFERENCES**   - Saturno, P. J., Soria-Aledo, V., Da Silva Gama, Z. a., Lorca-Parra, F., & Grau-Polan, M. (2014). Understanding WHO surgical checklist implementation: Tricks and pitfalls. An observational study. *World Journal of Surgery*, *38*(2), 287–295. doi:10.1007/s00268-013-2300-6 | |
| **OBSERVATIONS**  Each professional profile will be independently assessed, in turn.  Possible survey responses to the question: In your opinion, is the checklist a waste of time in the labor process?  Categories:  1. N ° answers "always"  2. N ° responses "almost always"  3. N ° responses "sometimes"  4. N ° responses "almost never"  5. N ° "never" answers  As a result of this indicator the proportion of responses to the rubric "never" will be obtained. | |

| TECHNICAL DATE OF INDICATORS: structure | |
| --- | --- |
| **GROUP:** | Factors potentially associated to SCC implementation |
| **AREA:** | Health professionals |
| Sub-area | Attitude |
| **Nº** | 2 (of 3) |
| **INDICATOR NAME** | Perception of the impact of the SCC |
| **FORM OF MEASUREMENT** | Poll |
| **DESCRIPTION** | Percentage of professionals who believe that the use of SCC improves the safety of delivery care |
| **Level of evidence; Force recommendation** | Not applicable |
| **INDICATOR FORMULA** | Number of professionals who believe that using SCC "always" improves childbirth safety |
| **Numerator** |  |
| **Denominator** | Number of professionals who answered the questionnaire of each service |
| **DATA SOURCE** | Questionnaire to health professionals |
| **INDICATOR ELABORATION** | Individual |
| **BIBLIOGRAPHIC REFERENCES**   - Saturno, P. J., Soria-Aledo, V., Da Silva Gama, Z. a., Lorca-Parra, F., & Grau-Polan, M. (2014). Understanding WHO surgical checklist implementation: Tricks and pitfalls. An observational study. *World Journal of Surgery*, *38*(2), 287–295. doi:10.1007/s00268-013-2300-6 | |
| **OBSERVATIONS**  Each professional profile will be evaluated independently by turns.  Possible survey responses to the question "Do you think the checklist improves the safety of childbirth care?":  Categories:  1. N ° answers "always"  2. N ° responses "almost always"  3. N ° responses "sometimes"  4. N ° responses "almost never"  5. N ° "never" answers  As a result of this indicator, the proportion of responses to the heading "always" will be obtained. | |

| TECHNICAL DATE OF INDICATORS: structure | |
| --- | --- |
| **GROUP:** | Factors potentially associated to SCC implementation |
| **AREA:** | Health professionals |
| Sub-area | Attitude |
| **Nº** | 3 (of 3) |
| **INDICATOR NAME** | Perception of the importance of the SCC implementation for the hospital |
| **FORM OF MEASUREMENT** | Poll |
| **DESCRIPTION** | Percentage of professionals who perceive the importance of the SCC implementation for the hospital |
| **Level of evidence; Force recommendation** | Not applicable |
| **INDICATOR FORMULA** | Number of professionals professionals who perceive the importance of the SCC implementation for the hospital |
| **Numerator** |  |
| **Denominator** | Number of professionals who answered the questionnaire of each service |
| **DATA SOURCE** | Questionnaire to health professionals |
| **INDICATOR ELABORATION** | Individual |
| **BIBLIOGRAPHIC REFERENCES**  Not applicable | |
| **OBSERVATIONS**  Each professional category will be assessed independently by turns.  Possible survey responses to the question "Do you think your hospital unit considers the implementation of the safe childbirth checklist important?"  or if  or not  or does not know / does not answer  Only responses marked "Yes" will be considered. | |

## 1.4. SCC Availability

| TECHNICAL DATE OF INDICATORS: structure | |
| --- | --- |
| **GROUP:** | Factors potentially associated to SCC implementation |
| **AREA:** | Infrastructure |
| Sub-area | Availability of SCC |
| **Nº** | 1 (of 1) |
| **INDICATOR NAME** | Availability of the SCC formats when needed |
| **FORM OF MEASUREMENT** | Professional Survey |
| **DESCRIPTION** | Percentage of professionals who find the safe childbirth checklist format available when they need it |
| **Level of evidence; Force recommendation** | Not applicable |
| **INDICATOR FORMULA** | Number of professionals per service who respond that they "always" find the SCC format available when they need it |
| **Numerator** |  |
| **Denominator** | Total of professionals who answered the questionnaire for each service and shift |
| **DATA SOURCE** | Questionnaire to health professionals |
| **INDICATOR ELABORATION** | Individual |
| **BIBLIOGRAPHIC REFERENCES**  Not applicable | |
| **OBSERVATIONS**  Proportions will be obtained for each of the frequency responses to the question "In your experience, how often do you find the safe childbirth checklist format available when you need it?":  1. No "always" answers / total service professionals.  2. N ° "almost always" answers / total of service professionals.  3. N ° "sometimes" responses / total of service professionals.  4. No "almost never" answers / total service professionals.  5. "Never" answers / total number of service professionals.  As a result of this indicator, the proportion of responses to the heading "always" will be obtained.  Disaggregated by service and shift respondent. | |

# SCC utilization

| TECHNICAL DATE OF INDICATORS: Process | |
| --- | --- |
| **GROUP:** | SCC utilization |
| **AREA:** |  |
| Sub-area |  |
| **Nº** | 1 (of 4) |
| **INDICATOR NAME** | Use of the SCC format in the hospital (presence in the file) |
| **FORM OF MEASUREMENT** | Sampling of records of women who were attended during labor: Review of filling the SCC |
| **DESCRIPTION** | Percentage of deliveries with SCC (presence in the medical record) |
| **Level of evidence; Force recommendation** | Not applicable |
| **INDICATOR FORMULA** | Number of clinical records with safe childbirth checklist used |
| **Numerator** |  |
| **Denominator** | Total number of childbiths attended |
| **DATA SOURCE** | Medical records |
| **INDICATOR ELABORATION** | Individual |
| **BIBLIOGRAPHIC REFERENCES**  Not applicable | |
| **OBSERVATIONS**  Example for frequency of data collection every 15 days:  In 15 days there have been 130 births  In the same 15 days SCC (initiated) in 90 deliveries  90/130 = 0.69  SCC has been used in 69% of deliveries that have been attended in the last 15 days (the percentage of SCC use has been 69%)  This indicator makes it possible to compare the information with the questionnaire to professionals: "In the deliveries you attend, how often do you use the SCC format?" | |

| TECHNICAL DATE OF INDICATORS: Process | |
| --- | --- |
| **GROUP:** | SCC utilization |
| **AREA:** |  |
| Sub-area |  |
| **Nº** | 2 (of 4) |
| **INDICATOR NAME** | Used of the SCC format in the hospital (complete SCC) |
| **FORM OF MEASUREMENT** | Sampling of records of women who were cared for during delivery: Review of filling the SCC |
| **DESCRIPTION** | Percentage of deliveries with completed SCC (all items) |
| **Level of evidence; Force recommendation** | Not applicable |
| **INDICATOR FORMULA** | Number of SCC completed |
| **Numerator** |  |
| **Denominator** | Number of SCC formats used |
| **DATA SOURCE** | SCC present in medical records |
| **INDICATOR ELABORATION** | Individual |
| **BIBLIOGRAPHIC REFERENCES**  Not applicable | |
| **OBSERVATIONS**  There must be concordance with the moment of entry of the woman to the center. Thus, if a patient enters the hospital having skipped a checklist stage, this stage will not be taken into account for the attribution of compliance / noncompliance.  Only the phases in which the pregnant woman has arrived and the subsequent ones will be taken into account. | |

| TECHNICAL DATE OF INDICATORS: Process | |
| --- | --- |
| **GROUP:** | SCC utilization |
| **AREA:** |  |
| Sub-area |  |
| **Nº** | 3 (of 4) |
| **INDICATOR NAME** | SCC completed items (global) |
| **FORM OF MEASUREMENT** | Sampling of records of women who were attended due to childbirth: Review of filling of SCC. |
| **DESCRIPTION** | Average percentage of SCC completed items (global) |
| **Level of evidence; Force recommendation** | Not applicable |
| **INDICATOR FORMULA** | Not applicable |
| **Numerator** |  |
| **Denominator** | Not applicable |
| **DATA SOURCE** | SCC present in medical records |
| **INDICATOR ELABORATION** | Individual |
| **BIBLIOGRAPHIC REFERENCES**  Not applicable | |
| **OBSERVATIONS**  Weighted mean, based on the overall percentage of each SCC used in the center, depending on the phases applied to each case.  This indicator will be calculated from the indicator "Completed items of the SCC at each stage". | |

| TECHNICAL DATE OF INDICATORS: Process | |
| --- | --- |
| **GROUP:** | SCC utilization |
| **AREA:** |  |
| Sub-area |  |
| **Nº** | 4 (of 4) |
| **INDICATOR NAME** | Completed SCC items by childbirth stage. |
| **FORM OF MEASUREMENT** | Sampling of records of women who were attended due to childbirth: Review of filling of SCC. |
| **DESCRIPTION** | Average percentage of SCC completed items by childbirth stage. |
| **Level of evidence; Force recommendation** | Not applicable |
| **INDICATOR FORMULA** | Number of items in the SCC filled by each stage |
| **Numerator** |  |
| **Denominator** | Number of items per stage (*) of all SCC used |
| **DATA SOURCE** | SCC present in medical records |
| **INDICATOR ELABORATION** | Individual |
| **BIBLIOGRAPHIC REFERENCES**  Not applicable | |
| **OBSERVATIONS**  (*) You will get a percentage for each stage.  It is necessary to verify in the clinical file the phase in which the parturient arrived to the care center. Only the stage at which the attendance began and the subsequent ones will be taken into account. Any stage prior to these will be excluded from the evaluation.  The results of this indicator are disaggregated by SCC items for the mother and the child.  Number of items per phase:  Admission: 12 mother  Before bidding: 10 (8 mother and 2 newborn)  Immediate postpartum: 18 (6 mother and 12 newborn)  Before discharge: 11 (6 mother and 5 newborn) | |

# 3. Good practice indicators

## 3.1. General

| TECHNICAL DATE OF INDICATORS: PROCESS | |
| --- | --- |
| **GROUP:** | Good practices |
| **AREA:** | General |
| Sub-area | Use of antibiotics |
| **Nº** | 1 (of 4) |
| **INDICATOR NAME** | Prescription of antibiotics during labor |
| **FORM OF MEASUREMENT** | Sampling of files of women who were attended for reasons of childbirth |
| **DESCRIPTION** | Percentage of women with antibiotic prescribed during the childbirth process |
| **Level of evidence; Force recommendation** | Not applicable |
| **INDICATOR FORMULA** | Number of women during the labor process with antibiotic prescribed |
| **Numerator** |  |
| **Denominator** | Number of women attended due to childbirth |
| **DATA SOURCE** | Clinical records  SCC (if applicable) |
| **INDICATOR ELABORATION** | Individual |
| **BIBLIOGRAPHIC REFERENCES**   - World Health Organization (WHO). WHO - Safe childbirth checklist [Internet]. Available from: <http://www.who.int/patientsafety/implementation/checklists/childbirth/en/> | |
| **OBSERVATIONS**  Antibiotic prescription:  Any indication or prescription of antibiotic in the clinical file during the entire process of delivery (from admission to discharge) will be valid. The phase of the care process will be captured (admission, pre-expulsion, immediate postpartum or before discharge) in which the indication or prescription is made, although the indicator is reported for the global process of childbirth care.  For purposes of validation of the SCC, the marking can be checked regardless of the cause justifying it, either for the overall process or for the antibiotic start (prescription) phase. | |

| TECHNICAL DATE OF INDICATORS: PROCESS | | | |  |
| --- | --- | --- | --- | --- |
| **GROUP:** | | Good practices | |  |
| **AREA:** | | General | |  |
| Sub-area | | Use of antibiotics | |  |
| **Nº** | | 2 (of 4) | |  |
| **INDICATOR NAME** | Management of antibiotics during labor | | | |
| **FORM OF MEASUREMENT** | Sampling of files of women who were attended for reasons of delivery and prescribed antibiotics | | | |
| **DESCRIPTION** | Percentage of women with antibiotic prescribed during childbirth process antibiotic and it is justified by any symptom | | | |
| **Level of evidence; Force recommendation** | II-2 - Kaimal A, 20081  B - Tita AT, 20081 | | | |
| **INDICATOR FORMULA** | Number of women with antibiotic prescribed during childbirth process antibiotic and it is justified by any symptom | | | |
| **Numerator** |  |  |  |  |
| **Denominator** | Number of women during the labor process with antibiotic prescribed | | | |
| **DATA SOURCE** | Clinical records  SCC (if applicable) | | | |
| **INDICATOR ELABORATION** | Individual | | | |
| **BIBLIOGRAPHIC REFERENCES**   - World Health Organization (WHO). WHO - Safe childbirth checklist [Internet]. Available from:   <http://www.who.int/patientsafety/implementation/checklists/childbirth/en/>   - - - 1. Guía de Práctica Clínica para la Reducción de la Frecuencia de Operación Cesárea México: Instituto Mexicano de Seguro social; 2014. ISBN: 978-607-7790-92-11   2. Organización Panamericana de la Salud “AIEPI Neonatal Intervenciones basadas en evidencia”. Segunda edición. Washington, D.C. 2010 ISBN: 978-92-75-33135-4 | | | | |
| **OBSERVATIONS**  Symptoms that may justify antibiotic prescription are dependent on the delivery phase:  Admission stage: need for antibiotic if patient presents ...  - Temperature> 38 °  - Flow with unpleasant odor (fetid)  - Membrane rupture> 18 hours  - In labor> 24 hours  **Pre-expulsive stage:** need for antibiotic if the patient presents ...  - Temperature> 38 °  - Flow with unpleasant odor (fetid)  - Membrane rupture> 18 hours  - In labor> 24 hours  - Onset of cesarean section1, 2  **Postpartum stage (<1 hour):** need for antibiotic ...  - whether the placenta was removed manually  - if there is a temperature> 38 ° and any of the following: chills and / or discharge with an unpleasant odor (fetid)  **Pre-discharge stage:** need for antibiotic if patient presents ...  - Temperature> 38 ° C and chill or discharge with an unpleasant odor  - Temperature> 38 ° C and low abdominal tone / bloating  In the case of SCC, it can be considered that there is a symptom that justifies the prescription with any cause checked or explicitly described. | | | | |
| TECHNICAL DATE OF INDICATORS: PROCESS | | |  |  |
| **GROUP:** | | Good practices |  |  |
| **AREA:** | | General |  |  |
| Sub-area | | Use of antibiotics |  |  |
| **Nº** | | 3 (of 4) |  |  |

| **INDICATOR NAME** | Prescription of antibiotics in the newborn |
| --- | --- |
| **FORM OF MEASUREMENT** | Sampling of records of women and their newborn attended for childbirth |
| **DESCRIPTION** | Percentage of newborns who are prescribed antibiotics |
| **Level of evidence; Force recommendation** | D - NICE. Coto, 2006. Fernández, 2008. |
| **INDICATOR FORMULA** | Number who are prescribed antibiotics at any time until discharge |
| **Numerator** |  |
| **Denominator** | Number of newborns alive at birth |
| **DATA SOURCE** | Mother and newborn clinical records  SCC (if applicable) |
| **INDICATOR ELABORATION** | Individual |
| **BIBLIOGRAPHIC REFERENCES**  World Health Organization (WHO). WHO - Safe childbirth checklist [Internet]. Available from: <http://www.who.int/patientsafety/implementation/checklists/childbirth/en/>  ^1^ Prevención, diagnóstico y tratamiento de sepsis y choque séptico del recién nacido en el segundo nivel y tercer nivel de atención, México: Secretaría de Salud; Noviembre 2012. | |
| **OBSERVATIONS**  Antibiotic prescription:  Any indication or prescription of antibiotic in the clinical file will be valid throughout the care process (from birth to discharge). The phase of the care process will be captured (immediate postpartum or before discharge) in which the prescription is made, although the indicator is reported without distinction of the phase.  For purposes of validation of the SCC, the marking can be checked regardless of the cause justifying it, either for the overall process or for the antibiotic start (prescription) phase. | |

| TECHNICAL DATE OF INDICATORS: PROCESS | |
| --- | --- |
| **GROUP:** | Good practices |
| **AREA:** | General |
| Sub-area | Use of antibiotics |
| **Nº** | 4 (of 4) |
| **INDICATOR NAME** | Management of antibiotics in the newborn |
| **FORM OF MEASUREMENT** | Sampling of records of women and their newborn attended for childbirth |
| **DESCRIPTION** | Percentage of newborns who are prescribed antibiotic and it is justified by any symptom |
| **Level of evidence; Force recommendation** | D - NICE. Coto, 2006. Fernández, 2008. |
| **INDICATOR FORMULA** | Number e of newborns who are prescribed antibiotic and it is justified by any symptom |
| **Numerator** |  |
| **Denominator** | Number who are prescribed antibiotics at any time until discharge |
| **DATA SOURCE** | Mother and newborn clinical records  SCC (if applicable) |
| **INDICATOR ELABORATION** | Individual |
| **BIBLIOGRAPHIC REFERENCES**   - World Health Organization (WHO). WHO - Safe childbirth checklist [Internet]. Available from:   <http://www.who.int/patientsafety/implementation/checklists/childbirth/en/>  ^1^ Prevención, diagnóstico y tratamiento de sepsis y choque séptico del recién nacido en el segundo nivel y tercer nivel de atención, México: Secretaría de Salud; noviembre 2012. | |
| **OBSERVATIONS**  need for antibiotics in the newborn if:  - Rapid breathing (> 60 breaths / min) or slow (<30 / min)  - Chest numbness, intercostal stretch, noise, convulsions  - Chorioamnionitis  - Little or no mobility to stimulation  - The mother was given antibiotics  - Premature rupture of membranes (18h) 1  - Very cold temperature (<35 ° C and not heated) or high temperature (> 38 ° C)  In the case of SCC, it can be considered that there is a symptom that justifies the prescription with any cause checked or explicitly described. | |

| TECHNICAL DATE OF INDICATORS: PROCESS | |
| --- | --- |
| **GROUP:** | Good practices |
| **AREA:** | General |
| Sub-area | Use of magnesium sulphate |
| **Nº** | 1 (of 2) |
| **INDICATOR NAME** | Prescription of magnesium sulfate during childbirth |
| **FORM OF MEASUREMENT** | Sampling of files of women who were attended for reasons of childbirth |
| **DESCRIPTION** | Percentage of women who are prescribed magnesium sulfate during the labor process |
| **Level of evidence; Force recommendation** | Not applicable |
| **INDICATOR FORMULA** | Number of women during the labor process who are prescribed magnesium sulfate |
| **Numerator** |  |
| **Denominator** | Number of women attended due to bildbirth |
| **DATA SOURCE** | Clinical records  SCC (if applicable) |
| **INDICATOR ELABORATION** | Individual |
| **BIBLIOGRAPHIC REFERENCES**   - World Health Organization (WHO). WHO - Safe childbirth checklist [Internet]. Available from: <http://www.who.int/patientsafety/implementation/checklists/childbirth/en/> | |
| **OBSERVATIONS**  Prescription of magnesium sulphate:  Any indication or prescription of magnesium sulphate in the clinical file during the entire delivery process (from admission to immediate postpartum) will be valid. It will capture the phase of the care process (admission, pre-expulsion or immediate postpartum) in which the indication or prescription is made, although the indicator is reported for the overall process of delivery care.  For purposes of validation of the SCC, it is possible to contrast the marking in this one independently of the justifying cause, either for the overall process or for the magnesium sulfate start-up phase (prescription). | |

| TECHNICAL DATE OF INDICATORS: PROCESS | |
| --- | --- |
| **GROUP:** | Good practices |
| **AREA:** | General |
| Sub-area | Use of magnesium sulphate |
| **Nº** | 2 (of 2) |
| **INDICATOR NAME** | Management of magnesium sulfate at delivery for preeclampsia / eclampsia control |
| **FORM OF MEASUREMENT** | Sampling of files of women who were attended for reasons of labor and were prescribed magnesium sulfate |
| **DESCRIPTION** | Percentage of women who are prescribed magnesium sulfate during the childbirth process and it is justified |
| **Level of evidence; Force recommendation** | Evidencia: Ia-[E. Shekelle] - McDonald SD, 2012.- Duley L, 2010. (Enf. Eclampsia)  IV-[E. Shekelle] - Diemunsh P, et.al. 2010- (ER obst)  Recomendación: A-[E. Shekelle] - Duley L, 2010.(Enf. Eclampsia) |
| **INDICATOR FORMULA** | Number of women who are prescribed magnesium sulfate during the childbirth process and it is justified |
| **Numerator** |  |
| **Denominator** | Number of women during the labor process who are prescribed magnesium sulfate |
| **DATA SOURCE** | Clinical records  SCC (if applicable) |
| **INDICATOR ELABORATION** | Individual |
| **BIBLIOGRAPHIC REFERENCES**   - World Health Organization (WHO). WHO - Safe childbirth checklist [Internet]. Available from:   <http://www.who.int/patientsafety/implementation/checklists/childbirth/en/>  1. Detección y Tratamiento Inicial de las Emergencias Obstétricas. México: Secretaria de Salud, elaboración 2010 y actualización 2011. | |
| **OBSERVATIONS**  In all stages: need for magnesium sulfate if the patient has:  - Diastolic pressure ≥110 mmHg and proteinuria 3+ (see table 1)  - Diastolic pressure ≥90 mmHg, proteinuria 2+ (see Table 1) and any of the following: severe headache and / or blurred vision  - Epigastric pain  It is contraindicated for the prescription of magnesium sulfate (it will be considered non-compliance): renal insufficiency, myocardial injury, heart block and myasthenia gravis.  In the case of SCC, it can be considered that there is a symptom that justifies the prescription with any cause checked or explicitly described. | |

| TECHNICAL DATE OF INDICATORS: PROCESS | |
| --- | --- |
| **GROUP:** | Good practices |
| **AREA:** | General |
| Sub-area | Bleeding control |
| **Nº** | 1 (of 1) |
| **INDICATOR NAME** | adequate management of hemorrhage after childbirth |
| **FORM OF MEASUREMENT** | Percentage of women with adequate management of postpartum hemorrhage |
| **DESCRIPTION** | Percentage of postpartum patients with adequate bleeding management |
| **Level of evidence; Force recommendation** | Expert opinion |
| **INDICATOR FORMULA** | Number of postpartum women who are given the necessary actions for postprtum bleeding or hemorrhage |
| **Numerator** |  |
| **Denominator** | Number of women who needed actions to manage postpartum hemorrhage |
| **DATA SOURCE** | Clinical records  SCC (if applicable) |
| **INDICATOR ELABORATION** | Individual |
| **BIBLIOGRAPHIC REFERENCES**   - World Health Organization (WHO). WHO - Safe childbirth checklist [Internet]. Available from:   <http://www.who.int/patientsafety/implementation/checklists/childbirth/en/>   - Organización Mundial de la Salud. Concentraciones de hemoglobina para diagnosticar la anemia y evaluar su gravedad. Ginebra, Organización Mundial de la Salud, 2011 (WHO/NMH/NHD/MNM/11.1) <http://www.who.int/vmnis/indicators/haemoglobin_es.pdf> (consultado el 08/07/2015). - Prevención y manejo de la hemorragia obstétrica en el primer, segundo y tercer niveles de atención – SS-103-08. Secretaría de salud. México. Actualización 2013. - Biblioteca de Salud Reproductiva de la OMS. Tratamiento para la hemorragia postparto primaria. Available from: <http://apps.who.int/rhl/pregnancy_childbirth/childbirth/postpartum_haemorrhage/sfguide/es/>   (consultado el 23/07/2015). | |
| **OBSERVATIONS**  **COMPONENT INDICATOR**  **Immediate postpartum stage:**  Each of the actions to be performed at this stage is valued independently. Report postpartum actions if there is bleeding ≥500 ml, or if ≥250 ml and severe anemia (hemoglobin less than 7 g / dL)  The review will be considered in the SCC and in the record of each action and of the set (compound):  - Uterine / SCC massage and revised records  - Consider additional uterotonic / SCC and reviewed records  - Initiate via intravenous / SCC and reviewed dossiers  - Activate rapid response team for obstetric / SCC emergencies and reviewed records  - Addressing the case / SCC and reviewed files  - The 5 activities carried out / SCC and reviewed files (compound)  **Step before departure:**  - Excessive bleeding: bleeding ≥ 500 ml, or ≥250 ml and severe anemia (hemoglobin less than 7 g / dL).  - If the patient has uncontrolled hemorrhage, the egress should be treated and delayed (file with "controlled hemorrhage" registered before discharge or checked in SCC). | |

##

## 3.2. Indicators for phase of admission

| TECHNICAL DATE OF INDICATORS: PROCESS | |
| --- | --- |
| **GROUP:** | Good practices |
| **AREA:** | Admission |
| Sub-area | Using partogram |
| **Nº** | 1 (of 2) |
| **INDICATOR NAME** | Partogram opening |
| **FORM OF MEASUREMENT** | Sampling of files of women who were attended for reasons of childbirth |
| **DESCRIPTION** | Percentage of women who underwent partogram opening |
| **Level of evidence; Force recommendation** | A – Shekelle – Sony OMS, 2009  D - Shekelle- NOM-007-SSA2-1993 |
| **INDICATOR FORMULA** | Number of women with partogram started |
| **Numerator** |  |
| **Denominator** | Number of women attended due to labor |
| **DATA SOURCE** | Clinical records: partogram |
| **INDICATOR ELABORATION** | Individual |
| **BIBLIOGRAPHIC REFERENCES**   - World Health Organization (WHO). WHO - Safe childbirth checklist [Internet]. Available from:   <http://www.who.int/patientsafety/implementation/checklists/childbirth/en/>   - Vigilancia y manejo del trabajo de parto en embarazo de bajo riesgo. México: Secretaría de Salud; 11 de diciembre de 2014. ISBN: 978-607-7790-94-5 - Norma Oficial Mexicana NOM-007-SSA2-1993, Atención de la mujer durante el embarazo, parto y puerperio y del recién nacido. Criterios y procedimientos para la prestación del servicio. | |
| **OBSERVATIONS**  Opening: there is the partograph in the file with the patient's data (at least, full name, age or date of birth, weeks of gestation and no clinical file). | |

| TECHNICAL DATE OF INDICATORS: PROCESS | |
| --- | --- |
| **GROUP:** | Good practices |
| **AREA:** | Admission |
| Sub-area | Using partogram |
| **Nº** | 2 (of 2) |
| **INDICATOR NAME** | Filling the partogram |
| **FORM OF MEASUREMENT** | Sampling of files of women who were attended for reasons of childbirth |
| **DESCRIPTION** | Percentage of women who underwent parturition at birth |
| **Level of evidence; Force recommendation** | A – Shekelle – Sony OMS, 2009  D - Shekelle- NOM-007-SSA2-1993 |
| **INDICATOR FORMULA** | Number of women with partogram correctly filled in the file |
| **Numerator** |  |
| **Denominator** | Number of women who were attended due to labor and had a partograph in the file |
| **DATA SOURCE** | Clinical records: partogram  SCC (if applicable) |
| **INDICATOR ELABORATION** | Individual |
| **BIBLIOGRAPHIC REFERENCES**   - World Health Organization (WHO). WHO - Safe childbirth checklist [Internet]. Available from:   <http://www.who.int/patientsafety/implementation/checklists/childbirth/en/>   - Vigilancia y manejo del trabajo de parto en embarazo de bajo riesgo. México: Secretaría de Salud; 11 de diciembre de 2014. ISBN: 978-607-7790-94-5 - Norma Oficial Mexicana NOM-007-SSA2-1993, Atención de la mujer durante el embarazo, parto y puerperio y del recién nacido. Criterios y procedimientos para la prestación del servicio. | |
| **OBSERVATIONS**  **Fill:**  - Every 2 hours: temperature.  - Every 30 min: heart rate of the woman and the fetus, and contractions.  - Every 4 hours: blood pressure.  - Touch / dilation according to evolution and medical indication: there must be an explicit check in SCC and / or registration in the file of touch / dilation (the registration of cm of dilation will be taken as valid) | |

| TECHNICAL DATE OF INDICATORS: PROCESS | |
| --- | --- |
| **GROUP:** | Good practices |
| **AREA:** | Admission |
| Sub-area | Use of antiretrovirals |
| **Nº** | 1 (of 1) |
| **INDICATOR NAME** | Management of anti-retroviral at birth |
| **FORM OF MEASUREMENT** | Review of dossiers of HIV-infected women attended |
| **DESCRIPTION** | Percentage of women with HIV in labor in whom antiretroviral treatment is started |
| **Level of evidence; Force recommendation** | A: [E. Shekelle]. Volmink, J. 2009 |
| **INDICATOR FORMULA** | Number of women (HIV +) who started antiretroviral during labor |
| **Numerator** |  |
| **Denominator** | Number of women with HIV + attended due to childbirth |
| **DATA SOURCE** | Clinical records  SCC (if applicable) |
| **INDICATOR ELABORATION** | Individual |
| **BIBLIOGRAPHIC REFERENCES**   - World Health Organization (WHO). WHO - Safe childbirth checklist [Internet]. Available from:   <http://www.who.int/patientsafety/implementation/checklists/childbirth/en/>   - Prevención, diagnóstico y tratamiento del binomio madre-hijo con infección por el VIH. México. Instituto Mexicano del Seguro Social, 2009; actualización junio 2012 | |
| **OBSERVATIONS**  For prevention of HIV transmission from the mother to the newborn during delivery, antiretroviral therapy should be administered from the start of labor (or at any time from the time of admission of labor to delivery) to the birth of newborn.  In the SCC, the initiation of anti-retroviral (ARV) must be recorded in the admission phase  The mother's medical record must be recorded ARV prescription always before birth. | |

| TECHNICAL DATE OF INDICATORS: PROCESS | |
| --- | --- |
| **GROUP:** | Good practices |
| **AREA:** | Admission |
| Sub-area | Patient-centered care |
| **Nº** | 1 (of 1) |
| **INDICATOR NAME** | Promotion of accompanying presence during childbirth |
| **FORM OF MEASUREMENT** | Postpartum women survey |
| **DESCRIPTION** | Percentage of women in labor whose companion is informed and encouraged to be present at delivery |
| **Level of evidence; Force recommendation** | Ia-[E. Shekelle] – Hodnett. Cochrane 2013.  IV-[E. Shekelle] – Martis BSR OMS 2007.  A-GPC Ministerio de Salud, 2010. |
| **INDICATOR FORMULA** | N ° of women in labor whose companion is informed and encouraged to be present at delivery since admission |
| **Numerator** |  |
| **Denominator** | Number of women attended due to labor |
| **DATA SOURCE** | Questionnaire to the mother  SCC (if applicable) |
| **INDICATOR ELABORATION** | Individual |
| **BIBLIOGRAPHIC REFERENCES**   - World Health Organization (WHO). WHO - Safe childbirth checklist [Internet]. Available from:   <http://www.who.int/patientsafety/implementation/checklists/childbirth/en/>   - Vigilancia y manejo del trabajo de parto en embarazo de bajo riesgo. México: Secretaría de Salud; 11 de diciembre de 2014. ISBN: 978-607-7790-94-5   ^1^ Biblioteca de Salud reproductiva de la OMS. Apoyo continuo a las mujeres durante el parto. http://apps.who.int/rhl/pregnancy_childbirth/childbirth/routine_care/rmcom/es/ | |
| **OBSERVATIONS**  If the mother is in a position to decide, she will be the one to determine who is her companion; provided that the hospital has the necessary structure to allow the presence of the companion.  Exceptionally, all cases in which there was no companion or the structure of the center does not allow it.  However, WHO notes1:  "The argument that it invades other women's privacy often can not be sustained, as other staff members such as housekeepers, catering and students also enter the prep room without introducing themselves and could invade the privacy of all women. Most hospitals offer curtains that can be used if privacy is a real problem. " | |

## 3.3. Indicators for pre-expulsive phase (or before cesarean section)

| TECHNICAL DATE OF INDICATORS: PROCESS | |
| --- | --- |
| **GROUP:** | Good practices |
| **AREA:** | Pre-expulsive phase |
| Sub-area | Interventions in childbirth |
| **Nº** | 1 (of 3) |
| **INDICATOR NAME** | Resolution of cesarean delivery justified |
| **FORM OF MEASUREMENT** | Sampling of records of women who were attended for reasons of labor and who underwent cesarean delivery |
| **DESCRIPTION** | Percentage of women with justified cesarean delivery |
| **Level of evidence; Force recommendation** | Expert opinion |
| **INDICATOR FORMULA** | Number of women undergoing cesarean delivery under a justified indication |
| **Numerator** |  |
| **Denominator** | Number of women undergoing cesarean delivery |
| **DATA SOURCE** | Clinical records  SCC (if applicable) |
| **INDICATOR ELABORATION** | Individual |
| **BIBLIOGRAPHIC REFERENCES**   - Guía de Práctica Clínica para la Reducción de la Frecuencia de Operación Cesárea México: Instituto Mexicano de Seguro social; 2014. ISBN: 978-607-7790-92-1 | |
| **OBSERVATIONS**  Correct and justified indication for cesarean section:  - 2 previous cesareans  - transverse situation  - twin pregnancy  - pelvic presentation  - Class III and IV heart disease  - fetal hydrocephalus  placenta previa  - abdominal cerclage  - macrosomía  - unstable fetal status  - fetal malformations  - active genital herpes  - tumor that obstructs the birth canal  - history of uterine surgery  - premature placental abruption  HIV  - death product> 30 weeks gestation (SDG) in patient without labor for more than 24 hrs. | |

| TECHNICAL DATE OF INDICATORS: PROCESS | |
| --- | --- |
| **GROUP:** | Good practices |
| **AREA:** | Pre-expulsive phase |
| Sub-area | Interventions in childbirth |
| **Nº** | 2 (of 3) |
| **INDICATOR NAME** | Performed instrumented childbirth justified |
| **FORM OF MEASUREMENT** | Sampling of records of women who were attended for reasons of labor and who were given instrumental delivery |
| **DESCRIPTION** | Percentage of women with instrumented childbirth justified |
| **Level of evidence; Force recommendation** | IIb - Guía de Práctica Clínica- Parto Instrumental. 2010  C - ACOG, 2004 |
| **INDICATOR FORMULA** | Number of women with justified indication of instrumented delivery |
| **Numerator** |  |
| **Denominator** | Number of women with instrumented delivery |
| **DATA SOURCE** | Clinical records  SCC (if applicable) |
| **INDICATOR ELABORATION** | Individual |
| **BIBLIOGRAPHIC REFERENCES**  Guía de Práctica Clínica para la Reducción de la Frecuencia de Operación Cesárea México: Instituto Mexicano de Seguro social; 2014. ISBN: 978-607-7790-92-1 | |
| **OBSERVATIONS**  Correct and justified indication for instrumented delivery (forceps):  **Fetal Causes:**  - Presumptive fetal / expulsive commitment with fetal instability  **Maternal causes:**  - Medical diseases to prevent valsalva (eg Maternal disease Functional class III-IV)  - Prolonged Expulsive  - Maternal fatigue / exhaustion  - Previous Cesarean section  - Maternal heart disease | |

| TECHNICAL DATE OF INDICATORS: PROCESS | |
| --- | --- |
| **GROUP:** | Good practices |
| **AREA:** | Pre-expulsive phase |
| Sub-area | Interventions in childbirth |
| **Nº** | 3 (of 3) |
| **INDICATOR NAME** | Performed justified episiotomy |
| **FORM OF MEASUREMENT** | Sampling of records of women who were attended for reasons of labor and who underwent episiotomy |
| **DESCRIPTION** | Percentage of women with justified episiotomy at delivery |
| **Level of evidence; Force recommendation** | A - GPC Ministerio de Sanidad y Política Social, 2010  A - GPC Prevención, diagnóstico y tratamiento de la episiotomías complicada IMSS, 2013 |
| **INDICATOR FORMULA** | Number of women with justified indication of episiotomy |
| **Numerator** |  |
| **Denominator** | Number of women with episiotomy at delivery |
| **DATA SOURCE** | Clinical records  SCC (if applicable) |
| **INDICATOR ELABORATION** | Individual |
| **BIBLIOGRAPHIC REFERENCES**   - Vigilancia y manejo del trabajo de parto en embarazo de bajo riesgo. México: Secretaría de Salud; 11 de diciembre de 2014. ISBN: 978-607-7790-94-5 - Norma Oficial Mexicana NOM-007-SSA2-1993, Atención de la mujer durante el embarazo, parto y puerperio y del recién nacido. Criterios y procedimientos para la prestación del servicio. | |
| **OBSERVATIONS**  Routine episiotomy should not be practiced at all spontaneous deliveries.  The episiotomy should be performed if there is clinical need, so the following will be considered:  - Instrumental birth  - Short and / or rigid perineum  - Shoulder dystocia in the fetus | |

| TECHNICAL DATE OF INDICATORS: STRUCTURE | |
| --- | --- |
| **GROUP:** | Good practices |
| **AREA:** | Pre-expulsive phase |
| Sub-area | Infrastructure |
| **Nº** | 1 (de 2) |
| **INDICATOR NAME** | Availability of maternal care supplies immediately before delivery |
| **FORM OF MEASUREMENT** | Sampling of records of women who were attended for childbirth: SCC review; or conducting a survey |
| **DESCRIPTION** | Availability of maternal care supplies immediately before delivery |
| **Level of evidence; Force recommendation** | Not applicable |
| **INDICATOR FORMULA** | Availability of maternal care inputs (each input and the whole) |
| **Numerator** |  |
| **Denominator** | Number of SCC reviewed |
| **DATA SOURCE** | SCC  Ad hoc questionnaire for professionals |
| **INDICATOR ELABORATION** | Individual |
| **BIBLIOGRAPHIC REFERENCES**  World Health Organization (WHO). WHO - Safe childbirth checklist [Internet]. Available from:  <http://www.who.int/patientsafety/implementation/checklists/childbirth/en/> | |
| **OBSERVATIONS**  Report of availability of each input and of the set (100%):  -Clean water  - Soap  - Disposable towels (toilets)  - Gloves  - Uterotonic: oxytocin 10 IU in syringe No. of SCC reviewed  - 2 ring tongs  Information validation tool: survey of professionals  The "always" response category will be taken into account as an available input.  The remaining categories will be taken into account as no availability of inputs. | |

| TECHNICAL DATE OF INDICATORS: STRUCTURE | |
| --- | --- |
| **GROUP:** | Good practices |
| **AREA:** | Pre-expulsive phase |
| Sub-area | Infrastructure |
| **Nº** | 2 (of 2) |
| **INDICATOR NAME** | Availability of supplies for newborn care immediately before delivery |
| **FORM OF MEASUREMENT** | Sampling of records of women who were attended for childbirth: SCC review; or conducting a survey |
| **DESCRIPTION** | Availability of supplies for newborn care immediately before delivery |
| **Level of evidence; Force recommendation** | Not applicable |
| **INDICATOR FORMULA** | Availability of supplies for care of the newborn (each input and the whole) |
| **Numerator** |  |
| **Denominator** | Number of SCC reviewed |
| **DATA SOURCE** | SCC  Ad hoc questionnaire for professionals |
| **INDICATOR ELABORATION** | Individual |
| **BIBLIOGRAPHIC REFERENCES**   - World Health Organization (WHO). WHO - Safe childbirth checklist [Internet]. Available from: <http://www.who.int/patientsafety/implementation/checklists/childbirth/en/> - Atención del recién nacido sano; México: Secretaría de Salud; 2009 | |
| **OBSERVATIONS**  SCC Availability Report: of each input and of the set (100%):  - Clean towels or fields  - Scissors for cord cutting  - Suction knob  - Vitamin K  - Ophthalmic prophylaxis  - Piping equipment  - Laryngoscope  - Umbilical cord tape Number of SCC reviewed  - O2 source and aspiration  - Adrenaline  - Intubation Cannulas  - Saline and Glucose Solution  - Mask and bag for ventilation (ambú)  Information validation tool: survey of professionals  The "always" response category will be taken into account as an available input.  The remaining categories will be taken into account as no availability of inputs. | |

## 3.4. Indicators for immediate postpartum phase (first hour after expulsion)

| TECHNICAL DATE OF INDICATORS: PROCESS | |
| --- | --- |
| **GROUP:** | Good practices |
| **AREA:** | Immediate postpartum |
| Sub-area | Mother's care |
| **Nº** | 1 (of 2) |
| **INDICATOR NAME** | Care for the mother immediately after birth |
| **FORM OF MEASUREMENT** | Sampling of records of women who were cared for during delivery: review of SCC |
| **DESCRIPTION** | Percentage of women who were given necessary actions immediately after birth. |
| **Level of evidence; Force recommendation** | A, B - (GPC Ministerio de Sanidad y Política Social, 2010) - Fuerte (WHO recommendations for the prevention and treatment of postpartum haemorrhage, 2012).  D - Shekelle - FIGO Guidelines. Prevention and treatment of postpartum hemorrhage in low-resource settings, 2012. |
| **INDICATOR FORMULA** | Women who were given the necessary actions immediately after birth (see comments) |
| **Numerator** |  |
| **Denominator** | Total records reviewed |
| **DATA SOURCE** | Medical records  SCC |
| **INDICATOR ELABORATION** | Individual |
| **BIBLIOGRAPHIC REFERENCES**  ^1^ World Health Organization (WHO). WHO - Safe childbirth checklist [Internet]. Available from: <http://www.who.int/patientsafety/implementation/checklists/childbirth/en/>  ^2^ Vigilancia y manejo del trabajo de parto en embarazo de bajo riesgo. México: Secretaría de Salud; 11 de diciembre de 2014. ISBN: 978-607-7790-94-5 | |
| **OBSERVATIONS**  Report of necessary actions to care for the mother immediately after birth  - **Oxytocin administration in the first minute1,2** / SCC and reviewed files  - **Control traction of the umbilical cord** 1,2 for the removal of the placenta / SCC and revised files  - **Uterus massage** 1,2 after removal of the placenta / SCC and revised files  Value% of compliance of each of the actions.  Value% of compliance of composite indicator. It is necessary to comply with 100% of the four components. | |

| TECHNICAL DATE OF INDICATORS: PROCESS | |
| --- | --- |
| **GROUP:** | Good practices |
| **AREA:** | Immediate postpartum |
| Sub-area | Mother's care |
| **Nº** | 2 (of 2) |
| **INDICATOR NAME** | Disconfirmed presence of second product |
| **FORM OF MEASUREMENT** | Sampling of records of women who were cared for during delivery: review of SCC |
| **DESCRIPTION** | Percentage of women in whom the presence of a second product is reviewed and disconfirmed |
| **Level of evidence; Force recommendation** |  |
| **INDICATOR FORMULA** | Women in whom a second product is reviewed and disconfirmed |
| **Numerator** |  |
| **Denominator** | Total records reviewed |
| **DATA SOURCE** | Medical records  SCC |
| **INDICATOR ELABORATION** | Individual |
| **BIBLIOGRAPHIC REFERENCES**  ^1^ World Health Organization (WHO). WHO - Safe childbirth checklist [Internet]. Available from: <http://www.who.int/patientsafety/implementation/checklists/childbirth/en/> | |
| **OBSERVATIONS**  Report of necessary actions to care for the mother immediately after birth  - Disconfirmed presence of a second baby 1 / SCC and reviewed files | |

| TECHNICAL DATE OF INDICATORS: PROCESS | |
| --- | --- |
| **GROUP:** | Good practices |
| **AREA:** | Immediate postpartum |
| Sub-area | Care of the newborn |
| **Nº** | 1 (of 5) |
| **INDICATOR NAME** | Immediate baby care |
| **FORM OF MEASUREMENT** | Sampling of records of women cared for during labor and their newborns |
| **DESCRIPTION** | Percentage of newborns who were given necessary care actions |
| **Level of evidence; Force recommendation** | **Profilaxis oftálmica**  A, C – NICE, 2006.  **Vitamina K**  A, D – NICE, 2006.  **Secado**  A – NICE, 2006. |
| **INDICATOR FORMULA** | Total newborns who were correctly followed up with the necessary actions in the immediate postpartum period |
| **Numerator** |  |
| **Denominator** | Number of newborns |
| **DATA SOURCE** | Clinical record of the mother; Sheet of the newborn; SCC (if applicable) |
| **INDICATOR ELABORATION** | Individual |
| **BIBLIOGRAPHIC REFERENCES**  World Health Organization (WHO). WHO - Safe childbirth checklist [Internet]. Available from: <http://www.who.int/patientsafety/implementation/checklists/childbirth/en/>  ^1^ Vigilancia y manejo del trabajo de parto en embarazo de bajo riesgo. México: Secretaría de Salud; 2014. ISBN: 978-607-7790-94-5  ^2^ Atención del recién nacido sano; México: Secretaría de Salud; 2009.  ^3^ Norma Oficial Mexicana NOM-007-SSA2-1993, Atención de la mujer durante el embarazo, parto y puerperio y del recién nacido. Criterios y procedimientos para la prestación del servicio. | |
| **OBSERVATIONS**  Value% of compliance of each of the actions. Value% of compliance with the composite indicator (compliance of 100% of the four components).  Proper care of the newborn:  - Dry the baby 1,2 and keep it warm2  - Administer vitamin K2,3  - Administering ophthalmic prophylaxis2 | |

| TECHNICAL DATE OF INDICATORS: PROCESS | |
| --- | --- |
| **GROUP:** | Good practices |
| **AREA:** | Immediate postpartum |
| Sub-area | Care of the newborn |
| **Nº** | 2 (of 5) |
| **INDICATOR NAME** | Delayed clamping of the umbilical cord in the newborn |
| **FORM OF MEASUREMENT** | Sampling of records of women cared for during labor and their newborns |
| **DESCRIPTION** | Percentage of newborns who underwent late-stage impingement of the newborn |
| **Level of evidence; Force recommendation** | **Delay in cutting the cord**  1+; GPC Ministerio de Sanidad y Política Social, 2010  IV; Shekelle - FIGO Guidelines. Prevention and treatment of postpartum hemorrhage in low-resource settings, 2012 |
| **INDICATOR FORMULA** | Total, newborns who underwent late-term impingement of the newborn as part of immediate postpartum actions |
| **Numerator** |  |
| **Denominator** | Number of newborns |
| **DATA SOURCE** | Clinical record of the mother; Sheet of the newborn; SCC (if applicable) |
| **INDICATOR ELABORATION** | Individual |
| **BIBLIOGRAPHIC REFERENCES**   - World Health Organization (WHO). WHO - Safe childbirth checklist [Internet]. Available from: <http://www.who.int/patientsafety/implementation/checklists/childbirth/en/>   ^1^ Vigilancia y manejo del trabajo de parto en embarazo de bajo riesgo. México: Secretaría de Salud; 2014. ISBN: 978-607-7790-94-5 | |
| **OBSERVATIONS**  - Delay in cutting the cord1 - Delayed clamping of the umbilical cord in the active management of the third period of labor (1 to 3 minutes after birth) or cessation of umbilical cord beating is recommended.  Exclusion Criteria: Instability of the mother and / or newborn. | |

| TECHNICAL DATE OF INDICATORS: PROCESS | |
| --- | --- |
| **GROUP:** | Good practices |
| **AREA:** | Immediate postpartum |
| Sub-area | Care of the newborn |
| **Nº** | 3 (of 5) |
| **INDICATOR NAME** | Skin-to-skin contact |
| **FORM OF MEASUREMENT** | Sampling of records of women cared for during labor and their newborns |
| **DESCRIPTION** | Percentage of newborns in whom skin-to-skin contact begins immediately |
| **Level of evidence; Force recommendation** | **Skin with immediate skin**  A - Shekelle. Moore, 2012  A - Guía de Práctica Clínicas sobre la Atención al Parto Normal, Ministerio de Sanidad y Consumo de España, 2010. |
| **INDICATOR FORMULA** | Total newborns in whom skin-to-skin contact begins immediately after birth |
| **Numerator** |  |
| **Denominator** | Number of newborns |
| **DATA SOURCE** | Clinical record of the mother; Sheet of the newborn; SCC (if applicable) |
| **INDICATOR ELABORATION** | Individual |
| **BIBLIOGRAPHIC REFERENCES**   - World Health Organization (WHO). WHO - Safe childbirth checklist [[ - Internet]. Available from: <http://www.who.int/patientsafety/implementation/checklists/childbirth/en/>   ^1^ Atención del recién nacido sano; México: Secretaría de Salud; 2009  ^2^ Vigilancia y manejo del trabajo de parto en embarazo de bajo riesgo. México: Secretaría de Salud; 2014. ISBN: 978-607-7790-94-5  ^3^ Atención del recién nacido sano; México: Secretaría de Salud; 2009. | |
| **OBSERVATIONS**  - **Skin with immediate skin1, 2, 3** - Early contact skin and skin of healthy mothers and newborns is recommended after birth, as it improves cardiorespiratory stability and increases glucose in newborns. And stay with your mother at all times after childbirth, as long as the state of health of the mother and newborn allow. If the mother's health condition does not allow it, the father will be offered the possibility of making skin-to-skin contact with the newborn.  **Exclusion Criteria**: Instability of the mother and / or newborn.  Validation tool: mothers survey. | |

| TECHNICAL DATE OF INDICATORS: PROCESS | |
| --- | --- |
| **GROUP:** | Good practices |
| **AREA:** | Immediate postpartum |
| Sub-area | Care of the newborn |
| **Nº** | 4 (of 5) |
| **INDICATOR NAME** | Breastfeeding |
| **FORM OF MEASUREMENT** | Sampling of records of women cared for during labor and their newborns |
| **DESCRIPTION** | Percent of newborn infants who initiate immediate breastfeeding |
| **Level of evidence; Force recommendation** | **LM inmediata**  D – Shekelle. PROYECTO de Norma Oficial Mexicana PROY-NOM-007-SSA2-2010, Para la atención de la mujer durante el embarazo, parto y puerperio, y del recién nacido, 2012. |
| **INDICATOR FORMULA** | Total newborns in which breastfeeding begins immediately after delivery (the first hour after expulsion) |
| **Numerator** |  |
| **Denominator** | Number of newborns |
| **DATA SOURCE** | Clinical record of the mother; Sheet of the newborn; SCC (if applicable) |
| **INDICATOR ELABORATION** | Individual |
| **BIBLIOGRAPHIC REFERENCES**  World Health Organization (WHO). WHO - Safe childbirth checklist [Internet]. Available from: <http://www.who.int/patientsafety/implementation/checklists/childbirth/en/>  ^1^ Atención del recién nacido sano; México: Secretaría de Salud; 2009  ^2^ Vigilancia y manejo del trabajo de parto en embarazo de bajo riesgo. México: Secretaría de Salud; 2014. ISBN: 978-607-7790-94-5  ^3^ Atención del recién nacido sano; México: Secretaría de Salud; 2009. | |
| **OBSERVATIONS**  - Immediate LM (> 1h) 1, 2, 3 - Initiate exclusive breastfeeding on demand within the first 30 minutes of the newborn's life, in those women whose conditions allow it.  Exclusion Criteria: Instability of the mother and / or newborn. Do not initiate or avoid LM in the case of mothers with HIV + or in treatment with a contraindicated drug for LM  Validation tool: mothers survey. | |

| TECHNICAL DATE OF INDICATORS: PROCESS | |
| --- | --- |
| **GROUP:** | Good practices |
| **AREA:** | Immediate postpartum |
| Sub-area | Care of the newborn |
| **Nº** | 5 (of 5) |
| **INDICATOR NAME** | Management of antiretroviral drugs in the newborn |
| **FORM OF MEASUREMENT** | Sampling or total of records of women with HIV who were cared for during delivery and their newborn |
| **DESCRIPTION** | Percentage of newborns receiving antiretroviral therapy started |
| **Level of evidence; Force recommendation** | C – Shekelle. Public Health Service Task Force Recommendations for Use of Antiretroviral Drugs in Pregnant HIV-Infected Women for materna Health and Interventions to Reduce Perinatal HIV Transmission in the United States, 2009. |
| **INDICATOR FORMULA** | Number of infants receiving antiretroviral therapy |
| **Numerator** |  |
| **Denominator** | Total number of newborns of mothers with HIV + |
| **DATA SOURCE** | Clinical Records / Newborn Story  SCC (if applicable) |
| **INDICATOR ELABORATION** | Individual |
| **BIBLIOGRAPHIC REFERENCES**  World Health Organization (WHO). WHO - Safe childbirth checklist [Internet]. Available from: <http://www.who.int/patientsafety/implementation/checklists/childbirth/en/>   - WHO recommendations for Prevention and treatment of pre-eclampsia and eclampsia. Geneva: World Health Organization, 2011. - Prevención, diagnóstico y tratamiento en el binomio madre-hijo con infección por el VIH. México, Instituto Mexicano del Seguro Social, 2009; actualización en junio, 2012. | |
| **OBSERVACIONES**  El recién nacido debe recibir tratamiento en las primeras 6h si la madre presenta VIH+. | |

## 3.5. Indicators for phase just before discharge

| TECHNICAL DATE OF INDICATORS: PROCESS | |
| --- | --- |
| **GROUP:** | Good practices |
| **AREA:** | Before Exit |
| Sub-area | Mother's care |
| **Nº** | 1 (of 2) |
| **INDICATOR NAME** | Information on family planning prior to discharge |
| **FORM OF MEASUREMENT** | Sampling of files of women attended for reasons of childbirth; Conducting a survey |
| **DESCRIPTION** | Percentage of women with information on family planning prior to discharge |
| **Level of evidence; Force recommendation** | Not found |
| **INDICATOR FORMULA** | Number of women reported, explained, or presented with family planning prior to discharge |
| **Numerator** |  |
| **Denominator** | Total number of women graduated after childbirth |
| **DATA SOURCE** | SCC (if applicable)  Questionnaire to mothers |
| **INDICATOR ELABORATION** | Individual |
| **BIBLIOGRAPHIC REFERENCES**   - World Health Organization (WHO). WHO - Safe childbirth checklist [Internet]. Available from:   <http://www.who.int/patientsafety/implementation/checklists/childbirth/en/>   - Norma Oficial Mexicana NOM-007-SSA2-1993, Atención de la mujer durante el embarazo, parto y puerperio y del recién nacido. Criterios y procedimientos para la prestación del servicio. | |
| **OBSERVATIONS**  The mother will be guided in the care of the newborn, exclusive breastfeeding, family planning methods, maternal feeding and about the emotional changes that may occur during the postpartum period. | |

| TECHNICAL DATE OF INDICATORS: PROCESS | |
| --- | --- |
| **GROUP:** | Good practices |
| **AREA:** | Before Exit |
| Sub-area | Mother's care |
| **Nº** | 2 (of 2) |
| **INDICATOR NAME** | Information about the mother's warning signs |
| **FORM OF MEASUREMENT** | Sampling of files of women attended for reasons of childbirth;  Conducting a survey  SCC |
| **DESCRIPTION** | Percentage of women and / or companions were informed about the warning signs of the mother to ask for help |
| **Level of evidence; Force recommendation** | Not applicable |
| **INDICATOR FORMULA** | Number of women and / or companions who were informed of the warning signs of the mother before whom she should ask for help in the discharge |
| **Numerator** |  |
| **Denominator** | Number of women attended due to labor |
| **DATA SOURCE** | Questionnaire for woman / companion  Review of the SCC |
| **INDICATOR ELABORATION** | Individual |
| **BIBLIOGRAPHIC REFERENCES**  World Health Organization (WHO). WHO - Safe childbirth checklist [Internet]. Available from:  <http://www.who.int/patientsafety/implementation/checklists/childbirth/en/> | |
| **OBSERVATIONS**  The alarm signs to which assistance should be requested to the discharge described in the SCC (for each sign and of the set -compound indicator type 100% compliance) are valued:  - Bleeding  - Severe abdominal pain  -Severe headache  - Alteration of the state of consciousness  - Visual alterations n ° of SCC reviewed  - Difficulty breathing  - Difficulty emptying the bladder  - Fever and / or chills  Information contrast tool: mothers survey. However, only the symptoms and signs that are agreed upon will be recorded. | |

| TECHNICAL DATE OF INDICATORS: PROCESS | |
| --- | --- |
| **GROUP:** | Good practices |
| **AREA:** | Before Exit |
| Sub-area | Care of mother and newborn |
| **Nº** | 1 (of 1) |
| **INDICATOR NAME** | Organization and agreements on the monitoring of mother and newborn |
| **FORM OF MEASUREMENT** | Sampling of files of women attended for reasons of childbirth;  Conducting a survey  SCC |
| **DESCRIPTION** | Percentage of women who know the visits they must make (them and the newborn) and the place to go to the reviews before they are discharged |
| **Level of evidence; Force recommendation** | Unidentified |
| **INDICATOR FORMULA** | Number of women who are informed prior to their departure from the visits they should make (them and the newborn) |
| **Numerator** |  |
| **Denominator** | Total number of women postpartum |
| **DATA SOURCE** | SCC (if applicable)  Questionnaire to mothers at the time of discharge |
| **INDICATOR ELABORATION** | Individual |
| **BIBLIOGRAPHIC REFERENCES**   - World Health Organization (WHO). WHO - Safe childbirth checklist [Internet]. Available from:   <http://www.who.int/patientsafety/implementation/checklists/childbirth/en/>   - Norma Oficial Mexicana NOM-007-SSA2-1993, Atención de la mujer durante el embarazo, parto y puerperio y del recién nacido. Criterios y procedimientos para la prestación del servicio. | |
| **OBSERVATIONS**  - **Mother:**  Immediate puerperium (2nd to 7th day) and late (8th to 42nd day): A minimum of three visits should be provided, covering the period of the first week (for the first consultation), and end of the first month (for the third); the second control should be performed within the period between the first and third visit, according to the state of health of the woman.  - **Newborn:**  Control visits at 7 days and at 28 days.  For the evaluation of this indicator, it will only be taken into account that the mother has been informed of the first visit for her and her newborn.  SCC validation method: independent measurement of the SCC and the results of the survey to mothers. | |

| TECHNICAL DATE OF INDICATORS: PROCESS | |
| --- | --- |
| **GROUP:** | Good practices |
| **AREA:** | Before Exit |
| Sub-area | Care of the newborn |
| **Nº** | 1 (of 1) |
| **INDICATOR NAME** | Information on the signs of alarm of the newborn |
| **FORM OF MEASUREMENT** | Sampling of files of women attended for reasons of childbirth;  Conducting a survey  SCC |
| **DESCRIPTION** | Percentage of women and / or companions who were informed about the newborn's warning signs before returning to a health unit and asking for help |
| **Level of evidence; Force recommendation** | Unidentified |
| **INDICATOR FORMULA** | Number of women and / or companion who were informed of the newborn's warning signs |
| **Numerator** |  |
| **Denominator** | Number of newborns graduating |
| **DATA SOURCE** | Review of the SCC  Questionnaire for woman / companion |
| **INDICATOR ELABORATION** | Individual |
| **BIBLIOGRAPHIC REFERENCES**  World Health Organization (WHO). WHO - Safe childbirth checklist [Internet]. Available from:  <http://www.who.int/patientsafety/implementation/checklists/childbirth/en/> | |
| **OBSERVATIONS**  The alarm signs of the newborn before whom the help described in the SCC (for each sign and of the set -compound indicator type 100% of compliance) should be evaluated:  -Fever  - Rapid breathing or shortness of breath  -Extremely cold  - Do not urinate or evacuate  - Cyanosis  - Interruption of correct power supply Number of SCC reviewed  -Convulsive crisis  - Less activity than normal  - Jaundice  - Rejection orally or vomiting  Information validation tool: mothers survey | |

# 4. Outcome indicators

| TECHNICAL INDICATOR: RESULT | |
| --- | --- |
| **GROUP:** | Results in health |
| **AREA:** | Complications (morbidity) |
| Sub-area | Mother |
| **Nº** | 1 (of 3) |
| **INDICATOR NAME** | Incidence of obstetric hemorrhage |
| **FORM OF MEASUREMENT** | Sampling of files of women who were attended for reasons of childbirth |
| **DESCRIPTION** | Percentage of women suffered bleeding (biweekly periods) |
| **Level of evidence; Force recommendation** | Not applicable |
| **INDICATOR FORMULA** | Number of women presenting with intra- and postpartum haemorrhage |
| **Numerator** |  |
| **Denominator** | Number of women attended due to labor |
| **DATA SOURCE** | Clinic history  SCC |
| **INDICATOR ELABORATION** | Individual |
| **BIBLIOGRAPHIC REFERENCES**   - Secretaría de Salud. Diagnóstico y tratamiento de la hemorragia obstétrica en la segunda mitad del embarazo y puerperio inmediato. IMSS-162-09. [Internet]. México; 2009. Available from: <http://www.cenetec.salud.gob.mx/interior/catalogoMaestroGPC.html> - Instituto Mexicano de la Seguridad Social (IMSS). Guía de práctica clínica. Detección y tratamiento inicial de las emergencias obstétricas. IMSS-436-11. [Internet]. México; 2011. Available from: <http://www.cenetec.salud.gob.mx/interior/catalogoMaestroGPC.html> | |
| **OBSERVATIONS**  ▪ Severe obstetric haemorrhage: Obstetric blood loss, with any of the following criteria: loss of 25% of blood volume, drop in hematocrit greater than 10 points, presence of hemodynamic changes or loss greater than 150 ml / min.  ▪ Postpartum haemorrhage (75% of cases of pathological puerperium): blood loss of more than 500 ml after vaginal delivery, or loss of more than 1000 ml after cesarean section. It has also been defined as the decrease in hematocrit level of 10%. It is considered as primary, if it occurs within the first 24 hours after birth and its main cause is uterine atony (80% of cases).  Alternatively, we will try to obtain the data of the monthly statistics that the center reports, as well as the total number of cases recorded in electronic databases with the ICD 10:  O67: Labor and delivery complicated by intrapartum hemorrhage, not elsewhere classified  O71: Another obstetric trauma  O72: Postpartum haemorrhage | |

| TECHNICAL INDICATOR: RESULT | |
| --- | --- |
| **GROUP:** | Results in health |
| **AREA:** | Complications (morbidity) |
| Sub-area | Mother |
| **Nº** | 2 (of 3) |
| **INDICATOR NAME** | Incidence of pre-, intra- and postpartum blood pressure disorders |
| **FORM OF MEASUREMENT** | Sampling of files of women who were attended for reasons of childbirth |
| **DESCRIPTION** | Percentage of women with blood pressure disorders in the birthing process (fortnightly periods) |
| **Level of evidence; Force recommendation** | Not applicable |
| **INDICATOR FORMULA** | Number of women with pre-, intra- and postpartum blood pressure disorders |
| **Numerator** |  |
| **Denominator** | Number of women attended due to labor |
| **DATA SOURCE** | Clinical history - SCC - Partograma |
| **INDICATOR ELABORATION** | Individual |
| **BIBLIOGRAPHIC REFERENCES**   - Secretaría de Salud. Detección y Diagnóstico de Enfermedades Hipertensivas del Embarazo. (México D. F., 2010).   <http://www.cenetec.salud.gob.mx/interior/catalogoMaestroGPC.html>   - Secretaría de Salud. Guía de Práctica Clínica Intervenciones de Enfermería en la paciente con Preeclampsia/Eclampsia. (México, 2011).   <http://www.cenetec.salud.gob.mx/interior/catalogoMaestroGPC.html>   - Secretaría de Salud. Detección y tratamiento inicial de las emergencias obstétricas. (México, 2011).   <http://www.cenetec.salud.gob.mx/interior/catalogoMaestroGPC.html> | |
| **OBSERVATIONS**  ▪ Preeclampsia: multisystemic syndrome of pregnancy and puerperium. It occurs after the 20th week of gestation, during delivery or in the first two weeks after delivery. Hypertension ≥ 140/90 mmHg and proteinuria, frequent with headache, tinnitus, phosphenes, edema, abdominal pain and / or laboratory alterations.  - Mild: PAS ≥ 140 mmHg and / or PAD ≥ 90 mmHg in normotensive women (2 shots with 6 hour difference between them, and a maximum period of 7 days). Proteinuria ≥300mg in 24-hour urine collection or test strip report of at least 30 mg / dl (1+) in 2 urine samples taken at random (difference of 6 hours between them, maximum period of 7 days and no evidence of urinary tract infection.  - Severe: PA≥ 160/110 mmHg in at least 2 determinations with a minimum of 6 hours difference. Proteinuria of 5 g or more in a 24-hour urine collection (by 3+ reagent strip or more in 2 samples with at least 4 hours difference). Other features: oliguria (less than 500 ml urine in 24 hours), visual disturbances, pulmonary edema, cyanosis, epigastric pain or right hypochondrium, altered liver function tests (DHL increase> 600 IU, double AST elevation and ALT), serum creatinine> 1.2 mg / dl, thrombocytopenia (platelets <150,000 cells / mm3), intrauterine growth restriction.  ▪ Eclampsia: seizures or coma in patients with preeclampsia after the 20th week of gestation, labor or in the first 6 weeks after gestation, in the absence of other causes of seizures.  ▪ HELLP syndrome (atypical variant of severe preeclampsia): microangiopathic haemolysis, elevation of liver enzymes and thrombocytopenia.  The pilot will try: Collect the data of the monthly statistics that the center reports with the total of the cases to compare by CIE 10.  O11, O14, O15, O16 | |

| TECHNICAL INDICATOR: RESULT | |
| --- | --- |
| **GROUP:** | Results in health |
| **AREA:** | Complications (morbidity) |
| Sub-area | Mother |
| **Nº** | 3 (of 3) |

| **INDICATOR NAME** | Incidence of maternal postpartum or perinatal infection |
| --- | --- |
| **FORM OF MEASUREMENT** | Sampling of files of women who were attended for reasons of childbirth |
| **DESCRIPTION** | Percentage of women with perinatal or postpartum infection (biweekly periods) |
| **Level of evidence; Force recommendation** | Not applicable |
| **INDICATOR FORMULA** | Number of women with perinatal or postpartum infection |
| **Numerator** |  |
| **Denominator** | Number of women attended due to labor |
| **DATA SOURCE** | Clinic history  SCC |
| **INDICATOR ELABORATION** | Individual |
| **BIBLIOGRAPHIC REFERENCES**  ^1^Organización Mundial de la Salud. Guía de la OMS para la aplicación de la CIE-10 a las muertes ocurridas durante el embarazo, parto y puerperio: CIE MM. Ginebra 2012.ISBN 978 92 4 154845 8 | |
| **OBSERVATIONS**  Consideration will be given to cases in which your clinical file includes clinical criteria (agreed between participating centers) or infection register (antibiotic prescription and cause) in SCC as identification of numerator units.  The pilot will try to: Collect the data of the monthly statistics that the center reports with the total of the cases to contrast.  Alternatively, CIE10 diagnostic codes will be used for the identification of cases, if possible their exploitation in electronic databases, and will be requested the data of the monthly statistics that the center reports.  CIE Codes 101:  O75.3: Other infection during labor  O85: Puerperal sepsis  O86: Other puerperal infections  If the pilot is unable to obtain the infection records, the criterion is replaced by "antibiotic prescribed" | |

| TECHNICAL INDICATOR: RESULT | |
| --- | --- |
| **GROUP:** | Results in health |
| **AREA:** | Complications (morbidity) |
| Sub-area | Newborn |
| **Nº** | 1 (of 2) |
| **INDICATOR NAME** | Incidence of neonatal infection |
| **FORM OF MEASUREMENT** | Sampling of records of women who were cared for on the basis of delivery or records of newborns |
| **DESCRIPTION** | Percentage of neonates with neonatal infection (biweekly periods) |
| **Level of evidence; Force recommendation** | Not applicable |
| **INDICATOR FORMULA** | Number of infants with infection |
| **Numerator** |  |
| **Denominator** | Live newborn number |
| **DATA SOURCE** | Clinical history of the newborn  SCC |
| **INDICATOR ELABORATION** | Individual |
| **BIBLIOGRAPHIC REFERENCES**  Not applicable | |
| **OBSERVATIONS**  Specific mention in clinical file of diagnosis of infection or reason for antibiotic prescription  The pilot will try to: Collect the data of the monthly statistics that the center reports with the total of the cases to contrast.  Alternatively, CIE10 codes may be used to identify the cases, if there is an electronic record that permits it.  P36 - Bacterial sepsis of the newborn  P37 - Other congenital infectious and parasitic diseases  P38 - Onfalitis of the newborn with or without hemorrhage  P39 - Other specific infections of the perinatal period  If the pilot is unable to obtain the infection records, the criterion is replaced by "antibiotic prescribed" | |

| TECHNICAL INDICATOR: RESULT | |
| --- | --- |
| **GROUP:** | Results in health |
| **AREA:** | Complications (morbidity) |
| Sub-area | Newborn |
| **Nº** | 2 (of 2) |
| **INDICATOR NAME** | Incidence of neonatal asphyxia |
| **FORM OF MEASUREMENT** | Sampling of records of women who were cared for on the basis of delivery or records of newborns |
| **DESCRIPTION** | Percentage of neonates with neonatal asphyxia (fortnightly periods) |
| **Level of evidence; Force recommendation** | Not applicable |
| **INDICATOR FORMULA** | Number of neonates with neonatal asphyxia event |
| **Numerator** |  |
| **Denominator** | Live newborn number |
| **DATA SOURCE** | Clinical history of the newborn |
| **INDICATOR ELABORATION** | Individual |
| **BIBLIOGRAPHIC REFERENCES**  Not applicable | |
| **OBSERVATIONS**  Specific mention of neonatal asphyxia in the clinical file  Collect the data of the monthly statistics that the center reports with the total of the cases to contrast.  P20 - Intrauterine hypoxia (rule out other diagnoses)  P21 - Asphyxia in childbirth | |

| TECHNICAL INDICATOR: RESULT | |
| --- | --- |
| **GROUP:** | Results in health |
| **AREA:** | Interventions |
| Sub-area |  |
| **Nº** | 1 (of 3) |
| **INDICATOR NAME** | Percentage of deliveries with caesarean section |
| **FORM OF MEASUREMENT** | Sampling of files of women who were attended for reasons of childbirth |
| **DESCRIPTION** | Percentage of women with cesarean delivery |
| **Level of evidence; Force recommendation** | Not applicable |
| **INDICATOR FORMULA** | Number of women undergoing caesarean section |
| **Numerator** |  |
| **Denominator** | Number of women attended due to labor |
| **DATA SOURCE** | Clinical records  SCC (if applicable) |
| **INDICATOR ELABORATION** | Individual |
| **BIBLIOGRAPHIC REFERENCES**  Norma Oficial Mexicana NOM-007-SSA2-1993, Atención de la mujer durante el embarazo, parto y puerperio y del recién nacido. Criterios y procedimientos para la prestación del servicio. | |
| **OBSERVATIONS**  All medical units with obstetric care should have guidelines for cesarean indication, which is ideally recommended for 15% in second level hospitals and 20% for third level hospitals in relation to total births. health care units should approach these values. | |

| TECHNICAL INDICATOR: RESULT | |
| --- | --- |
| **GROUP:** | Results in health |
| **AREA:** | Interventions |
| Sub-area |  |
| **Nº** | 2 (of 3) |
| **INDICATOR NAME** | Percentage of instrumented deliveries |
| **FORM OF MEASUREMENT** | Sampling of files of women who were attended for reasons of childbirth |
| **DESCRIPTION** | Percentage of women with instrumented delivery resolutio |
| **Level of evidence; Force recommendation** | Not applicable |
| **INDICATOR FORMULA** | Number of women undergoing instrumented delivery |
| **Numerator** |  |
| **Denominator** | Number of women attended due to labor |
| **DATA SOURCE** | Clinical records  SCC (if applicable) |
| **INDICATOR ELABORATION** | Individual |
| **BIBLIOGRAPHIC REFERENCES**  Guía de Práctica Clínica para la Reducción de la Frecuencia de Operación Cesárea México: Instituto Mexicano de Seguro social; 2014. ISBN: 978-607-7790-92-1 | |
| **OBSERVATIONS** | |

| TECHNICAL INDICATOR: RESULT | |
| --- | --- |
| **GROUP:** | Results in health |
| **AREA:** | Interventions |
| Sub-area |  |
| **Nº** | 3 (of 3) |
| **INDICATOR NAME** | Percentage of deliveries with episiotomy |
| **FORM OF MEASUREMENT** | Sampling of files of women who were attended for reasons of childbirth |
| **DESCRIPTION** | Percentage of women with episiotomy at delivery |
| **Level of evidence; Force recommendation** | Not applicable |
| **INDICATOR FORMULA** | Number of women with episiotomy at delivery |
| **Numerator** |  |
| **Denominator** | Number of women attended due to labor |
| **DATA SOURCE** | Clinical records  SCC (if applicable |
| **INDICATOR ELABORATION** | Individual |
| **BIBLIOGRAPHIC REFERENCES**   - - - 1. Informe presentado por el Grupo Técnico de Trabajo - OMS. CUIDADOS EN EL PARTO NORMAL: UNA GUÍA PRÁCTICA. Ginebra; 1996.       2. Belizan J, Campodonico L, Carroli G, Gonzalez L. **Routine vs selective episiotomy: a randomised controlled trial**. *Lancet*. 1993; **18**:1517-8.       3. Hartmann K, Viswanathan M, Palmieri R, Gartlehner G, Thorp J, Lohr KN. **Outcomes of routine episiotomy: a systematic review**. *JAMA*. 2005; **17**:2141-8. | |
| **OBSERVATIONS**  The 1996 WHO report mentions that following a study (Sleep et al 1984), a good target might be an episiotomy frequency of no more than 10% 1 for normal deliveries. Subsequently, different authors have recommended that the performance of episiotomy should be between 30% and 15% 2,3, but in no case should exceed 30% 2. | |

| TECHNICAL INDICATOR: RESULT | |
| --- | --- |
| **GROUP:** | Results in health |
| **AREA:** | Adverse events |
| Sub-area | Mother |
| **Nº** | 1 (of 1) |
| **INDICATOR NAME** | Adverse events in the mother |
| **FORM OF MEASUREMENT** | Sampling of files of women who were attended for reasons of childbirth |
| **DESCRIPTION** | Percentage of adverse events in women given birth |
| **Level of evidence; Force recommendation** | Not applicable |
| **INDICATOR FORMULA** | Number of women with at least one adverse event |
| **Numerator** |  |
| **Denominator** | Total deliveries |
| **DATA SOURCE** | Medical records |
| **INDICATOR ELABORATION** | Adapted from Pettker et al. |
| **BIBLIOGRAPHIC REFERENCES**  Pettker CM et al. Impact of a comprehensive patient safety strategy on obstetric adverse events. Am Journal Obst Gynecol, 2009, 200:492e1-492e8. Disponible en: http://www.ajog.org/article/S0002-9378(09)00092-1/pdf. | |
| **OBSERVATIONS**  All adverse events listed shall be taken into account, but only one adverse event per woman is considered:  -Blood transfusion  - 3rd or 4th degree laceration  - Internships in ICU  - Postpartum hysterectomy  - Uterine rupture  - Return to hospital after discharge  - Maternal death  The use of a composite indicator facilitates the obtaining of a result of adverse events, minimizing the low frequency of some of them, and individually indicating the severity of the cases, since the highest is the numerator of the indicator of adverse events probably more failures in the service existed the childbirth. | |

| TECHNICAL INDICATOR: RESULT | |
| --- | --- |
| **GROUP:** | Results in health |
| **AREA:** | Morbi-mortality |
| Sub-area | Newborn |
| **Nº** | 1 (of 1) |
| **INDICATOR NAME** | Adverse events in newborns |
| **FORM OF MEASUREMENT** | Sampling of records of women who were cared for by the birth or newborn |
| **DESCRIPTION** | Percentage of adverse events in newborn infants treated at the center |
| **Level of evidence; Force recommendation** | Not applicable |
| **INDICATOR FORMULA** | Number of newborns with at least one adverse effect |
| **Numerator** |  |
| **Denominator** | Total births |
| **DATA SOURCE** | Medical records |
| **INDICATOR ELABORATION** | Adapted from Pettker et al. |
| **BIBLIOGRAPHIC REFERENCES**  Pettker CM et al. Impact of a comprehensive patient safety strategy on obstetric adverse events. Am Journal Obst Gynecol, 2009, 200:492e1-492e8. Disponible en: http://www.ajog.org/article/S0002-9378(09)00092-1/pdf. | |
| **OBSERVATIONS**  All adverse events listed will be considered, but only one adverse event per newborn is considered:  - NICU interns weighing> 2500g and for> 24 hours;  - APGAR <7 at 5 minutes  Trauma or wounding in childbirth (eg, head injury, fracture, neurological injury, bleeding or laceration);  - Hospitalized more than 7 days  - Fetal or neonatal death  The use of a composite indicator facilitates the obtaining of a result of adverse events, minimizing the low frequency of some of them, and individually indicating the severity of the cases, since the highest is the numerator of the indicator of adverse events probably more failures in the service existed the childbirth. | |
